# Resveratrol Enhances Antitumor Activity of TRAIL in Prostate Cancer Xenografts through Activation of FOXO Transcription Factor

**DOI:** 10.1371/journal.pone.0015627

**Published:** 2010-12-28

**Authors:** Suthakar Ganapathy, Qinghe Chen, Karan P. Singh, Sharmila Shankar, Rakesh K. Srivastava

**Affiliations:** 1 Division of Radiation Biology, Department of Radiation Oncology, The University of Texas Health Science Center at San Antonio, Greehey Children's Cancer Research Institute, San Antonio, Texas, United States of America; 2 Virginia Bioinformatics Institute, Virginia Polytechnic Institute and State University, Blacksburg, Virginia, United States of America; 3 Department of Biostatistics, University of North Texas Health Science Center at Fort Worth, Fort Worth, Texas, United States of America; 4 Department of Pathology and Laboratory Medicine, The University of Kansas Cancer Center, The University of Kansas Medical Center, Kansas City, Kansas, United States of America; 5 Department of Pharmacology, Toxicology and Therapeutics, and Medicine, The University of Kansas Cancer Center, The University of Kansas Medical Center, Kansas City, Kansas, United States of America; The Ohio State University Medical Center, United States of America

## Abstract

**Background:**

Resveratrol (3, 4′, 5 tri-hydroxystilbene), a naturally occurring polyphenol, exhibits anti-inflammatory, antioxidant, cardioprotective and antitumor activities. We have recently shown that resveratrol can enhance the apoptosis-inducing potential of TRAIL in prostate cancer cells through multiple mechanisms *in vitro*. Therefore, the present study was designed to validate whether resveratrol can enhance the apoptosis-inducing potential of TRAIL in a xenograft model of prostate cancer.

**Methodology/Principal Findings:**

Resveratrol and TRAIL alone inhibited growth of PC-3 xenografts in nude mice by inhibiting tumor cell proliferation (PCNA and Ki67 staining) and inducing apoptosis (TUNEL staining). The combination of resveratrol and TRAIL was more effective in inhibiting tumor growth than single agent alone. In xenografted tumors, resveratrol upregulated the expressions of TRAIL-R1/DR4, TRAIL-R2/DR5, Bax and p27^/K IP1^, and inhibited the expression of Bcl-2 and cyclin D1. Treatment of mice with resveratrol and TRAIL alone inhibited angiogenesis (as demonstrated by reduced number of blood vessels, and VEGF and VEGFR2 positive cells) and markers of metastasis (MMP-2 and MMP-9). The combination of resveratrol with TRAIL further inhibited number of blood vessels in tumors, and circulating endothelial growth factor receptor 2-positive endothelial cells than single agent alone. Furthermore, resveratrol inhibited the cytoplasmic phosphorylation of FKHRL1 resulting in its enhanced activation as demonstrated by increased DNA binding activity.

**Conclusions/Significance:**

These data suggest that resveratrol can enhance the apoptosis-inducing potential of TRAIL by activating FKHRL1 and its target genes. The ability of resveratrol to inhibit tumor growth, metastasis and angiogenesis, and enhance the therapeutic potential of TRAIL suggests that resveratrol alone or in combination with TRAIL can be used for the management of prostate cancer.

## Introduction

Resveratrol (3, 4', 5 tri-hydroxystilbene), a naturally occurring polyphenol, exhibits pleiotropic health benefits including anti-inflammatory, antioxidant, cardioprotective and antitumor activities [1], [2], [3], [4]. Currently, numerous preclinical findings suggest resveratrol as a promising agent for cancer prevention and/or treatment. As a potential anti-cancer agent, resveratrol has been shown to inhibit or retard the growth of various cancer cells *in vitro* and implanted tumors in vivo [5], [6], [7], [8], [9]. Resveratrol has been shown to inhibit the activation of JAK2-STAT3, Src-STAT3, AKT and IKK-NFκB pathways and to induce apoptosis in several cancer cell lines [10], [11], [12], [13]. We have recently demonstrated that resveratrol downregulated the expression of Bcl-2, Bcl-X_L_ and survivin and upregulated the expression of Bax, Bak, PUMA, Noxa, and Bim and death receptors (TRAIL-R1/DR4 and TRAIL-R2/DR5) [1], [14], [15]. Furthermore, Treatment of prostate cancer cells with resveratrol resulted in generation of reactive oxygen species (ROS), translocation of Bax to mitochondria and subsequent drop in mitochondrial membrane potential, release of mitochondrial proteins (cytochrome c, Smac/DIABLO, and AIF) to cytosol, activation of effector caspase-3 and caspase-9, and induction of apoptosis [1], [14], [15]. Resveratrol-induced ROS production, caspase-3 activity and apoptosis were inhibited by N-acetylcysteine, suggesting the ROS production, at least in part, plays a role in mediating anticancer activities of resveratrol [1], [14], [15]. Resveratrol enhanced the apoptosis-inducing potential of TRAIL in PC-3 cells and sensitized TRAIL-resistant prostate cancer LNCaP cells *in vitro* [1], [14], [15]. Overall, these data suggest that resveratrol can regulate multiple signaling pathways and possesses several therapeutic benefits.

PI3K signaling plays a pivotal role in intracellular signal transduction pathways involved in cellular transformation, cell growth, and tumorigenesis [16], [17]. Inactivation of AKT results in dephosphorylation and activation of FOXO transcription factors, reported to mediate cell cycle arrest, DNA repair, and apoptosis [18], [19]. These transcription factors, belong to the ‘O’ subgroup of winged-helix/forkhead transcription-factor family, consist principally of four members FOXO1, FOXO3a, FOXO4, and FOXO6 [20]. FOXO proteins are evolutionarily conserved transcription factors implicated in several fundamental cellular processes, functioning as end-point for transcriptional programs involved in apoptosis, stress response and longevity [21], [22]. Since abrogation of FOXO function is frequently observed in human cancer, the reactivation of FOXO proteins will be an attractive strategy for cancer therapy and prevention. The FOXO proteins integrate regulatory inputs from a variety of upstream signaling pathways, most importantly in response to growth factor and stress signaling [23]. Recently, FOXO factors have been established as tumor suppressors, promoting the transcription of pro-apoptotic molecules like FasL and Bim when the PI3K/AKT pathway is downregulated due to nutrient or serum starvation and cellular stress [24], [25]. Triple knockout mouse models proved the tumor suppressor properties of FOXOs, as mice simultaneously lacking the principal members of the mammalian FOXO subfamily, FOXO1, FOXO3a and FOXO4, are prone to develop hemangiomas and lymphoproliferative diseases [26]. Conversely, the individual or paired inactivation of FOXO1, FOXO3a or FOXO4 resulted in a less severe phenotype, supporting the idea of functional redundancy of these FOXO factors [26]. Furthermore, forced expression of FOXO has been shown to inhibit tumorigenesis in xenograft models in nude mice [27], [28]. Therefore, reactivation of FOXO based on its tumor suppressor properties is considered as a very attractive anti-cancer strategy. Since FOXO proteins were reported to be critical mediators of apoptosis induced by anticancer drugs, we postulated that FOXO expression or transcriptional activity could be important event in mediating the effects of resveratrol.

TNF-related apoptosis-inducing ligand (TRAIL) has been shown to TRAIL-R1/DR4 and TRAIL-R2/DR5 [29]. We and others have shown that TRAIL can induce apoptosis in various cancer cell types [29], [30], [31], [32], [33]. Based on preclinical data, it appears that TRAIL has great promise as a selective anticancer agent [31], [32]. Resveratrol has been shown to enhance the therapeutic potential of TRAIL *in vitro* [14], [34]. The interactions of resveratrol and TRAIL were blocked by either dominant negative FADD or caspase-8 siRNA [14], [15]. The combination of resveratrol and TRAIL enhanced the mitochondrial dysfunctions during apoptosis. Resveratrol treatment can activate the extrinsic TRAIL-receptor-mediated death pathway, thereby increasing sensitivity to TRAIL in prostate cancer cells. However, the molecular mechanisms by which resveratrol can enhance the therapeutic potential of TRAIL *in vivo* has not been examined.

The purpose of our study was to investigate the molecular mechanisms by which resveratrol enhances the therapeutic potential of TRAIL in prostate cancer xenografts in nude mice. Our results indicated that resveratrol inhibited PC-3 xenograft growth and markers of metastasis, and angiogenesis through activation of FOXO transcription factors. Thus, our data suggest that resveratrol can be used alone or in combination with TRAIL for the management of of prostate cancer.

## Results

### Resveratrol enhances antitumor tumor activity of TRAIL in PC-3 xenografts *in vivo*

**Figure 1:**
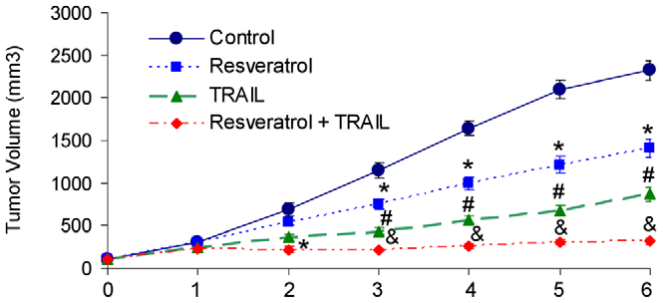
Resveratrol enhances apoptosis-inducing potential of TRAIL in PC-3 xenografts. (A), PC-3 cells were injected into the right flanks of Balb c nude mice. After tumor formation (about 100 mm^3^), mice were treated with saline, resveratrol (30 mg/kg, three days per week), TRAIL (15 mg/kg, four times during first three weeks) or resveratrol plus TRAIL. Tumor volume was measured weekly. Data represent mean ± SE. *, # and $ are significantly different from control, P<0.05).

We have recently shown that resveratrol can enhance the apoptosis inducing potential of TRAIL *in vitro*. Therefore, in the present study, we examined whether resveratrol can enhance the antitumor activity of TRAIL *in vivo*. PC-3 cells were xenografted in Balb c nude mice. After tumor formation, these mice were treated with resveratrol, TRAIL, or resveratrol plus TRAIL for 6 weeks and the effects of these agents on tumor growth were examined. Resveratrol and TRAIL alone inhibited growth of PC-3 xenografts (Fig. 1). By comparison, the combination of resveratrol and TRAIL was more effective in inhibiting tumor growth than single agent alone.

These data suggest that resveratrol can enhance the antitumor activity of TRAIL in prostate cancer.

### Regulation of tumor cell proliferation and apoptosis by resveratrol and/or TRAIL in PC-3 xenografts

**Figure 2:**
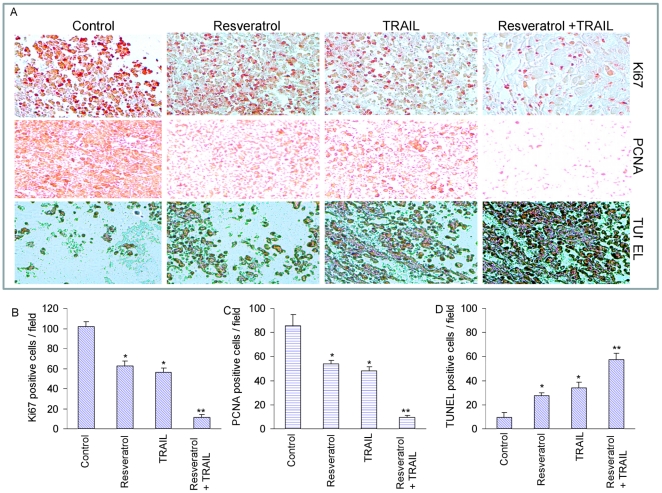
Effects of resveratrol and/or TRAIL on tumor cell proliferation and apoptosis. (A), Immunohistochemistry was performed in tumor tissues derived from control, resveratrol and/or TRAIL treated mice on week 6 to measure cell proliferation by Ki67 and PCNA staining and apoptosis by TUNEL assay. (B, C and D), Quantification of Ki67, PCNA and TUNEL positive tumor cells. Tumor slides of different treatment groups were visualized under microscope, and Ki67, PCNA and TUNEL positive cells were quantified. Data represent mean ± SE. * and ** are significantly different from their respective controls, P<0.05).

Tumor cell proliferation and apoptosis can regulate the size of tumor at any given time. Therefore, we performed immunohistochemistry in tumor tissues to measure the expression of Ki67 and PCNA, and TUNEL assay to measure apoptosis (Fig. 2A). Tumor cell proliferation was measured by counting Ki67 and PCNA positive cells, and apoptosis was measured by counting TUNEL positive cells (Fig. 2B–D). Treatment of mice with resveratrol and TRAIL alone resulted in inhibition of tumor cell proliferation, and induction in apoptosis. The combination of resveratrol and TRAIL was more effective in inhibiting tumor cell proliferation and inducing apoptosis than single agent alone. These data suggest that resveratrol, although effective alone, can enhance the antitumor activity of TRAIL in prostate cancer.

### *In vivo* regulation of death receptor TRAIL-R1/DR4 and TRAIL-R2/DR5 by resveratrol and/or TRAIL

**Figure 3:**
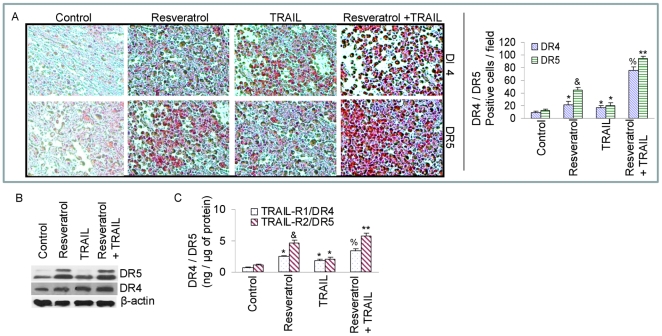
Effects of resveratrol and/or TRAIL on the expression of TRAIL-death receptors. (A), Immunohistochemistry was performed to measure the expressions of TRAIL-R1/DR4 and TRAIL-R2/DR5 in tumor tissues derived from control and treated mice on week 6. Quantification of DR4 and DR5 positive cells are also shown on right panel. (B), Expressions of TRAIL-R1/DR4, TRAIL-R2/DR5 and β-actin in tumor tissues derived on week 6. Western blot analysis was performed to measure the expression of DRs (left panel). Quantification of DR4 and DR5 positive tumor cells (right panel). (C), Measurement of DR4 and DR5 by ELISA. Proteins extracts were prepared and the expressions of DRs were measured as per manufacturer's instructions.

Since resveratrol enhances the therapeutic potential of TRAIL by inducing apoptosis *in vivo*, we sought to examine the molecular mechanisms by which resveratrol enhances the antitumor activity of TRAIL in PC-3 xenografts. We next examined the effects of resveratrol and/or TRAIL on the expression of death receptors (TRAIL-R1/DR4 and TRAIL-R2/DR5) by immunohistochemistry in tumor tissues derived from *in vivo* experiment (Fig. 3A, left and right panels). Treatment of mice with resveratrol enhanced the expressions of DR4 and DR5. TRAIL slightly induced the expression of DR4 and DR5. On the other hand, treatment of mice with a combination of resveratrol plus TRAIL significantly showed enhanced expressions of DR4 and DR5 proteins than that of mice treated with resveratrol alone or TRAIL alone.

We confirmed the immunohistochemistry data by examining the expression of these proteins by the Western blot analysis (Fig. 3B). Treatment of mice with Resveratrol and TRAIL alone resulted in upregulation of death receptors DR4 and DR5. By comparison, resveratrol plus TRAIL treatments had more effects on the induction of DR4 and DR5 compared to single agent alone. These data are in agreement with immunohistochemistry data where the proapoptotic DR4 and DR5 proteins were upregulated.

We next confirmed the immunohistochemistry and western blot data by examining the expressions of DRs by ELISA (Fig. 3C, right panel). Treatment of mice with resveratrol and TRAIL alone upregulated the expression of death receptors (DR4 and DR5). By comparison, the combination of resveratrol plus TRAIL induced more DR4 and DR5 expressions than single agent alone. These data are in agreement with immunohistochemistry and western blot data where the proapoptotic DR4 and DR5 proteins were upregulated by resveratrol and TRAIL. Up-regulation of DRs may enhance the apoptosis-inducing potential of TRAIL.

### *In vivo* regulation of Bcl-2 family members and cell cycle regulatory proteins by resveratrol and/or TRAIL

**Figure 4:**
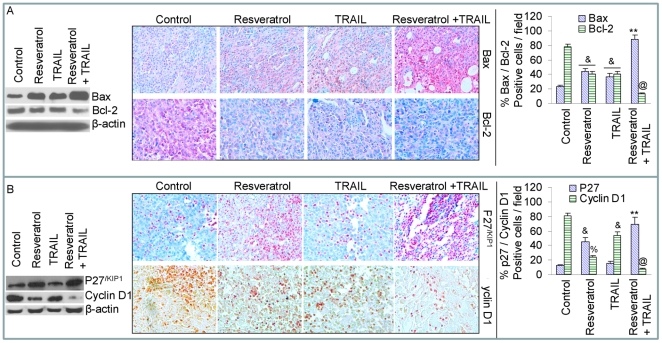
Effects of resveratrol and/or TRAIL on Bcl-2 family members and cell cycle regulatory proteins. (A), Western blot analysis was performed to measure the expressions of Bax and Bcl-2 in tumor tissues derived from control, resveratrol and/or TRAIL treated mice on week 6 (left panel). Immunohistochemistry was performed to measure the expressions of Bax and Bcl-2 in tumor tissues derived from control and drug treated mice on week 6 (middle panel). Quantification of Bax and Bcl-2 positive cells in tumor cells (right panel). (B), Western blot analysis was performed to measure the expressions of p27^/KIP1^ and cyclin D1 in tumor tissues derived from control and drug treated mice on week 6 (left panel). Immunohistochemistry was performed to measure the expressions of p27^/KIP1^ and cyclin D1 in tumor tissues derived from control and drug treated mice on week 6 (middle panel). Quantification of p27^/KIP1^ and cyclin D1 positive cells in tumor tissues (right panel).

Since Bcl-2 family members play a major role in apoptosis, we sought to examine the expression of Bax and Bcl-2 in tumor tissues derived from resveratrol and/or TRAIL-treated mice (Fig. 4A, left panel). Treatment of mice with resveratrol and TRAIL alone resulted in upregulation of Bax, and inhibition of Bcl-2 expression. By comparison, the combination of resveratrol plus TRAIL was more effective in upregulating Bax and inhibiting Bcl-2. We next confirmed the Western blot data by examining the expressions of these proteins by immunohistochemistry (Fig. 4A, middle and right panels). Treatment of mice with resveratrol upregulated the expression of Bax and inhibited the expression of Bcl-2. By comparison, treatment of mice with a combination of resveratrol plus TRAIL had more effects on the upregulation of Bax and inhibition of Bcl-2 than single agent alone.

We next examined the effects of resveratrol and/or TRAIL on the expression of p27^/KIP1^ and cyclin D1 in tumor tissues by Western blotting and immunohistochemistry (Fig. 4B). Treatment of mice with resveratrol resulted in the induction of cell cycle inhibitor p27^/KIP1^ and inhibition of cyclin D1 expression (Fig. 4B). By comparison, TRAIL had no significant effect on p27 expression, but slightly inhibited the expression of cyclin D1 compared to control group. However, the combination of resveratrol and TRAIL showed upregulation of p27, and inhibition of cyclin D1 compared to single agent alone. We next confirmed the Western blot data with immunohistochemistry data by examining the expressions of these proteins (Fig. 4B, middle and right panels). TRAIL has no significant effect on the expression of p27 but slightly inhibited the expression of cyclin D1. By comparison, resveratrol induced the expressions of p27, and inhibited the expression of cyclin D1. These data suggest that resveratrol can regulate cell cycle by up-regulating the expression of p27 and inhibiting the expression of cyclin D1.

### *In vivo* regulation of MMP-2 and MMP-9 expression by resveratrol and/or TRAIL

**Figure 5:**
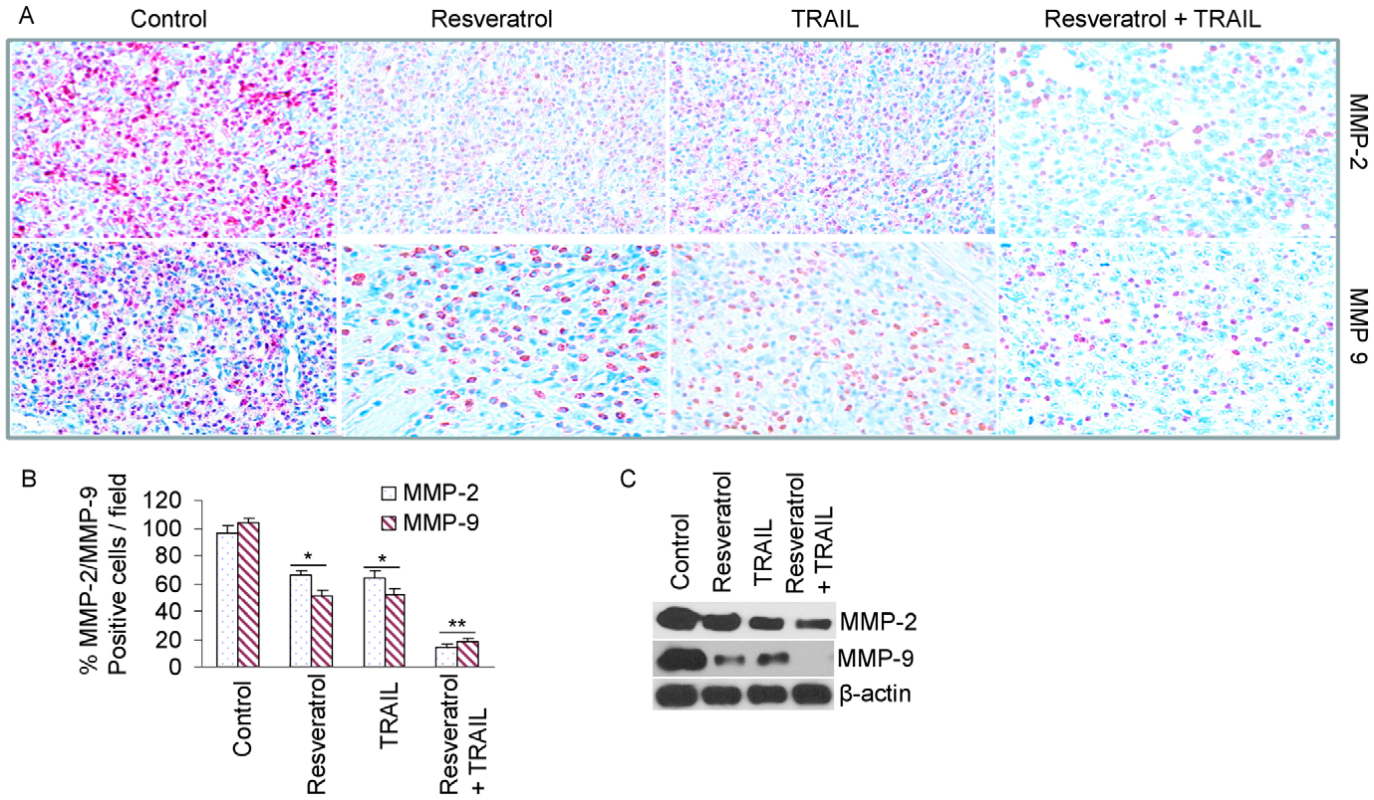
Effects of resveratrol and/or TRAIL on markers of metastasis. (A), Immunohistochemistry was performed to measure the expressions of MMP-2 and MMP-9 in tumor tissues derived from control, resveratrol and/or TRAILtreated mice on week 6. (B), Quantification of MMP-2 and MMP-9 positive cells in tumor tissues. (C), Expressions of MMP-2, MMP-9 and β-actin in tumor tissues were measured on week 6 by the Western blot analysis.

Elevated expressions of matrix metalloproteinases (MMPs) are associated with increased metastatic potential in many tumor cells [35], [36], [37]. We therefore sought to examine the effects of resveratrol on MMP-2 and MMP-9 expressions in tumor tissues derived from xenografted nude mice by immunohistochemistry and Western blot analysis. Treatment of xenogrfated mice with resveratrol resulted in inhibition of MMP-2 and MMP-9 expressions than those of control or TRAIL treated group (Fig. 5A and B). The combination of resveratrol and TRAIL was more effective in inhibiting MMP-2 and MMP-9 expressions than single agent alone. We next confirmed the immunohistochemistry data by examining the expressions of these proteins by Western blot analysis (Fig. 5C). TRAIL has no significant effect on the expressions of MMP-2 and MMP-9. By comparison, resveratrol or resveratrol plus TRAIL inhibited the expressions of MMP-2 and MMP-9. These data suggest that resveratrol and/or TRAIL may inhibit prostate cancer metastasis by inhibiting MMP-2 and MMP-9.

### *In vivo* regulation of angiogenesis by resveratrol and/or TRAIL

**Figure 6:**
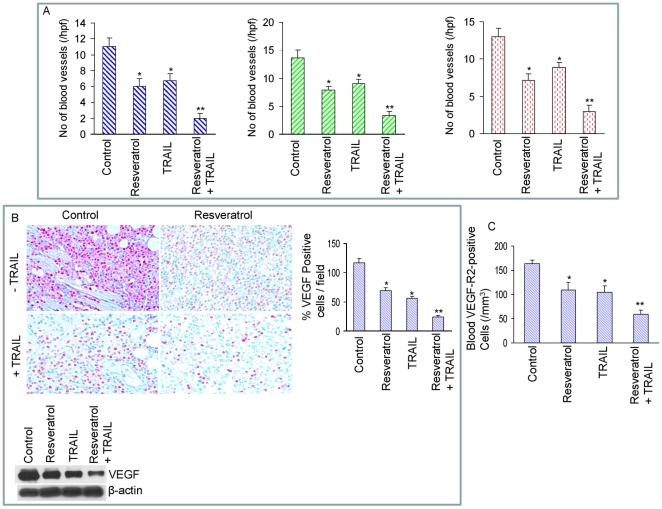
Effects of resveratrol and/or TRAIL on markers of angiogenesis. (A), Left panel, tumor tissue sections derived from control, resveratrol and/or TRAIL treated mice on week 6 were stained with H & E and the numbers of blood vessels were counted at 400 X magnification. Each column represents the mean ± SD. * or **  =  significantly different from control, P<0.05. Middle panel, blood vessel quantification in tumors derived on week 6. Tumor sections from control and drug treated mice were stained with anti-CD31 antibody, and the numbers of CD31-positive blood vessels were counted. The results are shown as the mean ± SD. * or **  =  significantly different from control, P<0.05. Right panel, tumor sections from control and drug treated mice obtained on week 6 were stained with anti-von Willebrand Factor (vWF) antibody to evaluate blood vessels. The results are shown as the mean ± SD. * or **  =  significantly different from control, P<0.05. (B), Left panel, immunohistochemistry was performed to measure the expression of VEGF in tumor tissues derived from control and drug treated mice on week 6. Right panel, quantification of VEGF positive cells in tumor tissues. Right panel, quantification of VEGF positive cells. The results are shown as the mean ± SD. * or **  =  significantly different from control, P<0.05. Bottom panel, expressions of VEGF and β-actin in tumor tissues derived on week 6 were measured by the Western blot analysis. (C), VEGF receptor 2 (VEGF-R2)-positive circulating endothelial cells in mice on week 6. The blood cells from peripheral blood attached to the slide were stained with anti-VEGF-R2 antibody, and the number of VEGF-R2 positive cells was counted under a microscope. The results are shown as the mean ± SD. * or **  =  significantly different from control, P<0.05.

Whether regression in tumor growth by resveratrol and/or TRAIL was due to inhibition of angiogenesis, we analyzed the markers of angiogenesis by immunohistochemistry in tumor tissues derived from control and treated mice (Fig. 6). We first examined the effects of resveratrol and/or TRAIL treatment on number of blood vessels in tumor tissues by utilizing three different approaches (Fig. 6A). Blood vessels were examined by staining the tumor tissues by H&E, anti-CD31 antibody, and anti-vWF antibody. Treatment of mice with resveratrol or TRAIL caused an inhibition in number of blood vessels. By comparison, the treatment of mice with a combination of resveratrol plus TRAIL further inhibited the number of blood vessels.

We next examined the expression of VEGF by immunohistochemistry and Western blot analysis (Fig 6). Examination of tumor tissues by immunohistochemistry showed that control mice had increased VEGF-positive endothelial cells compared to resveratrol or TRAIL treated mice (Fig. 6B, left and right panels). The combination of resveratrol plus TRAIL showed significantly less VEGF staining than single agent alone. We next confirmed the immunohistochemistry data of VEGF expression by examining the protein levels by Western blot analysis (Fig. 6B, lower panel). Treatment of mice with resveratrol and TRAIL alone inhibited VEGF expression. By comparison, resveratrol plus TRAIL inhibited the expression of VEGF.

We have demonstrated that numbers of circulating vascular endothelial growth factor receptor 2 (VEGF-R2)-positive endothelial cells correlate directly with increase in tumor angiogenesis and can serve as *in vivo* indicators of tumor angiogenesis [31], [38]. As expected, control mice had increased circulating VEGF-R2-positive endothelial cells compared to resveratrol treated mice (Fig. 6C). Resveratrol plus TRAIL-treated group demonstrated significantly less VEGFR2-positive cells than that of resveratrol or TRAIL treated group. These data strongly demonstrate that resveratrol can inhibit tumor growth by inhibiting angiogenesis, and may also promote antitumor activity of TRAIL *in vivo*.

### *In vivo* regulation of transcription factor FKHRL1

**Figure 7:**
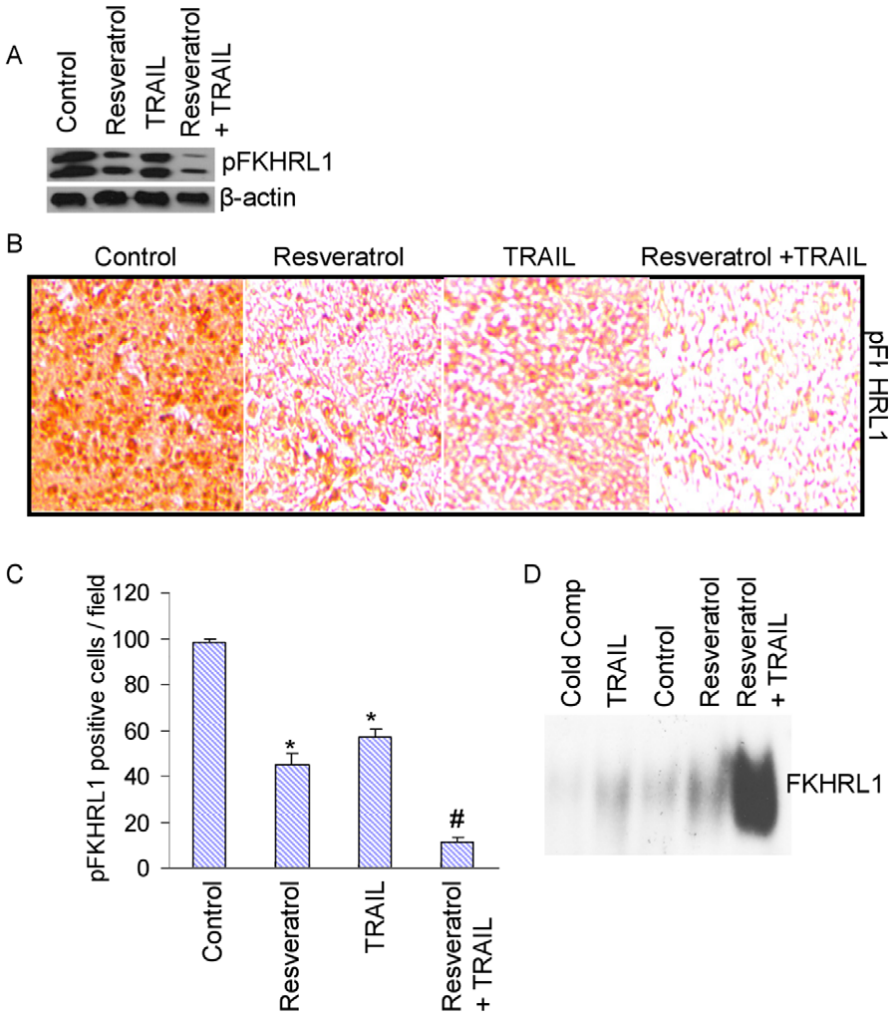
Activation of transcription factor FKHRL1 by resveratrol. (A), Inhibition of FKHRL1 phosphorylation by resveratrol. Western blot analysis was performed to measure the expression of phospho-FKHRL1 in tumor tissues derived from control and/or drug treated mice on week 6. (B), Immunohistochemical examination of phospho-FKHRL1. Immunohistochemistry was performed to measure the expression of phospho-FKHRL1 in tumor tissues derived from control, resveratrol and/or TRAIL treated mice on week 6. (C), Quantification of FKHRL-positive cells in tumor tissues. (D), FKHRL1-DNA binding activity. Nuclear extracts were prepared from tumor tissues derived from control and drug-treated mice. Gelshift assay was performed to measure the FKHRL1-DNA binding activity as described in Materials and Methods.

AKT has been shown to phosphorylate FKHRL1, and the inhibition of FKHRL1 phosphorylation causes its nuclear translocation, enhanced DNA binding and transcriptional activity [39]. We next examined whether antitumor activities of resveratrol and/or TRAIL are exerted through activation of FKHRL1 (Fig. 7). Resveratrol and TRAIL inhibited the phosphorylation of FKHRL1; however, resveratrol was more effective than TRAIL. The combination of resveratrol and TRAIL had more effect in inhibiting phosphorylation of FKHRL1 than single agent alone. We next examined the phosphorylation of FKHRL1 in tumor tissues by immunohistochemistry (Fig. 7B and C). Resveratrol and TRAIL inhibited the cytoplasmic levels of phosphorylated FKHRL1 as shown in Fig. 7B and quantified in Fig. 7C. The combination of resveratrol plus TRAIL had more effect in inhibiting cytoplamic levels of phosphorylated FKHRL1 than single agent alone. These data suggest that resveratrol and TRAIL can inhibit the phosphorylation of FKHRL1, which may results in its nuclear translocation and activation.

We next examined the FKHRL1-DNA binding activity by gelshift assay (Fig. 7D). Nuclear extracts were prepared from tumor tissues derived from resveratrol and/or TRAIL treated mice. Gelshift data revealed that TRAIL and resveratrol enhanced FKHRL1-DNA binding activities. The combination of resveratrol and TRAIL had significantly more FKHRL1-DNA binding activity than single agent alone. Overall, these data suggest that resveratrol and TRAIL can inhibit the phosphorylation of FKHRL1 leading to its enhanced nuclear translocation and DNA binding activities.

## Discussion

We have recently shown that resveratrol induces apoptosis in TRAIL-sensitive PC-3 cells, and sensitizes TRAIL-resistant LNCaP cells *in vitro* through activation of multiple signaling pathways [14], [15]. Resveratrol-induced apoptosis engages mitochondria, as was shown by a drop in mitochondrial membrane potential and activation of caspase-3 and caspase-9 in both prostate cancer PC-3 and LNCaP cells [14], [15]. Resveratrol induced expression of proapoptotic proteins (Bax, Bak, PUMA, Noxa and Bim), death receptors (TRAIL-R1/DR4 and TRAIL-R2/DR5), and inhibited expression of anti-apoptotic proteins (Bcl-2 and Bcl-X_L_) and IAPs (XIAP and survivin). In our recent study, resveratrol regulated the expression of TRAIL, DR4, DR5, Bim, p27^/KIP1^ and cyclin D1 through FOXO transcription factors *in vitro*, and inhibition of FKHRL1, FKHR and AFX by RNAi blocked these affects of resveratrol [40]. In the present study, we have validated our *in vitro* findings and demonstrated that resveratrol and TRAIL alone inhibited the growth of PC-3 xenografts, metastasis and angiogenesis through activation of FOXO transcription factors. Interestingly, the combination of resveratrol and TRAIL had greater effect on the inhibition of tumor growth, metastasis and angiogenesis than either agent alone.

*In vitro* resveratrol downregulated the expressions of Bcl-2 and Bcl-X_L_ and upregulated the expressions of p53, Bax, Bak, PUMA, Noxa, and Bim at mRNA and protein levels in prostate cancer cells [41]. We have also demonstrated that resveratrol upregulated the expression, phosphorylation, and acetylation of p53 in androgen-dependent LNCaP cells [41]. The ability of resveratrol to regulate gene transcription was also evident as it caused acetylation of histone H3 and H4 in LNCaP cells [41]. Furthermore, treatment of LNCaP cells with resveratrol resulted in translocation of Bax and p53 to mitochondria, production of reactive oxygen species, drop in mitochondrial membrane potential, release of mitochondrial proteins (cytochrome c, Smac/DIABLO and Omi/HtrA2), and activation of caspase-3 leading to apoptosis [41]. Furthermore, deletion of Bax and Bak genes completely inhibited resveratrol-induced cytochrome c and Smac/DIABLO release in mouse embryonic fibroblasts [42]. In the present study, treatment of xenografted mice with resveratrol resulted in downregulation of Bcl-2 and up-regulation of Bax. The combination of resveratrol plus TRAIL was more effective in regulating Bcl-2 family members than single agent alone. Our previously published *in vitro* data are in agreement with current *in vivo* studies which demonstrate that resveratrol can engage cell-intrinsic pathway of apoptosis by regulating the expression of Bcl-2 family of proteins.

FOXO transcription factors are tumor suppressors that are inactivated in the majority of human cancers, owing to the overactivation of the PI3K/AKT pathway [43]. FOXO proteins can regulate a variety of genes that influence cell proliferation, survival, metabolism and response to stress [19], [20], [44]. FOXO transcription factors are regulated by synthesis, acetylation, phosphorylation and ubiquitination at three different levels: subcellular localization, stability and transcriptional activity [19], [20], [44]. Upon activation of PI3K/AKT signaling, FOXOs undergo AKT-mediated phosphorylation, which promotes binding to 14-3-3, nuclear export through CRM1 and cytoplasmic sequestration. Under stress conditions or in the absence of growth or survival factors, when the PI3K/AKT pathway is inhibited, FOXO proteins translocate to the cell nucleus, where their transcriptional functions can be executed [45]. A second regulatory layer is FOXO acetylation by p300, Cbp (CREB-binding protein) and Pcaf (p300/CBP-associated factors) in response to oxidative stress or DNA binding [46], [47], [48], followed by deacetylation by class I and II histone deacetylases [48], [49], [50], including Sirt1, the NAD^+^-dependent deacetylase encoded by the ortholog of yeast longevity gene Sir2 [51]. We have recently demonstrated that inhibition of PI3K/AKT pathway enhanced FOXO-DNA binding and transcriptional activity [40], [52]. Furthermore, phosphorylation deficient mutant of FOXO enhanced resveratrol-induced FOXO transcriptional activity and apoptosis. Post-translational modification of FOXO proteins is an important mechanism that regulates the ability of different transcription factors to activate distinct gene sets, involved in cell cycle inhibition [53], apoptosis [54], defense against oxidative stress and DNA repair [55]. The enhanced DNA binding activity also serves to limit the availability of FOXO proteins for phosphorylation by AKT [46]. In the present study, we have shown that resveratrol induced apoptosis in prostate cancer cells through activation of FOXO transcription factors. Similarly in another study, we have demonstrated that inhibition of FOXO transcription factors by shRNA blocked resveratrol-induced upregulation of Bim, TRAIL, DR4, DR5, p27^/Kip1^ and apoptosis, and resveratrol-induced inhibition of cyclin D1 in prostate cancer cells *in vitro* [40]. Our data suggest that resveratrol induces cell cycle arrest and apoptosis through regulation of FOXO transcription factors in prostate cancer cells.

The FOXO transcription factors have been shown to be constitutively activated in various human malignancies, including prostate cancer [39], [56]. FOXOs are shown to contribute to development and/or progression of malignancy by regulating the expression of genes involved in cell growth, differentiation, apoptosis, angiogenesis and metastasis [39], [56]. We have recently demonstrated that FOXO transcription factors and VEGF neutralizing antibody enhance anti-angiogenic effects of resveratrol [57]. Prostate cancer cells have been reported to have constitutive FOXO activity due to increased activity of the AKT and ERK kinases. Activation of FOXO may inhibit cell growth, proliferation and angiogenesis, and induce apoptosis by regulating expression of genes such as FasL, Bim, cyclin D1, p27 and TRAIL [39], [40], [56]. Thus, FOXO-mediated expression of genes, involved in angiogenesis, invasion and metastasis may further contribute to the progression of prostate cancer. Constitutive FOXO activity has also been demonstrated in primary prostate cancer tissue samples and suggested to have prognostic importance for a subset of primary tumors. In the present study, resveratrol induced the activation of FKHRL1 and its gene products in PC-3 xenografted tumors. These findings suggest that FOXO may play a role in human prostate cancer development, and/or progression, and resveratrol can inhibit these processes through regulation of FOXO-regulated gene products.

During malignant neoplastic progression the cells undergo genetic and epigenetic cancer specific alterations that finally lead to a loss of tissue homeostasis and restructuring of the microenvironment. The invasion of cancer cells through connective tissue is a crucial prerequisite for metastasis formation. MMPs are up-regulated in many tumors and have been implicated in tumor progression and metastasis. MMPs are critical for pericellular degradation of the extracellular matrix, thereby promoting tumor cell invasion and dissemination. To grow efficiently *in vivo*, tumor cells induce angiogenesis in both primary solid tumors and metastatic foci. In the present study, treatment of xenografted mice with resveratrol plus TRAIL significantly inhibited tumor cell proliferation, metastasis and angiogenesis, and induced apoptosis than single agent alone. Furthermore, resveratrol inhibited the growth of PC-3 xenografts and enhanced the apoptosis-inducing potential of TRAIL probably through regulation of apoptosis, angiogenesis and metastasis.

TRAIL induces apoptosis in cancer cells which express TRAIL-R1/DR4 and TRAIL-R2/DR5. We have shown that the upregulation of death receptors by chemotherapeutic drugs, irradiation and chemopreventive agents enhance or sensitize cancer cells to TRAIL treatment [1], [14], [15], [29], [32], [58], [59], [60], [61], [62], [63]. Specifically, TRAIL-resistant LNCaP cells can be sensitized by chemotherapeutic drugs and irradiation through upregulation of death receptors DR4 and/or DR5 [31], [32]. Similarly, our *in vitro* study has demonstrated the upregulation of DR4 and DR5 in PC-3 and LNCaP cells by resveratrol [14], [15]. Interestingly, resveratrol sensitized TRAIL-resistant LNCaP xenografts by inhibiting tumor cell proliferation and inducing apoptosis which were correlated with induction of death receptors DR4 and DR5. Death receptor (DR4 and/or DR5) regulation has been shown to be under the control of transcription factor NFκB, SP1 and p53 [64], [65], [66]. Inducible silencing of DR5 *in vivo* promoted bioluminescent colon tumor xenograft growth and confers resistance to chemotherapeutic agent 5-fluorouracil [67]. These finding suggest that upregulation of DR4 and DR5 by resveratrol may be one of the mechanisms by which resveratrol enhances the therapeutic potential of TRAIL.

In a recent report, resveratrol and resveratrol-4′-*O*-sulfate were able to inhibit the activity of COX-1 and -2, at concentrations that have been shown to be achievable in human plasma [68]. These data indicate that resveratrol and its 4′-*O*-sulfate metabolite may mediate or contribute to the health benefits attributed previously only to resveratrol. Resveratrol and its 4′-*O*-sulfate metabolite inhibit COX-1 and COX-2 with similar efficacy, and X-ray structural and computational studies indicate these compounds bind in the cyclooxygenase sites of the enzymes. In another study, resveratrol was identified as a potent, mechanistic-based inhibitor of COX-1 (but not COX-2) [69]. In the same study, resveratrol was also found to be an ineffective inhibitor against COX-2. Several studies, which measured the PGE2 production using this immunoassay system, have shown that resveratrol is an inhibitor of both COX-1 and -2 enzymatic activities [70]. Furthermore, it has been demonstrated that resveratrol binds directly to COX-2 and that this binding is absolutely required for the inhibition of cancer cell growth by resveratrol [70]. Therefore, the observation that resveratrol and its 4′-*O*sulfate metabolite inhibit both COX-1 and COX-2 enzymes with nearly the same efficacy is of importance since selective inhibition of either one of the enzymes has shown to lead to serious side effects such as gastric ulcer, heart attack, and stroke. Compounds that target both enzymes equipotently instead might provide beneficial effects without the complications due to single enzyme inhibition.

In summary, our *in vivo* experiments have demonstrated that resveratrol can enhance the therapeutic potential of TRAIL through multiple mechanisms. It induces death receptors (DR4 and DR5) and cell cycle inhibitor p27^/KIP1^, upregulates Bax, inhibits antiapoptotic Bcl-2 protein and markers of cell proliferation (PCNA and Ki67), metastasis (MM2 and MMP7) and angiogenesis (VEGF and VEGF-R2). Furthermore, resveratrol activates FKHRL1 which may result in regulation of Bim, TRAIL, p27, and cyclin D1. FOXO transcription factor has been shown to regulate invasion, metastasis and angiogenesis. All these events will significantly contribute to the antiproliferative and antitumor activities of resveratrol. Our studies demonstrate strong clinical potential because resveratrol, either alone or in combination with TRAIL, can be used for the management of prostate cancer.

## Methods

### Ethics statement

All experiments involving animals were approved by the Institutional Animal Care and Use Committee (IACUC) at the University of Texas Health Science Center at Tyler, protocol #373.

### Reagents

Antibodies against Bcl-2, Bax, TRAIL-R1/DR4, TRAIL-R2/DR5, CD31, VEGF, VEGFR2 and β-actin were purchased from Santa Cruz Biotechnology Inc. (Santa Cruz, CA). Antibodies against p27^/KIP1^, phospho-FKHRL1, cyclin D1, MMP-2 and MMP-9 were purchased from Cell Signaling Technology, Inc. (Danvers, MA). Enhanced chemiluminescence (ECL) Western blot detection reagents were from Amersham Life Sciences Inc. (Arlington Heights, IL). Terminal Deoxynucleotidyl Transferase Biotin-dUTP Nick End Labeling (TUNEL) assay kit was purchased from EMD Biosciences (San Diego, CA). TRAIL was purified as described elsewhere [71]. Resveratrol was purchased from LKT Laboratories, Inc. (St. Paul, MN).

### Western Blot Analysis

Western blots were performed as we described earlier [42]. In brief, tumors were lysed in RIPA buffer containing 1 X protease inhibitor cocktail, and protein concentrations were determined using the Bradford assay (Bio-Rad, Philadelphia, PA). Proteins were separated by 12.5% SDS/PAGE and transferred to Immobilon membranes (Millipore, Bedford, MA) using semidry method. After blotting in 5% nonfat dry milk in TBS, the membranes were incubated with primary antibodies at 1∶1,000 dilution in TBS overnight at 4°C, and then secondary antibodies conjugated with horseradish peroxidase at 1∶5,000 dilution in TBS-Tween 20 for 1 hour at room temperature. Membranes were washed three times with TBS-Tween 20, and protein bands were visualized on X-ray film using an enhanced chemiluminescence system.

### Xenograft Assays in Nude Mice

Athymic nude mice (Balb c nu/nu, 4–6 weeks old) were purchased from the National Cancer Institute (Frederick, MD). PC-3 cells (2×10^6^ cells as a 50% suspension in matrigel, Becton Dickinson, Bedford, MA) in a final volume of 0.1 ml were injected subcutaneously at right flank of Balb c nude mice. When the average tumor volume reached about 100 mm^3^, mice were randomized into four groups of 10 mice/group, and the following treatment protocol was implemented: *Group 1*, vehicle control (0.1 ml normal saline) administered through gavage, three times/week (Monday, Wednesday and Friday) beginning when tumor volume reached about 100 mm^3^; *Group 2*, TRAIL (15 mg/kg) administered through i.v. on day 1, 7, 14, and 21; *Group 3*, resveratrol (30 mg/kg, in 0.1 ml normal saline) administered through gavage, three times/week (Monday, Wednesday and Friday); *Group 4*, resveratrol and TRAIL, resveratrol administered through gavage, and TRAIL administered through i.v. Mice were housed under pathogen-free conditions and maintained on a 12 h light/12 h dark cycle, with food and water supplied *ad libitum*.

### Immunohistochemistry

Immunohistochemistry was performed as described earlier (28, 29). In brief, tumor tissues were collected, excised and fixed with 10% formalin, embedded in paraffin and sectioned. Tissue sections were stained with primary antibodies against Bax, Bcl-2, DR4, DR5, Ki-67, PCNA, p27^/Kip1^, phospho-FKHRL1, CD31, VEGF, VEGFR2, MMP-2 and MMP-9 or TUNEL reaction mixture. For immunohistochemistry, sections were fixed, air-dried, and incubated with various primary antibodies at room temperature for 4 h. Subsequently, slides were washed three times in PBS and incubated with secondary antibody at room temperature for 1 h. Finally, alkaline phosphatase or hydrogen peroxide polymer-AEC chromagen substrate kits were used as per manufacturer's instructions (Lab Vision Corporation). After washing with PBS, Vectashield (Vector Laboratories) mounting medium was applied and sections were coverslipped and imaged.

### Electrophoretic Mobility Shift Assay

Nuclear extracts from tumor samples were incubated with ^32^P-labeled FOXO consensus sequence in a buffer containing 20 mM HEPES (pH 7.9), 20% glycerol, 100 mM KCl, 0.2 mM EDTA, 0.5 mM phenylmethylsulfonyl fluoride, and 0.5 mM DTT for 30 min at 25°C. Protein-DNA complexes were resolved on a high ionic strength 5% polyacrylamide gel containing 0.5 x Tris-borate EDTA buffer [380 mM glycine, 45 mM Tris base (pH 8.5), 45 nM boric acid, and 2 mM EDTA]. Dried gels were subjected to autoradiography.

### Statistical Analysis

The mean and SD were calculated for each experimental group. Differences between groups were analyzed by one or two way ANOVA using PRISM statistical analysis software (GrafPad Software, Inc., San Diego, CA). The non-parametric Mann-Whitney U test was performed to assess the difference of tumor volume between control and treatment groups. Significant differences among groups were calculated at P<0.05.
