# Expression of Concern: Resveratrol Enhances Antitumor Activity of TRAIL in Prostate Cancer Xenografts through Activation of FOXO Transcription Factor

**DOI:** 10.1371/journal.pone.0223138

**Published:** 2019-09-24

**Authors:** The PLOS ONE Editors

After publication of this article [1], several concerns were raised for Figs 3, 4 and 6:

- In Fig 3A, there appears to be overlap between the following image pairs, suggesting that all four of the affected panels may represent data from the same slide:
- DR4 TRAIL panel (row 1, column 3) and DR5 Resveratrol panel (row 2, column 2)
- DR5 Resveratrol panel and DR5 TRAIL panel (row 2, column 3)
- DR4 Resveratrol panel (row 1, column 2) and DR5 TRAIL panel

The authors provide here an updated version of Fig 3A in which they replaced all panels for the DR4 and DR5 immunohistochemistry experiments.

**Fig 3:**
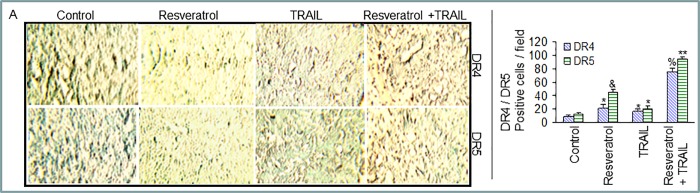
Effects of resveratrol and/or TRAIL on the expression of TRAIL-death receptors. (A), Immunohistochemistry was performed to measure the expressions of TRAIL-R1/DR4 and TRAIL-R2/DR5 in tumor tissues derived from control and treated mice on week 6. Quantification of DR4 and DR5 positive cells are also shown on right panel. (B), Expressions of TRAIL-R1/DR4, TRAIL-R2/DR5 and β-actin in tumor tissues derived on week 6. Western blot analysis was performed to measure the expression of DRs (left panel). Quantification of DR4 and DR5 positive tumor cells (right panel). (C), Measurement of DR4 and DR5 by ELISA. Proteins extracts were prepared and the expressions of DRs were measured as per manufacturer's instructions.

- In Fig 4A, regions of overlap were noted between the Bax/Resveratrol, Bax/TRAIL, and Bax/Resveratrol+TRAIL immunohistochemistry panels (row 1, columns 1–3) suggesting that these images were derived from the same original slide. The authors noted that this resulted from errors in Fig preparation, and provide here an updated version of Fig 4A in which they replaced all panels for the Bax immunohistochemistry experiments.

**Fig 4:**
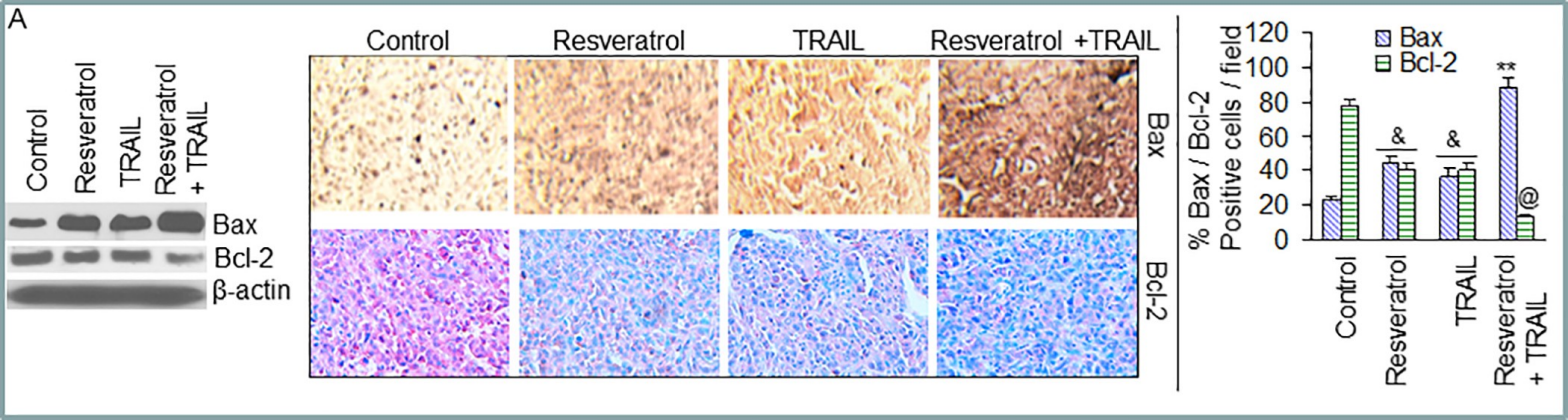
Effects of resveratrol and/or TRAIL on Bcl-2 family members and cell cycle regulatory proteins. (A), Western blot analysis was performed to measure the expressions of Bax and Bcl-2 in tumor tissues (pooled samples) derived from control, resveratrol and/or TRAIL treated mice on week 6 (left panel). β-actin was used as a loading control. Immunohistochemistry was performed to measure the expressions of Bax and Bcl-2 in tumor tissues derived from control and drug-treated mice on week 6 (middle panel). Quantification of Bax and Bcl-2 positive cells in tumor cells (right panel). Data represent mean ± SD, N = 10. &, **, or @ = significantly different from respective controls, P < 0.05. (B), Western blot analysis was performed to measure the expressions of p27^/KIP1^ and cyclin D1 in tumor tissues (pooled samples) derived from control and drug-treated mice on week 6 (left panel). β-actin was used as a loading control. Immunohistochemistry was performed to measure the expressions of p27^/KIP1^ and cyclin D1 in tumor tissues derived from control and drug-treated mice on week 6 (middle panel). Quantification of p27^/KIP1^ and cyclin D1 positive cells in tumor tissues (right panel). Data represent mean ± SD, N = 10. &, %, **, or @ = significantly different from respective controls, P < 0.05.

- There appears to be a region of overlap between the Fig 5A MMP9 Resveratrol panel (row 2, column 2) and the Fig 6B VEGF Control + TRAIL panel (row 2, column 1), when flipped horizontally. The authors noted that data from the MMP9 Resveratrol experiment (Fig 5A) were included in Fig 6B in error, and they provide here an updated version of Fig 6 in which the Control + TRAIL panel has been replaced.

**Fig 5:**
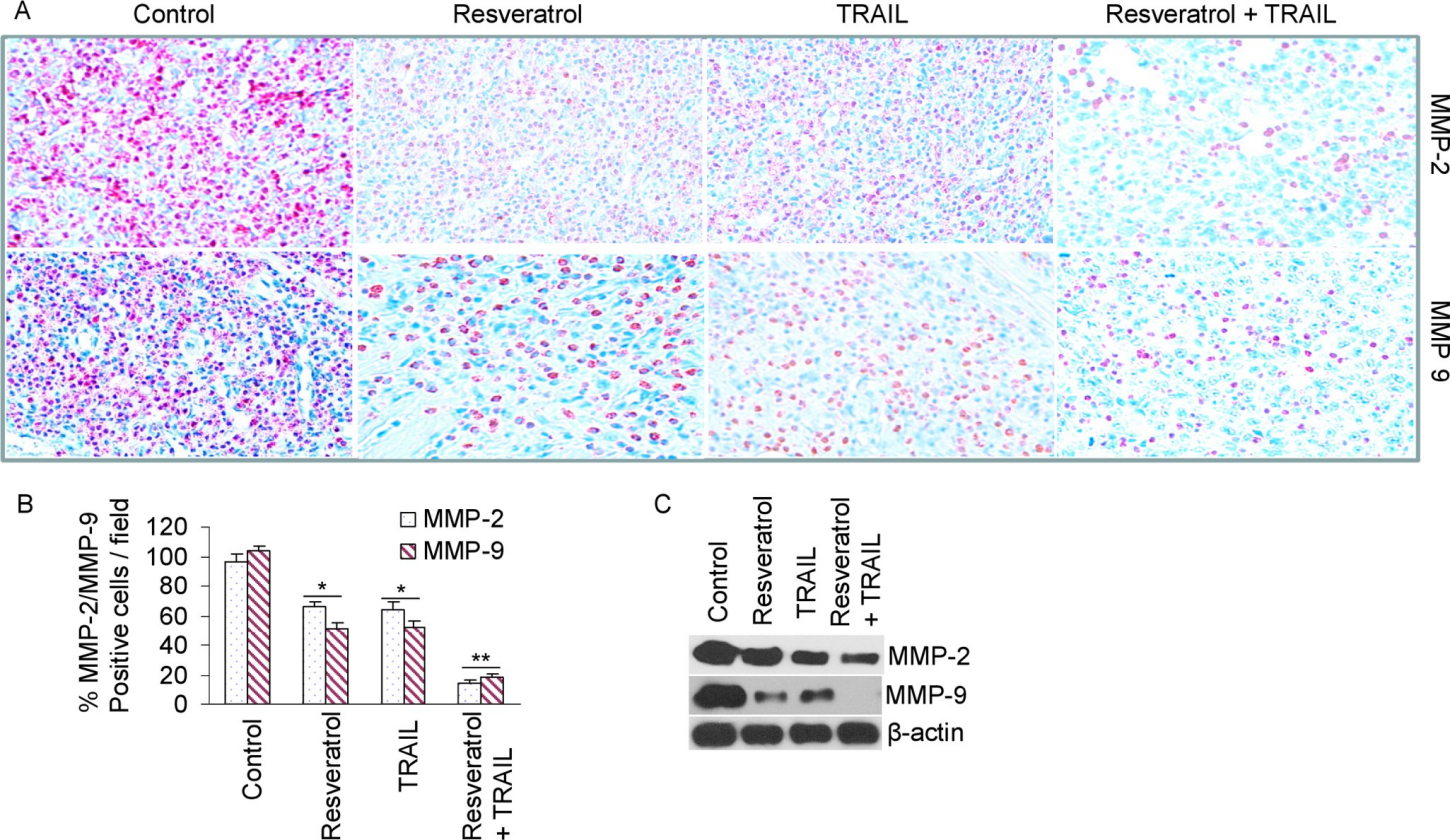
Effects of resveratrol and/or TRAIL on markers of metastasis. (A), Immunohistochemistry was performed to measure the expressions of MMP-2 and MMP-9 in tumor tissues derived from control, resveratrol- and/or TRAIL-treated mice on week 6. (B), Quantification of MMP-2 and MMP-9 positive cells in tumor tissues. Data represent mean ± SD, N = 7. *, or ** = significantly different from respective controls, P < 0.05. (C), Expressions of MMP-2, MMP-9 and β-actin in tumor tissues (pooled samples) were measured on week 6 by the Western blot analysis. β-actin was used as a loading control.

**Fig 6:**
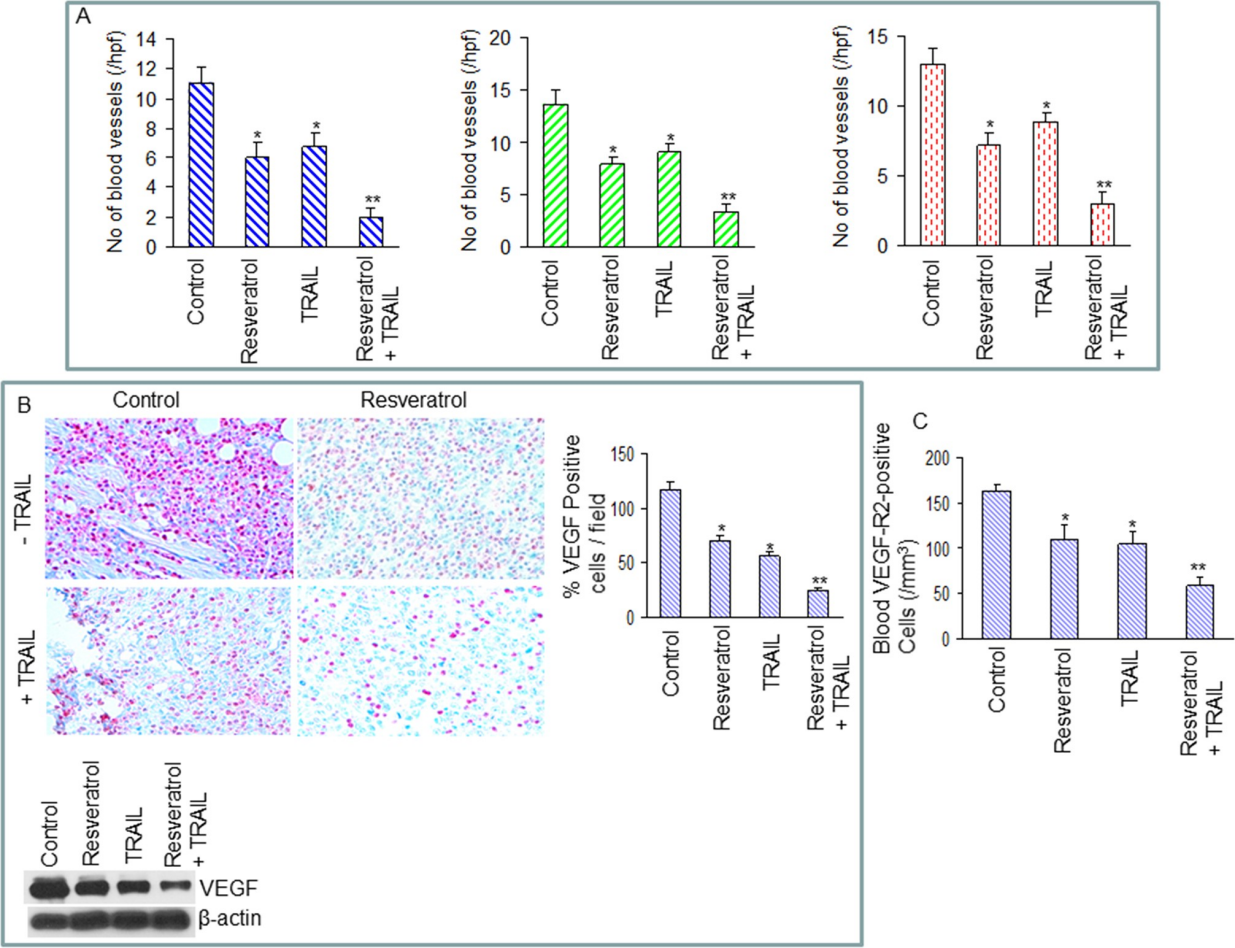
Effects of resveratrol and/or TRAIL on markers of angiogenesis. (A), Left panel, tumor tissue sections derived from control, resveratrol- and/or TRAIL-treated mice on week 6 were stained with H & E and the numbers of blood vessels were counted at 400 X magnification. Each column represents the mean ± SD, N = 7. * or ** = significantly different from control, P < 0.05. Middle panel, blood vessel quantification in tumors derived on week 6. Tumor sections from control and drug-treated mice were stained with anti-CD31 antibody, and the numbers of CD31-positive blood vessels were counted. The results are shown as the mean ± SD, N = 7. * or ** = significantly different from control, P < 0.05. Right panel, tumor sections from control and drug-treated mice obtained on week 6 were stained with anti-von Willebrand Factor (vWF) antibody to evaluate blood vessels. The results are shown as the mean ± SD, N = 7. * or ** = significantly different from control, P < 0.05. (B), Left panel, immunohistochemistry was performed to measure the expression of VEGF in tumor tissues derived from control and drug-treated mice on week 6. Right panel, quantification of VEGF positive cells in tumor tissues. The results are shown as the mean ± SD, N = 7. * or ** = significantly different from control, P < 0.05. Bottom panel, expressions of VEGF in tumor tissues (pooled samples) derived on week 6 were measured by the Western blot analysis. β-actin was used as a loading control. (C), VEGF receptor 2 (VEGF-R2)-positive circulating endothelial cells in mice on week 6. The blood cells from peripheral blood attached to the slide were stained with anti-VEGF-R2 antibody, and the number of VEGF-R2 positive cells was counted under a microscope. The results are shown as the mean ± SD, N = 7. * or ** = significantly different from control, P < 0.05.

The authors noted that the quantitative data shown in the published Figures [1] were obtained from direct microscopy and are unaffected by these Figure errors. However, the original image data used in the quantification have not been provided to verify this claim.

The authors apologize for these errors and provide here the underlying image files supporting results in Figs 3–6 as Supporting Information (S1–S5 Files). Updated Figures and their accompanying legends for the full Figs 3–6 are also provided with this notice; these indicate the number of replicates represented in the published bar graphs, with statistical significance. A member of *PLOS ONE*’s Editorial Board reviewed the updated figure panels and confirmed that the overall conclusions of the study are supported by the new data.

The authors confirm that the underlying data supporting other figures in the published article [1] are available upon request.

The *PLOS ONE* Editors issue this Expression of Concern to notify readers of the issues with the published figures and to provide the additional supporting data for the affected results.

## Supporting information

S1 File Quantitative and western blot data available to support the published version of Fig 3. (ZIP)

S2 File Image and quantitative data underlying the published version of Fig 4. (ZIP)

S3 File Image files supporting replacement Bax data in the revised version of Fig 4A. (ZIP)

S4 File Underlying data for Fig 5. (ZIP)

S5 File Underlying data for updated version of Fig 6. (ZIP)
