# Supplementary figures and images for: Expression of Concern: Resveratrol Enhances Antitumor Activity of TRAIL in Prostate Cancer Xenografts through Activation of FOXO Transcription Factor
Source: PLoS One. 2019 Sep 24;14(9):e0223138. doi: 10.1371/journal.pone.0223138 (PMC6759166; doi:10.1371/journal.pone.0223138)

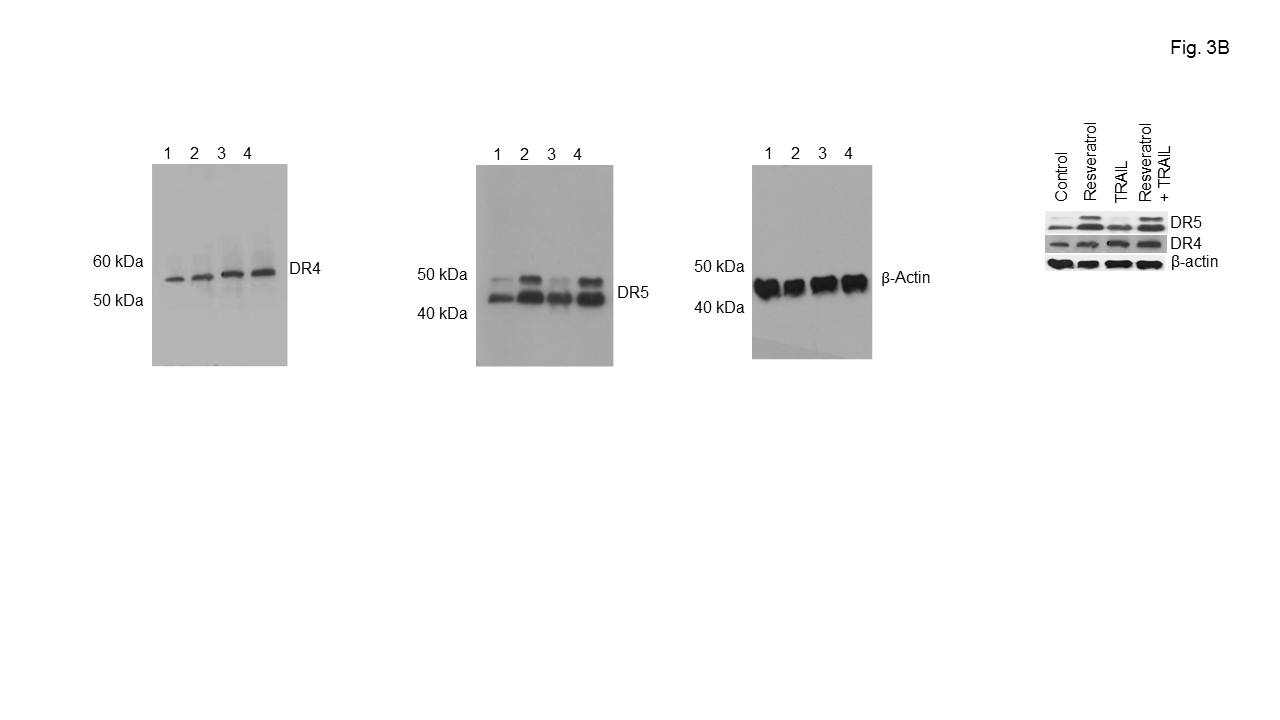

Supplement: S1 File — (ZIP) [file pone.0223138.s001.zip › S1_File/S10_File - new WB Fig 3B.TIF]

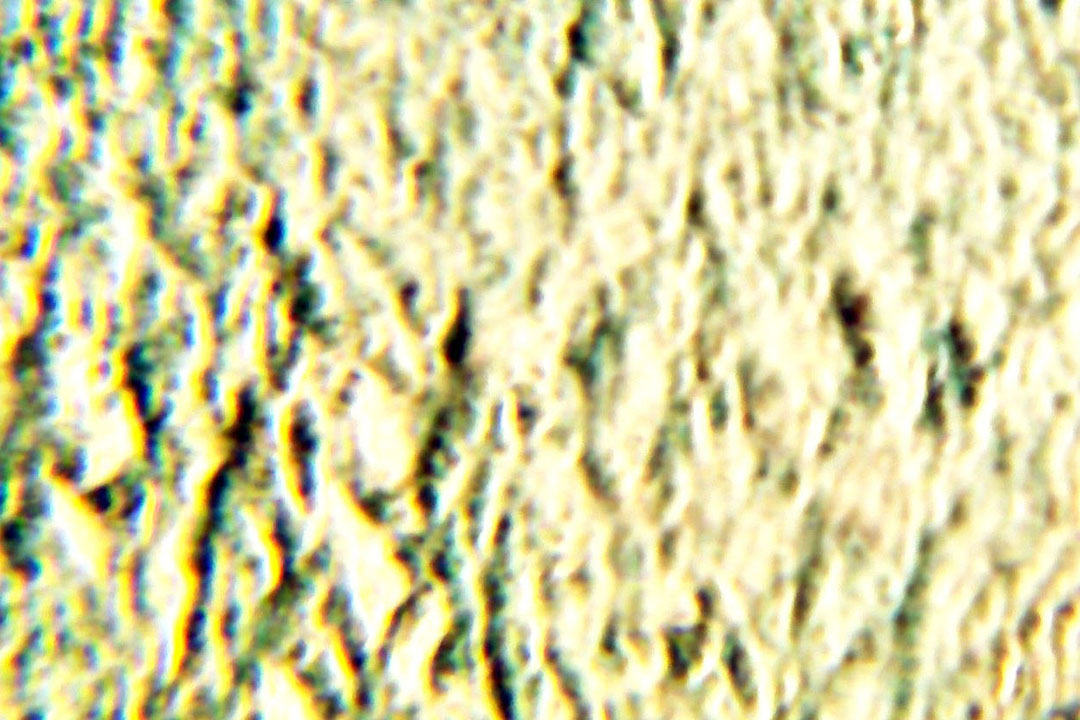

Supplement: S1 File — (ZIP) [file pone.0223138.s001.zip › S1_File/S1_File - new DR4 Control.tif]

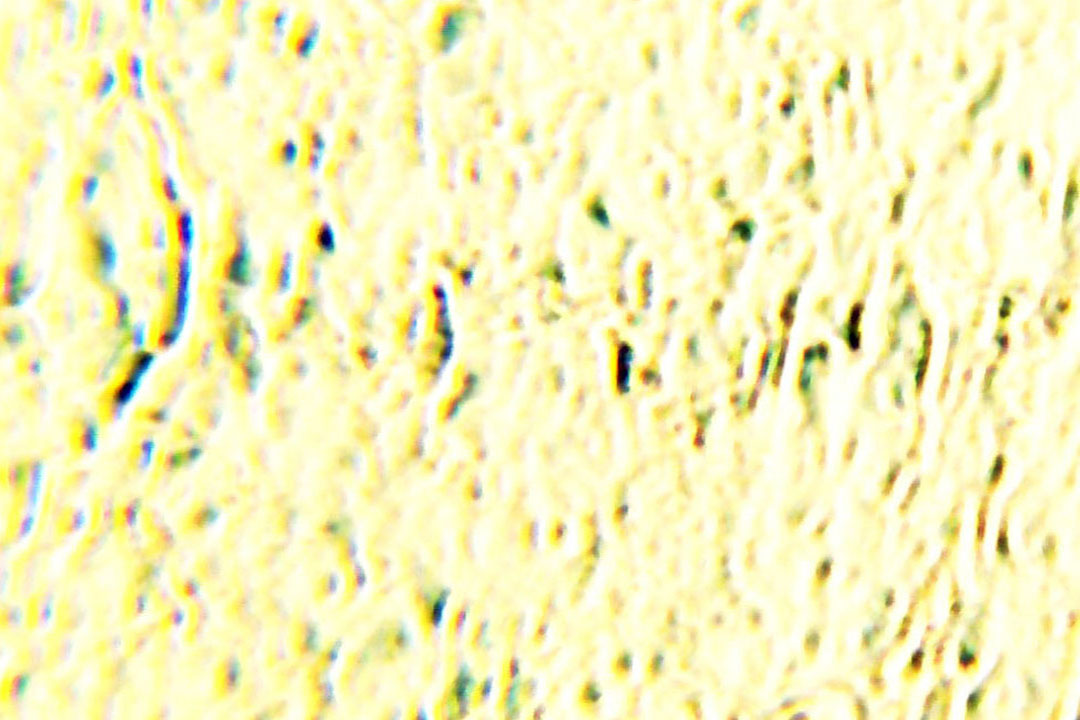

Supplement: S1 File — (ZIP) [file pone.0223138.s001.zip › S1_File/S2_File - new DR4 Resveratrol.tif]

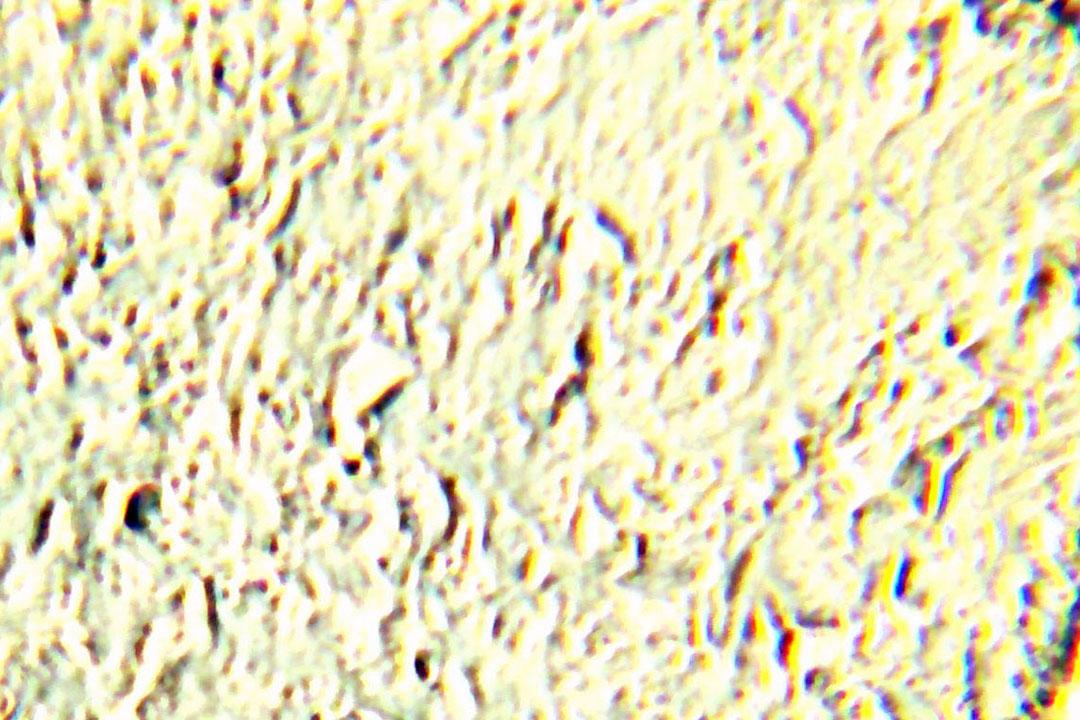

Supplement: S1 File — (ZIP) [file pone.0223138.s001.zip › S1_File/S3_File - new DR4 TRAIL.tif]

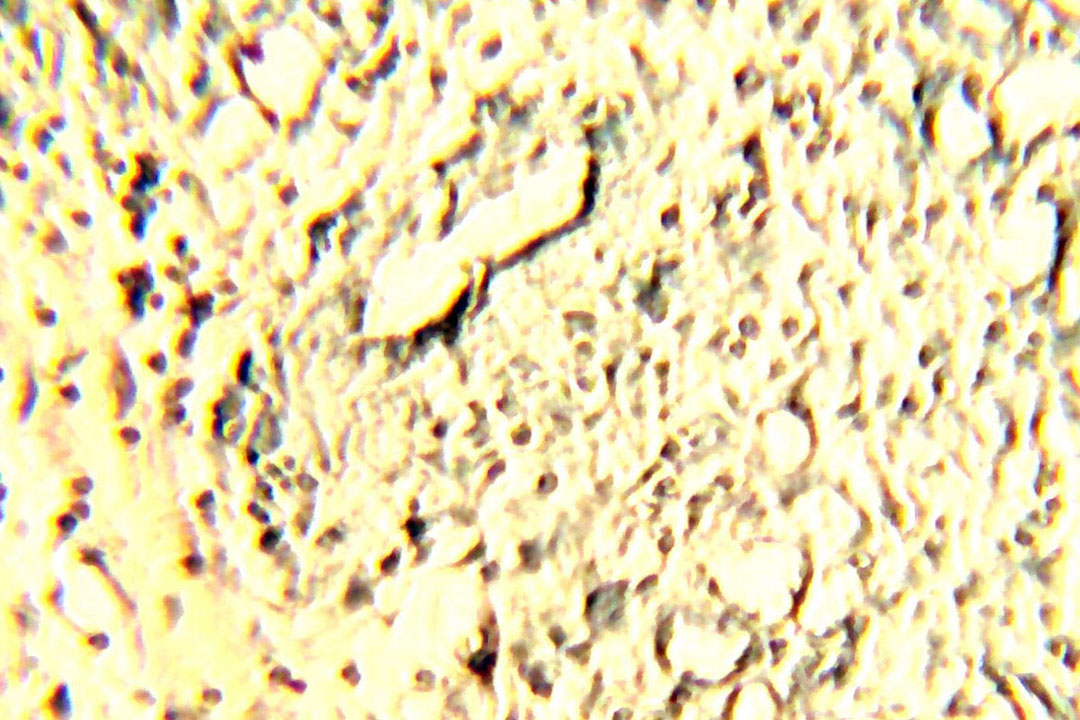

Supplement: S1 File — (ZIP) [file pone.0223138.s001.zip › S1_File/S4_File - new DR4 Resveratrol and TRAIL.tif]

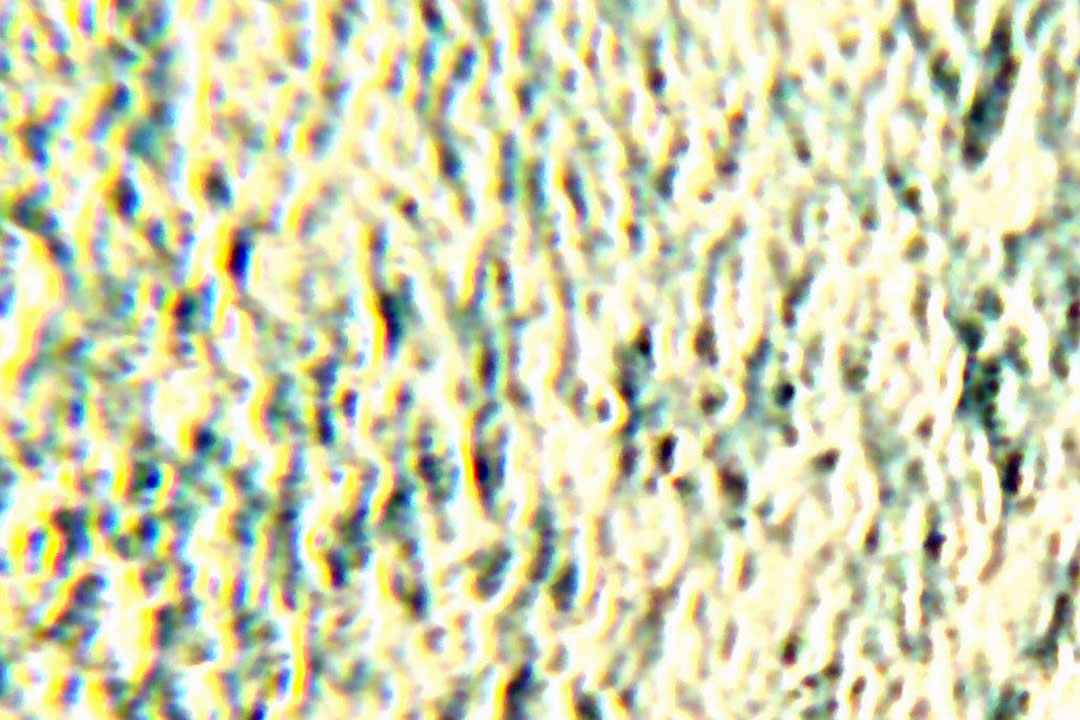

Supplement: S1 File — (ZIP) [file pone.0223138.s001.zip › S1_File/S5_File - new DR5 Control.tif]

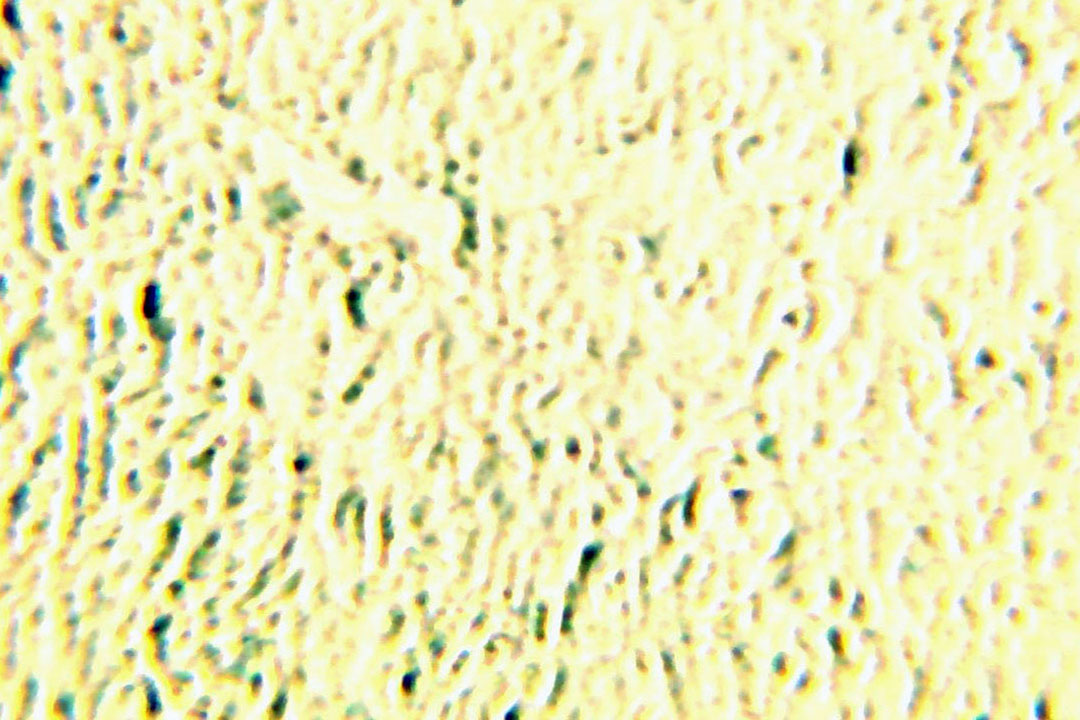

Supplement: S1 File — (ZIP) [file pone.0223138.s001.zip › S1_File/S6_File - new DR5 Resveratrol.tif]

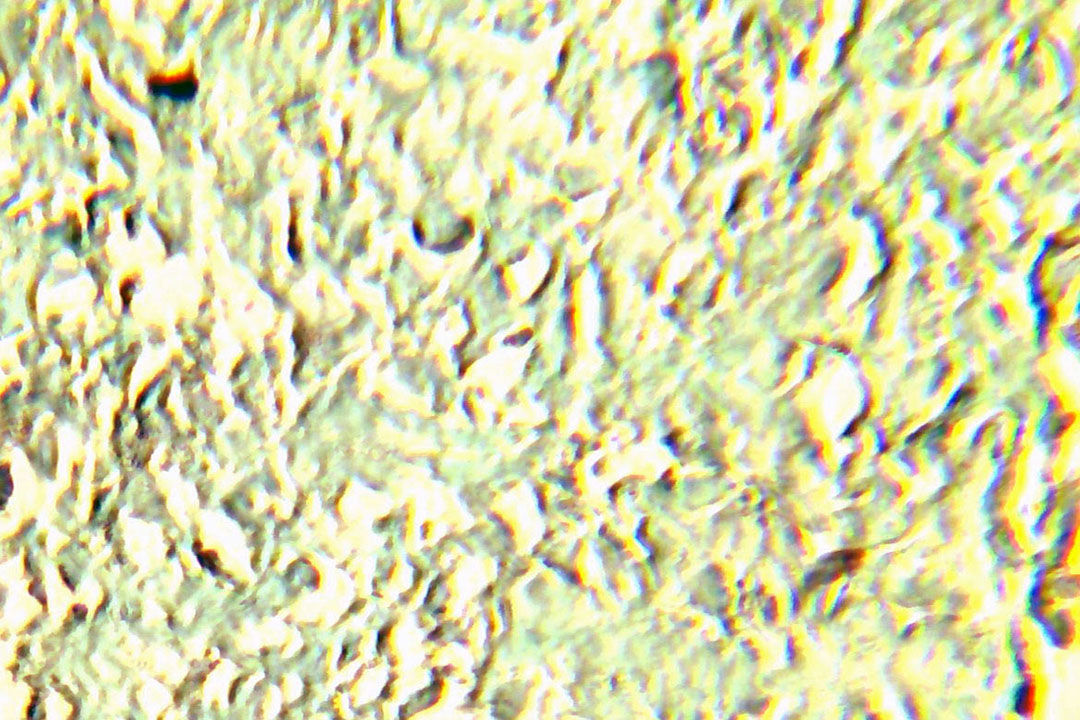

Supplement: S1 File — (ZIP) [file pone.0223138.s001.zip › S1_File/S7_File - new DR5 TRAIL.tif]

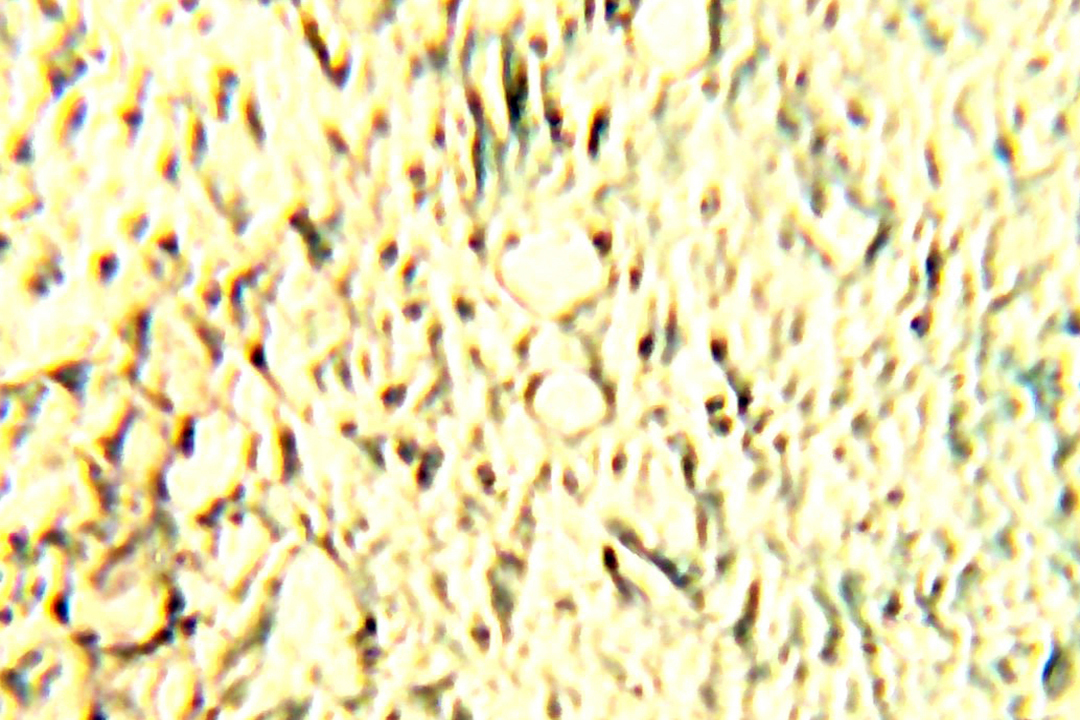

Supplement: S1 File — (ZIP) [file pone.0223138.s001.zip › S1_File/S8_File - new DR5 Resveratrol and TRAIL.tif]

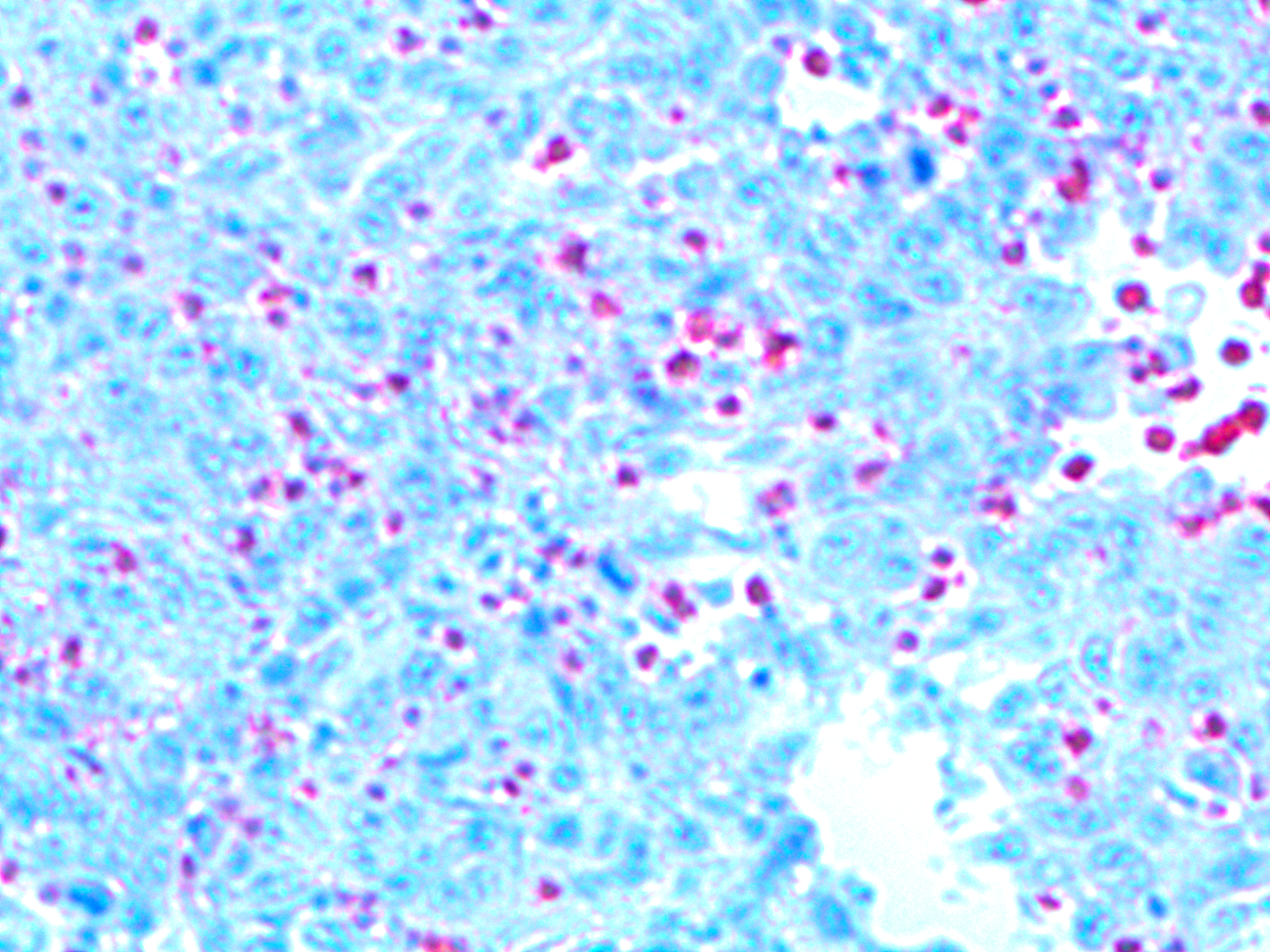

Supplement: S2 File — (ZIP) [file pone.0223138.s002.zip › S2_File/File S10 - 4B published_p27 control.tif]

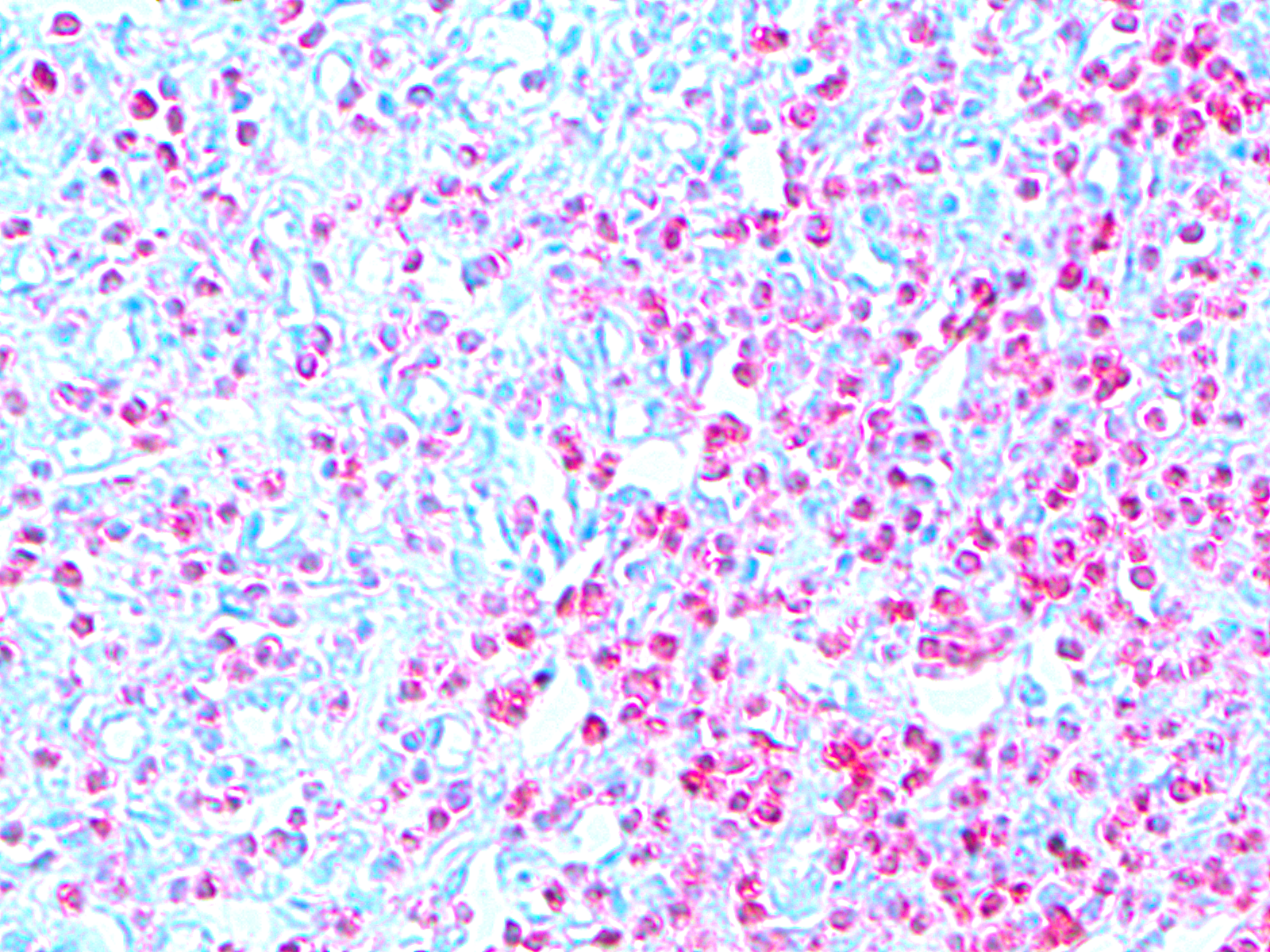

Supplement: S2 File — (ZIP) [file pone.0223138.s002.zip › S2_File/File S11 - 4B published_p27 Resveratrol.tif]

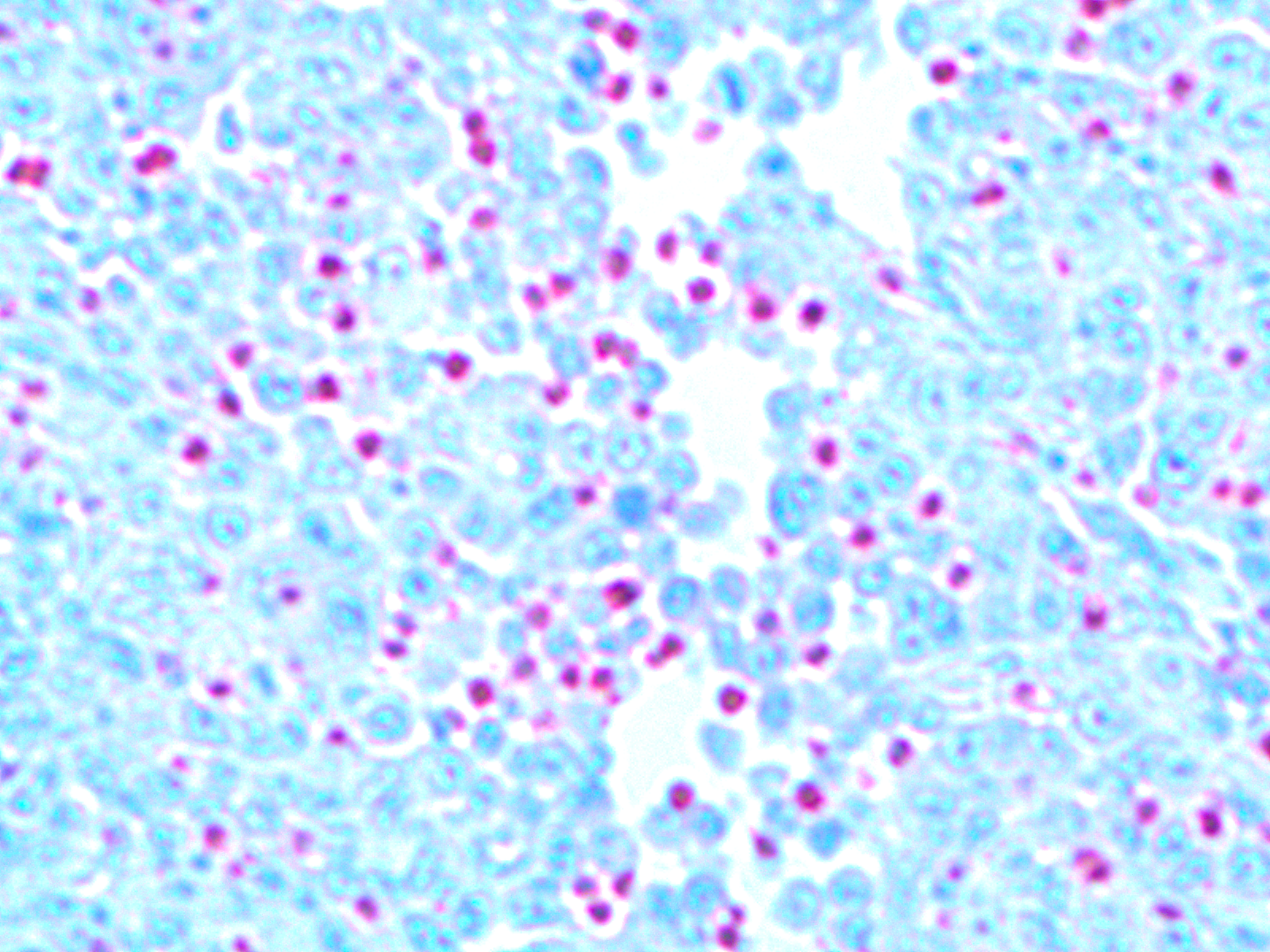

Supplement: S2 File — (ZIP) [file pone.0223138.s002.zip › S2_File/File S12 - 4B published_p27 TRAIL.tif]

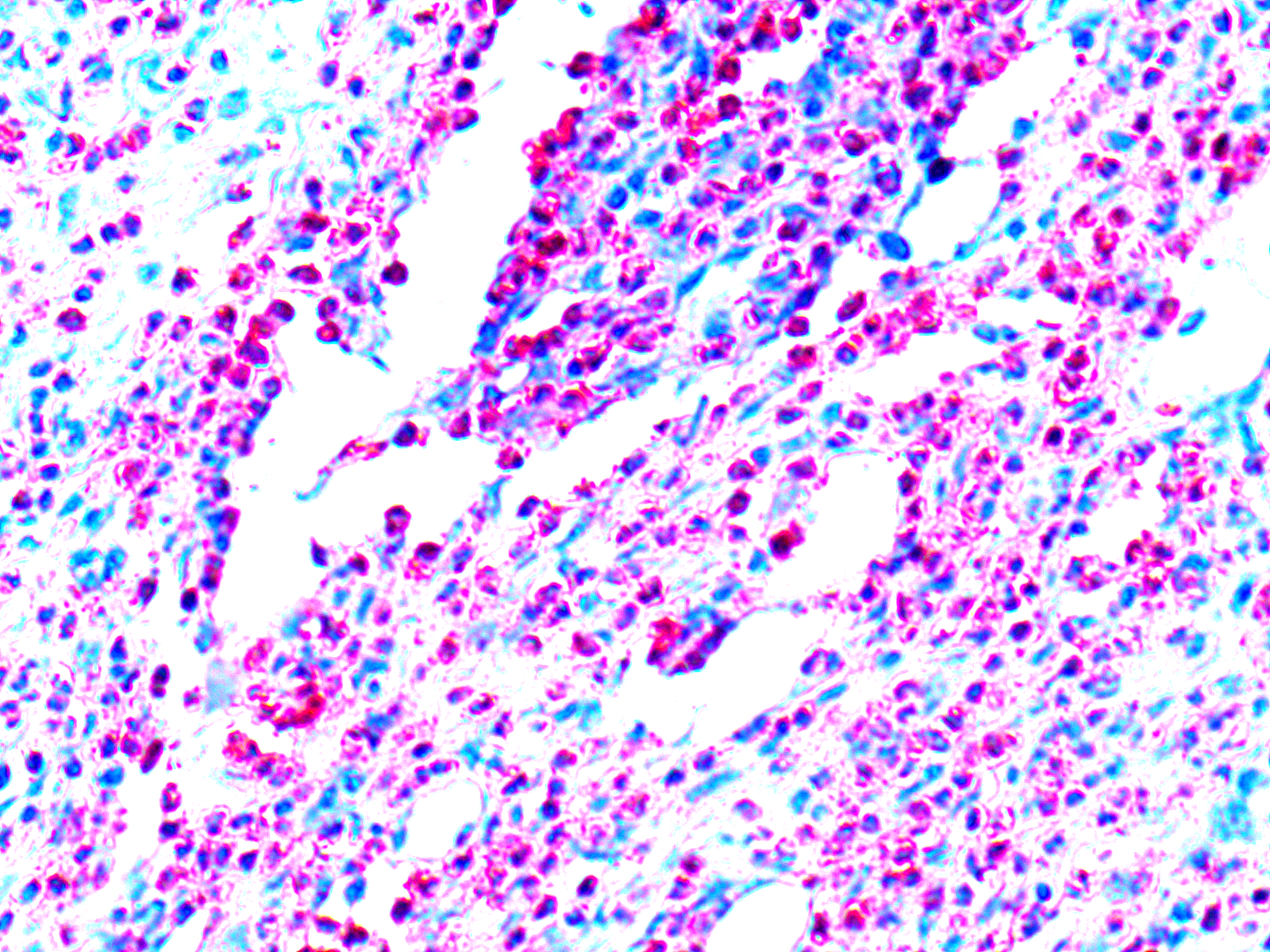

Supplement: S2 File — (ZIP) [file pone.0223138.s002.zip › S2_File/File S13 - 4B published_p27 Resv + TRAIL.tif]

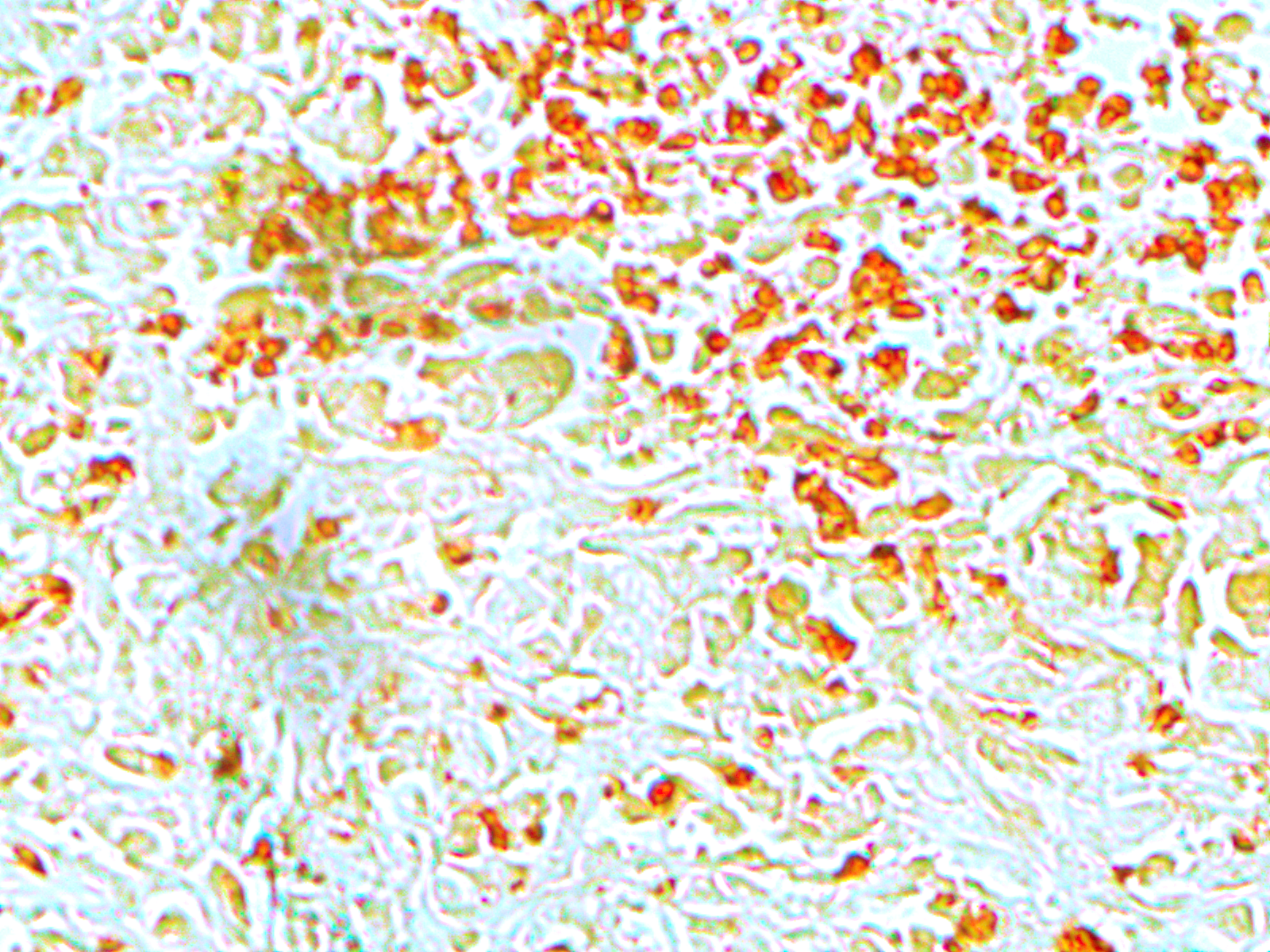

Supplement: S2 File — (ZIP) [file pone.0223138.s002.zip › S2_File/File S14 - 4B published_CycD1 Control.tif]

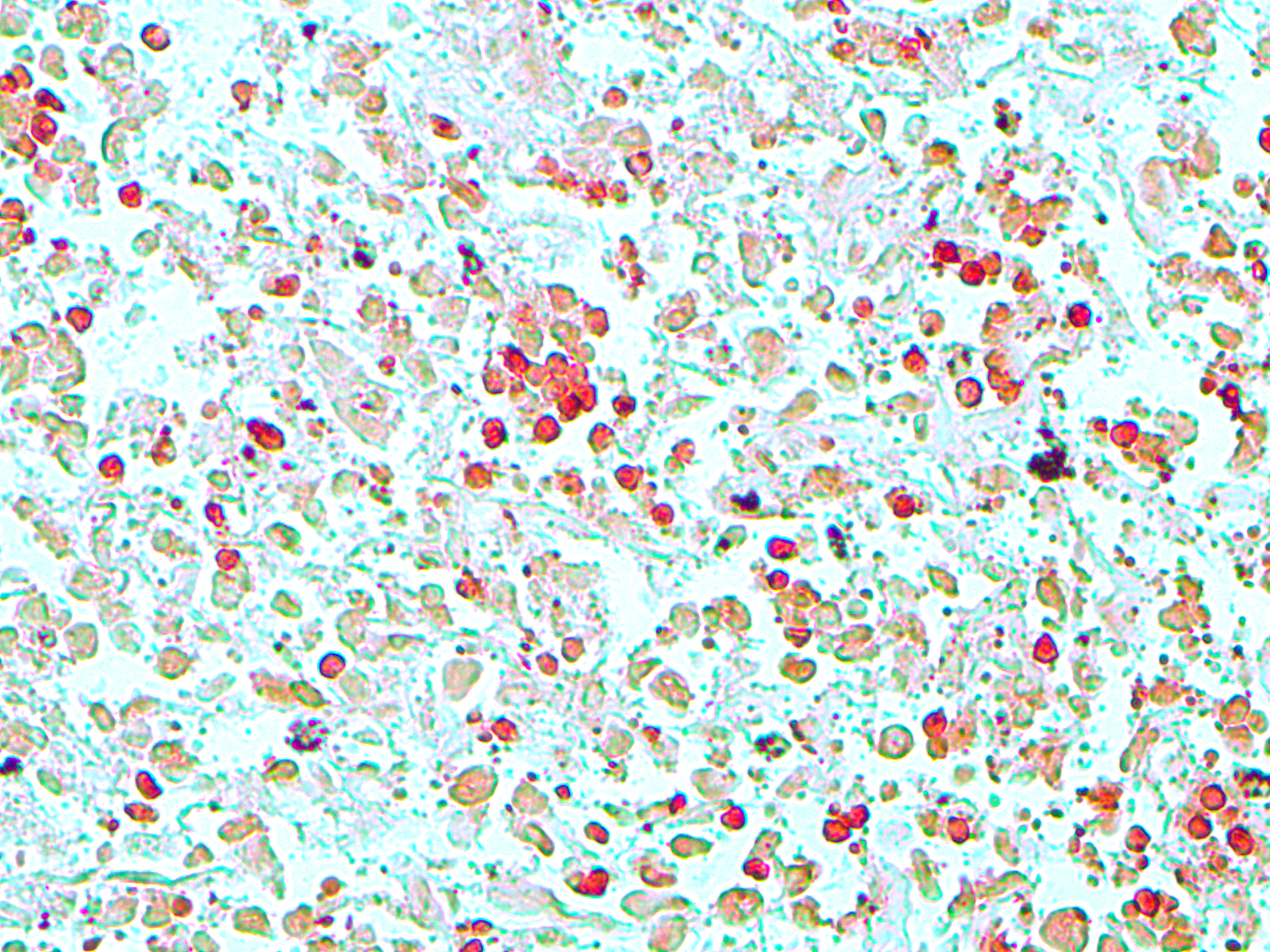

Supplement: S2 File — (ZIP) [file pone.0223138.s002.zip › S2_File/File S15 - 4B published_CycD1 Resveratrol.tif]

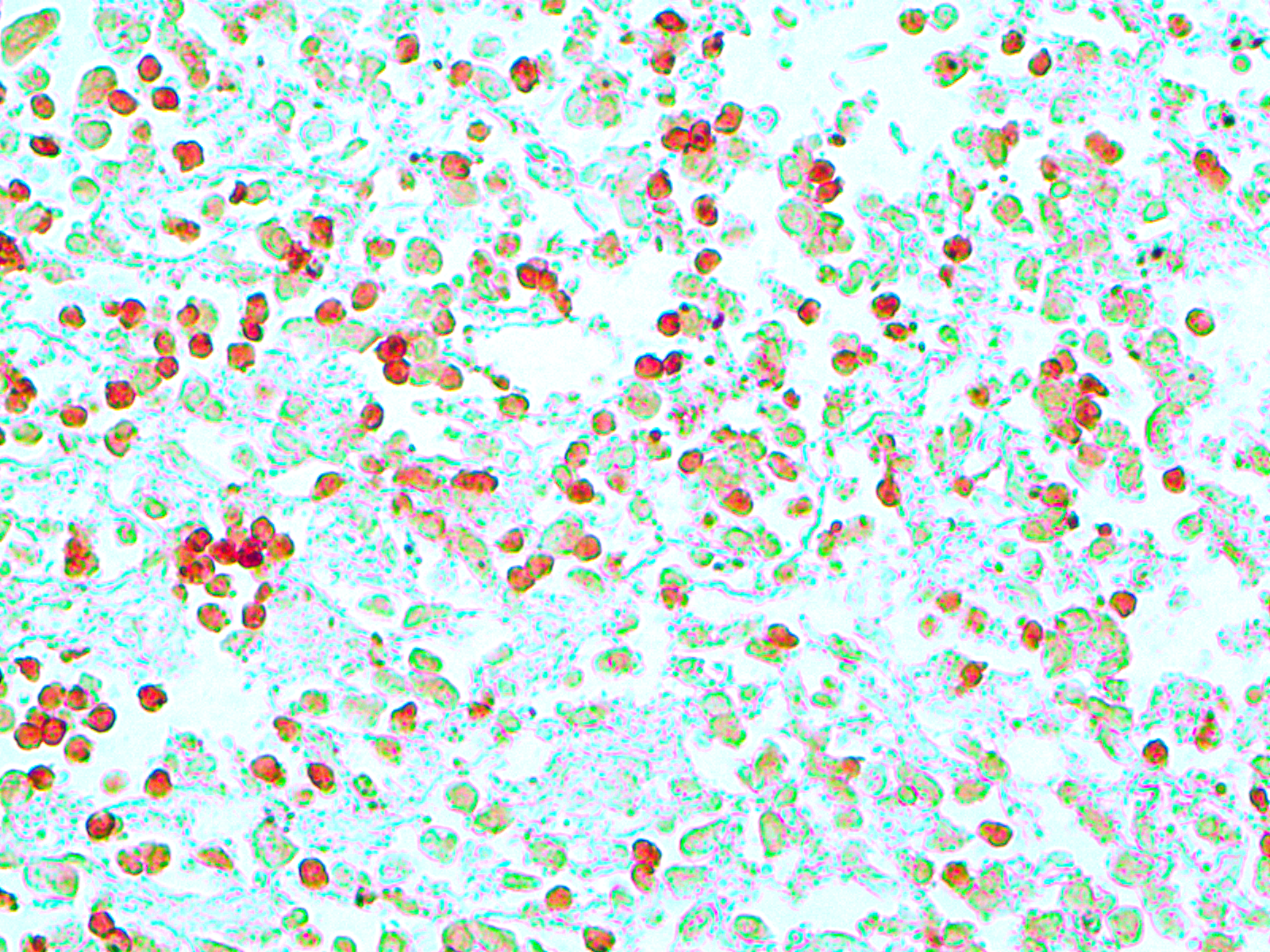

Supplement: S2 File — (ZIP) [file pone.0223138.s002.zip › S2_File/File S16 - 4B published_CycD1 TRAIL.tif]

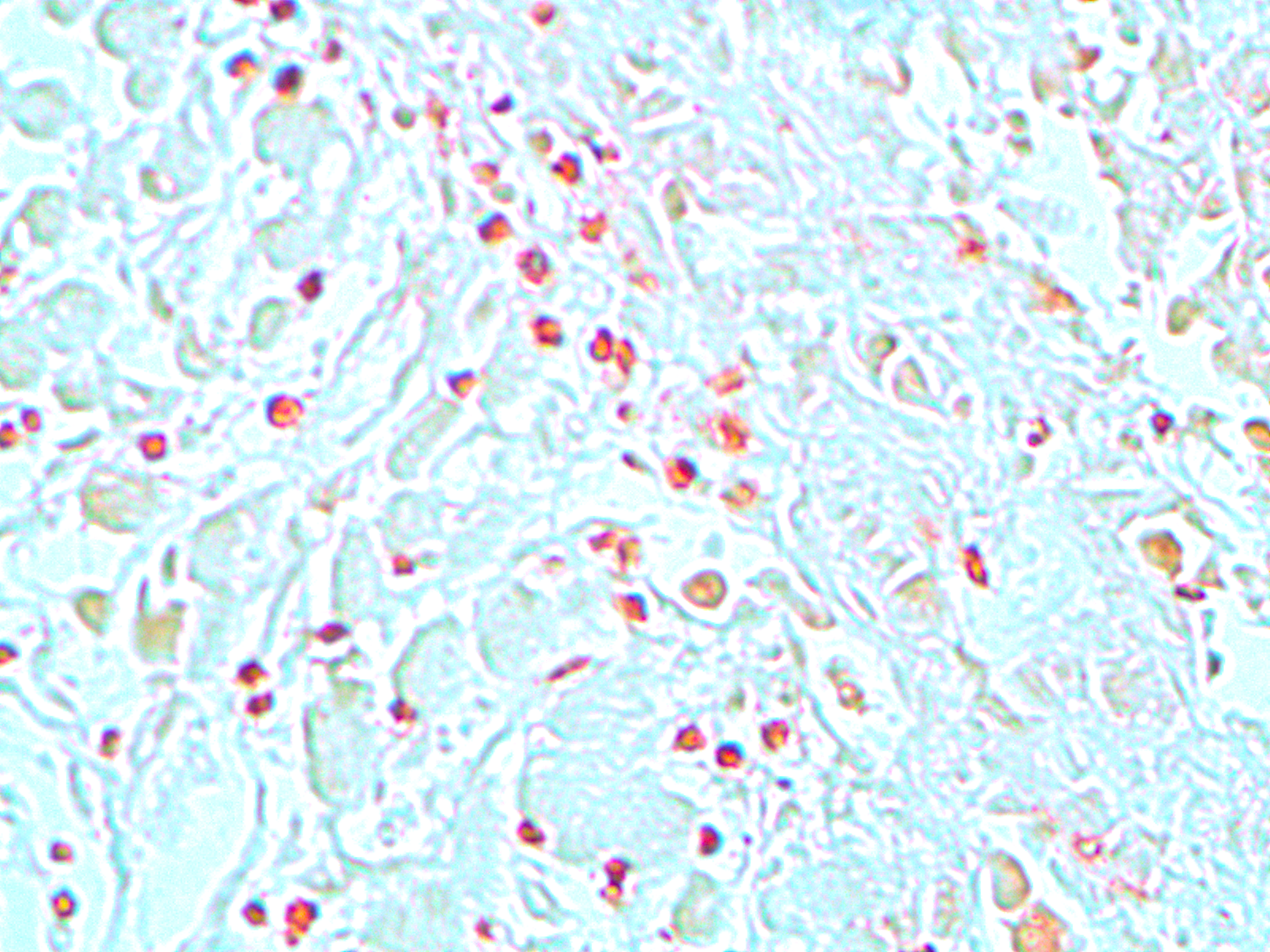

Supplement: S2 File — (ZIP) [file pone.0223138.s002.zip › S2_File/File S17 - 4B published_CycD1 Resv + TRAIL.tif]

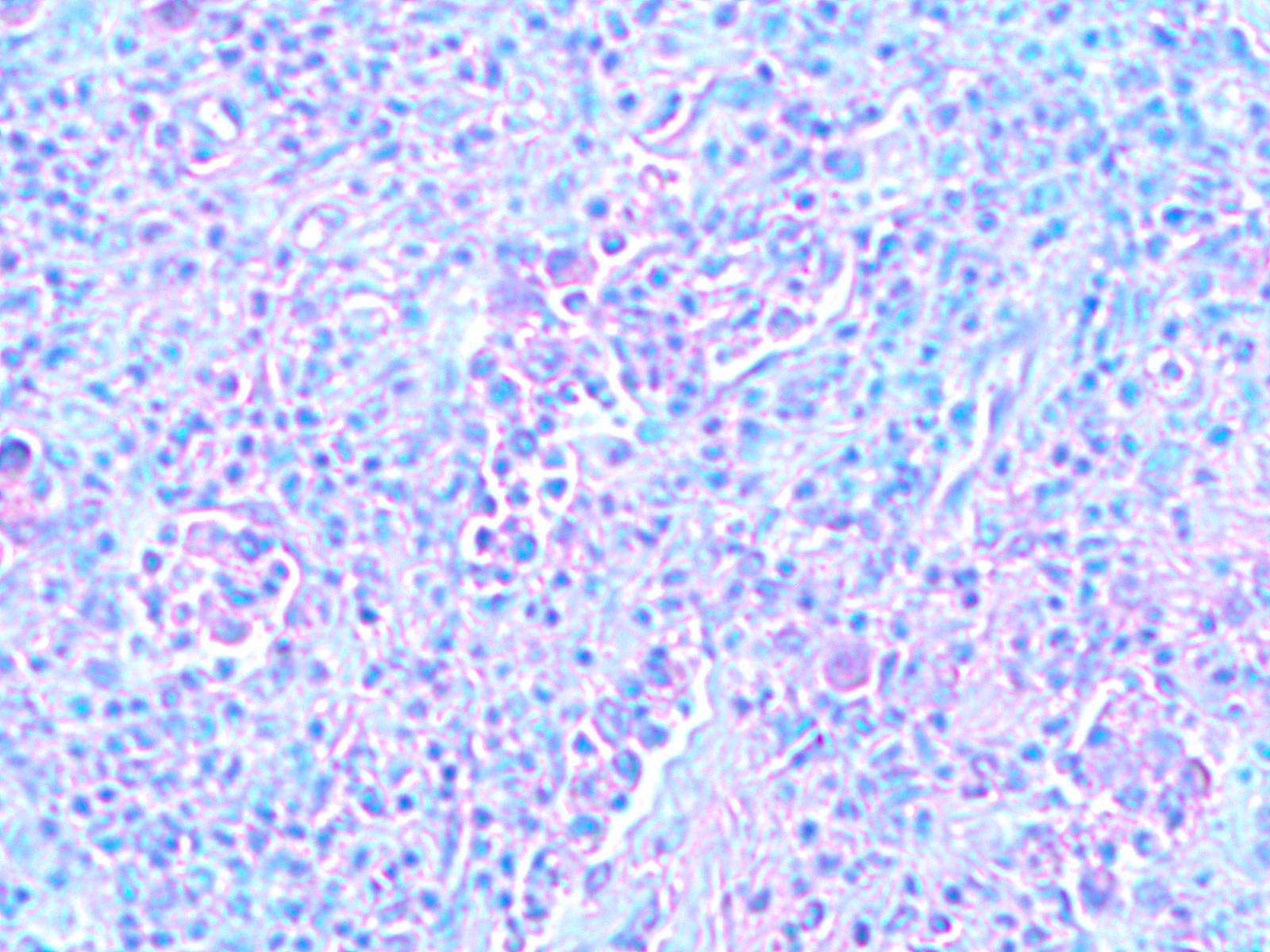

Supplement: S2 File — (ZIP) [file pone.0223138.s002.zip › S2_File/File S2 - 4A published_Bax control.tif]

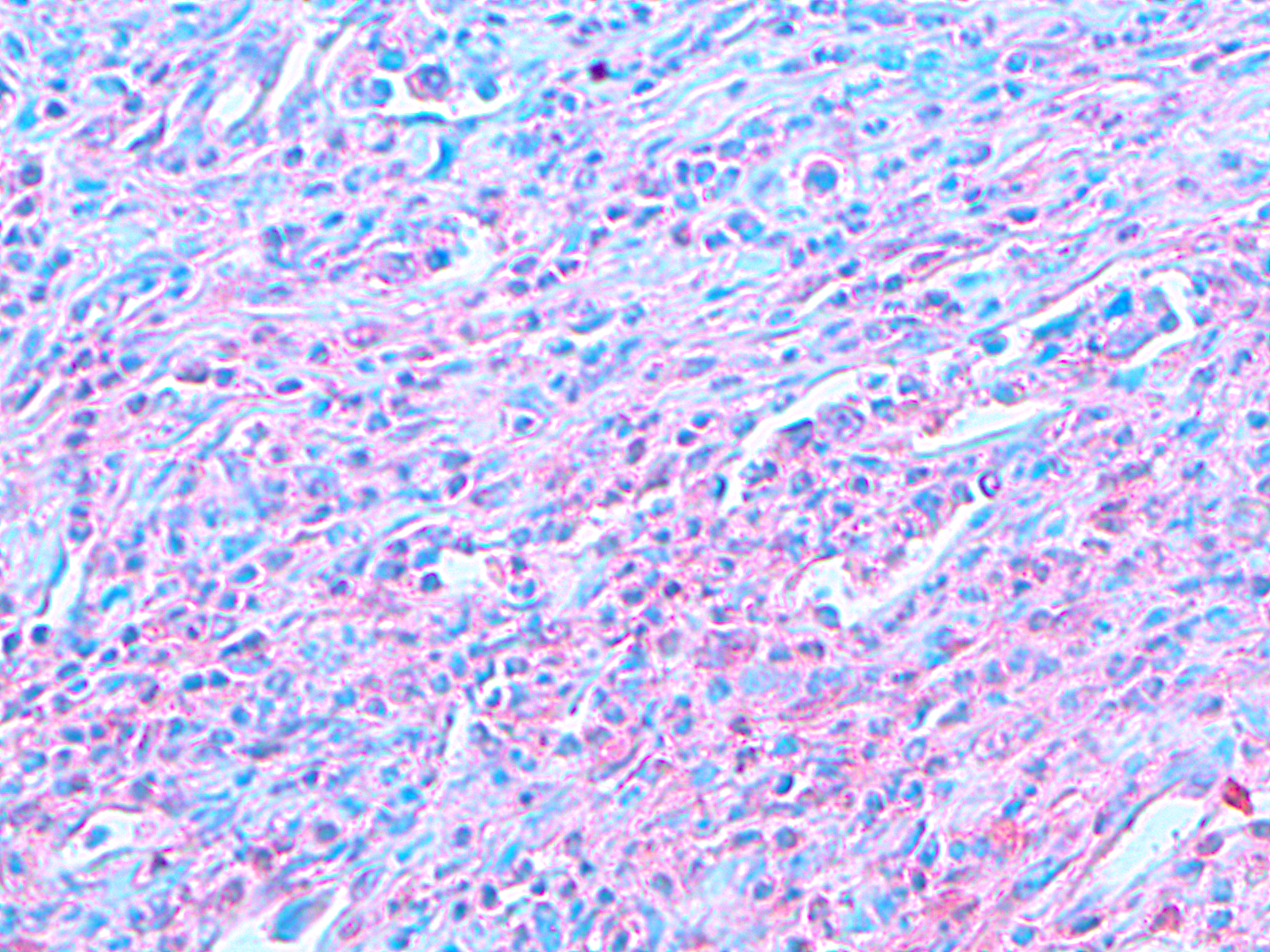

Supplement: S2 File — (ZIP) [file pone.0223138.s002.zip › S2_File/File S3 - 4A published_Bax Resveratrol.tif]

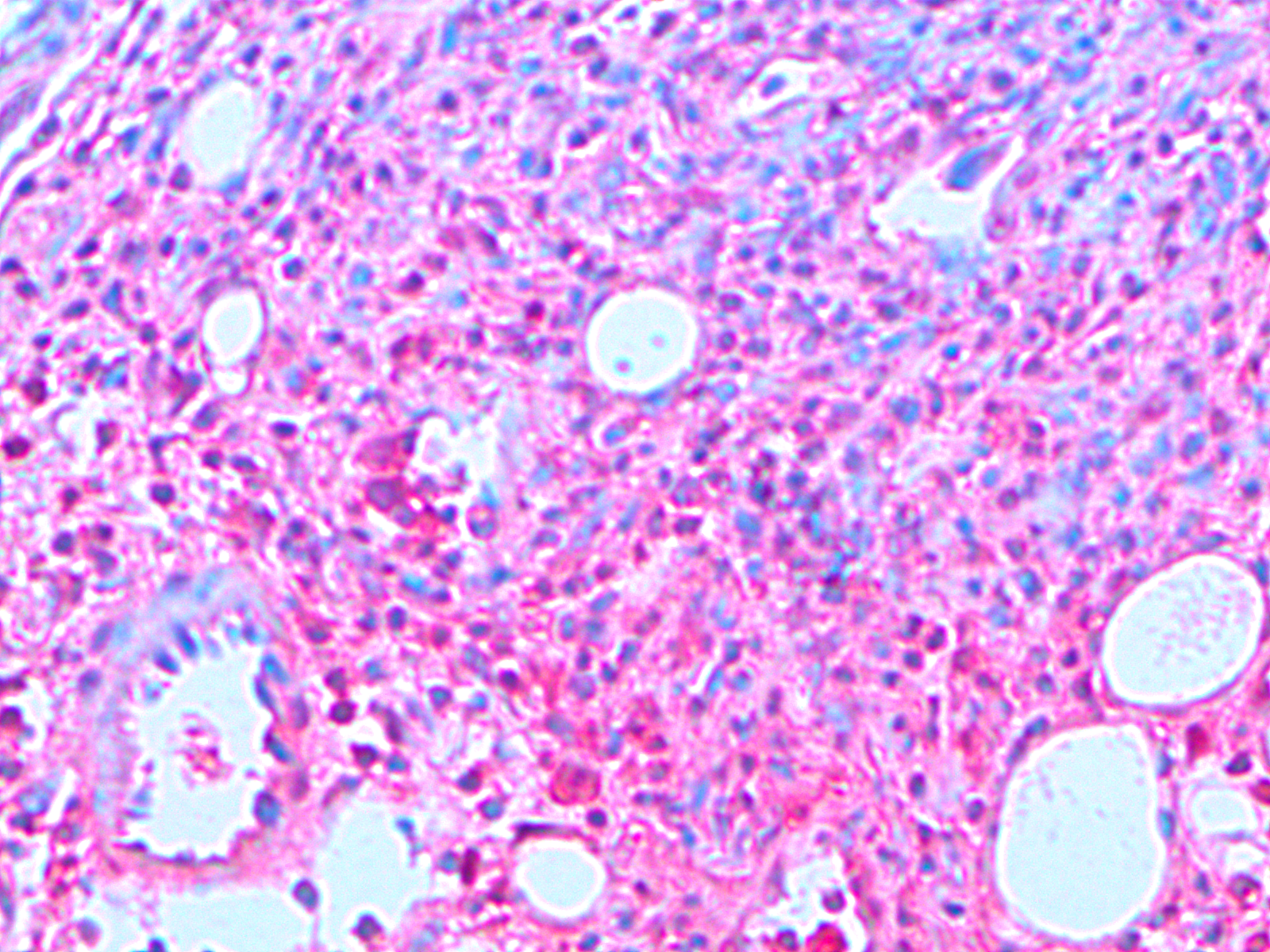

Supplement: S2 File — (ZIP) [file pone.0223138.s002.zip › S2_File/File S4 - 4A published_Bax Resv + TRAIL.tif]

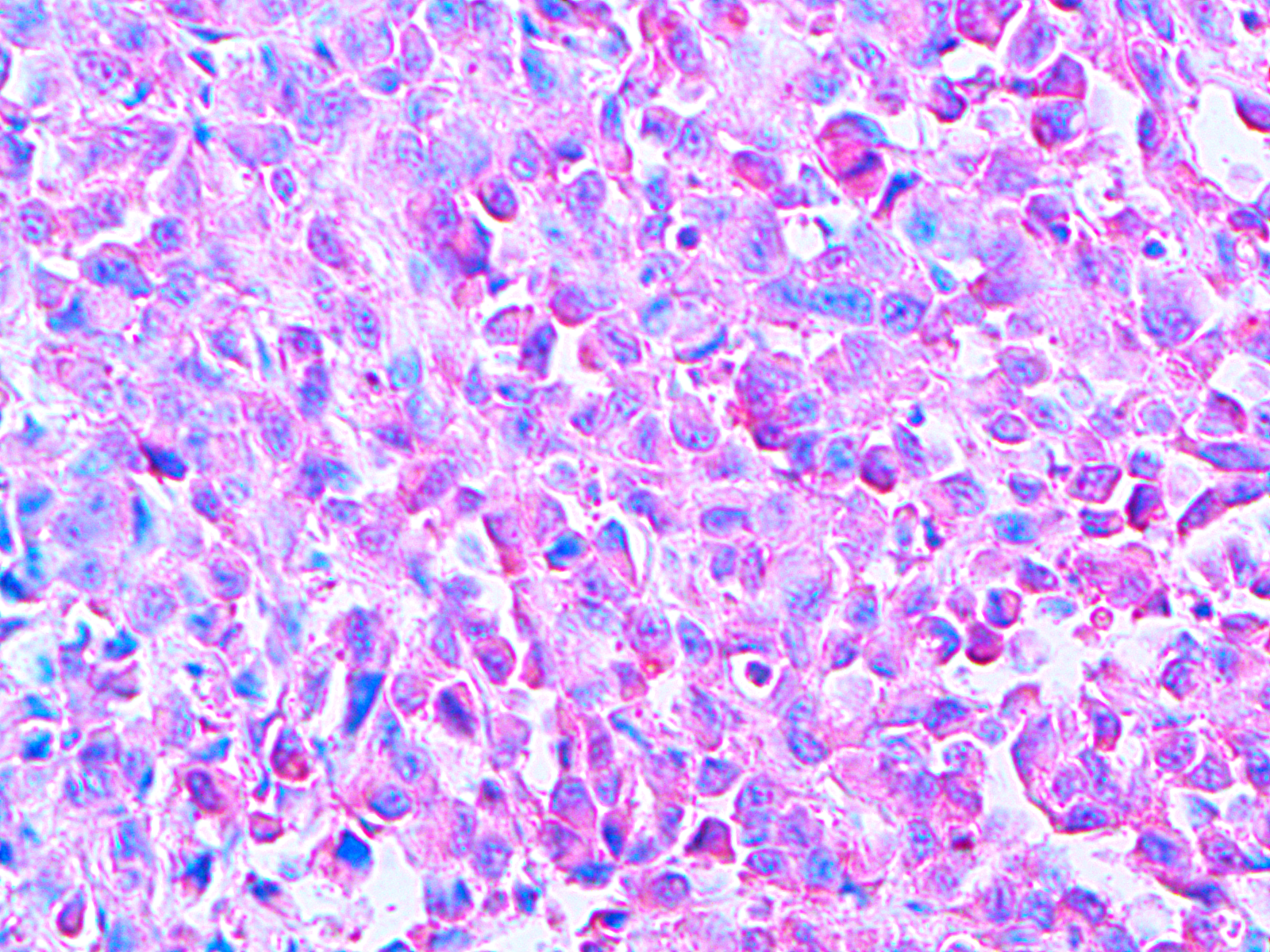

Supplement: S2 File — (ZIP) [file pone.0223138.s002.zip › S2_File/File S6 - 4A published_Bcl2 control.tif]

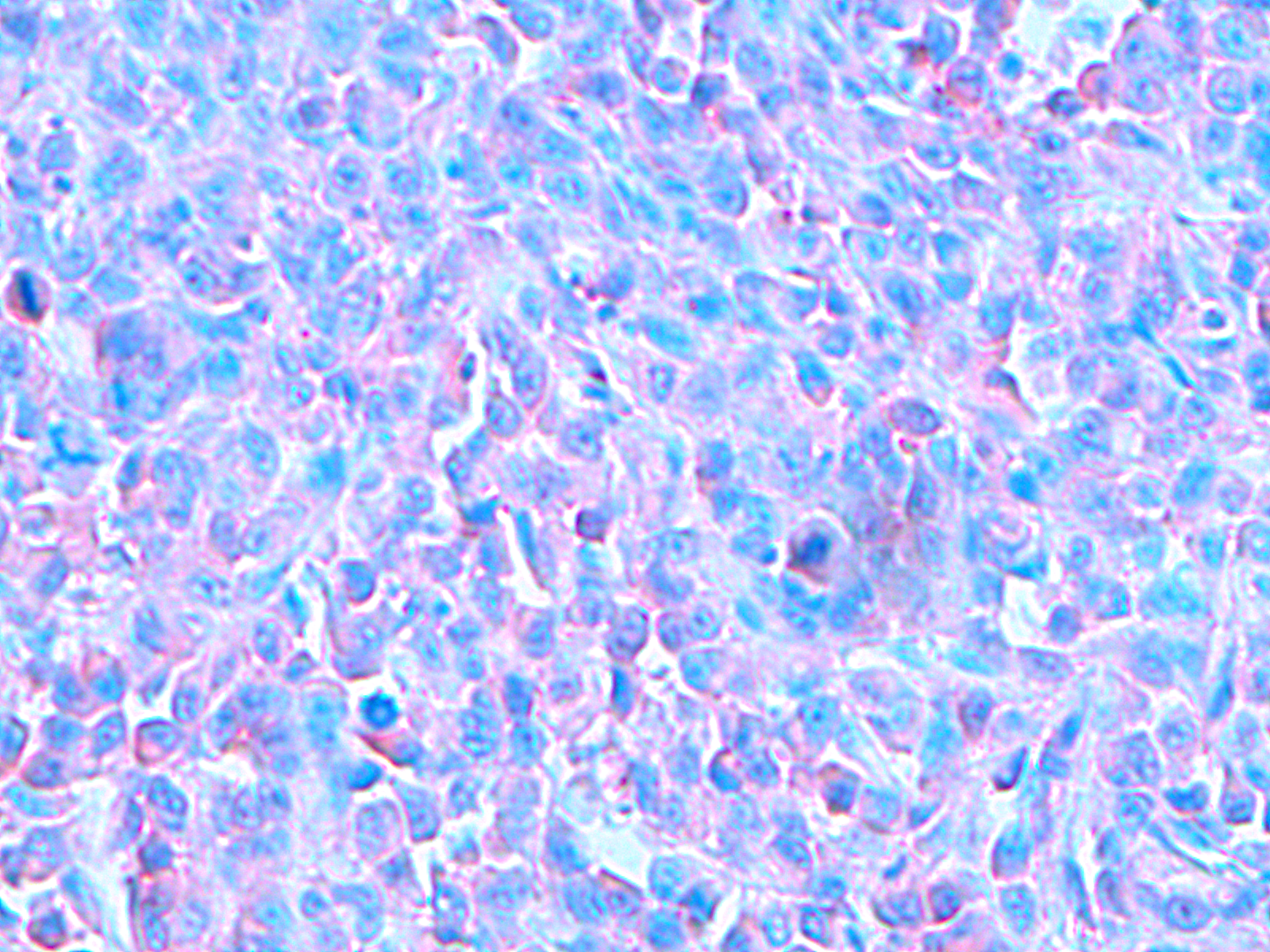

Supplement: S2 File — (ZIP) [file pone.0223138.s002.zip › S2_File/File S7 - 4A published_Bcl2 Resveratrol.tif]

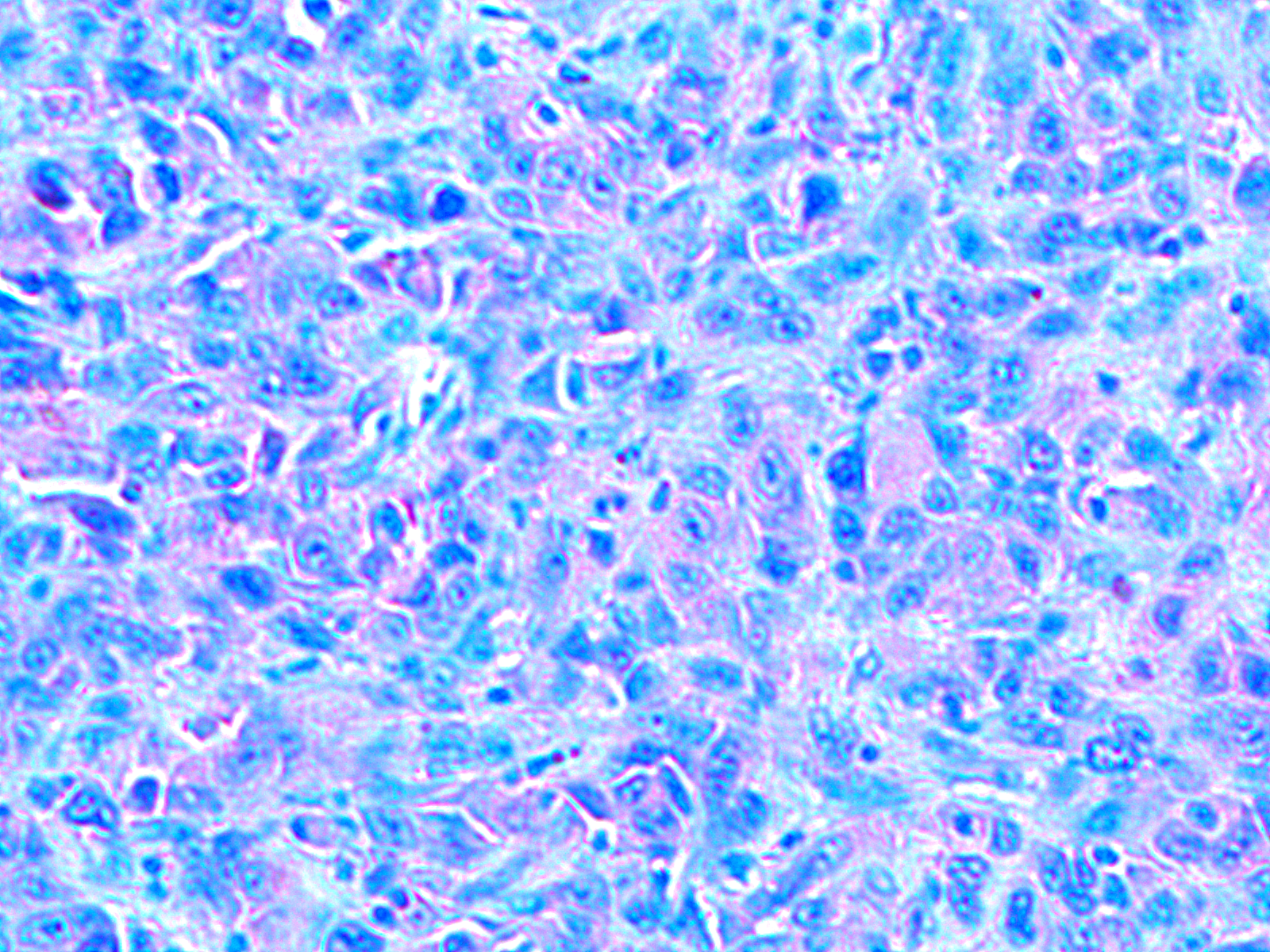

Supplement: S2 File — (ZIP) [file pone.0223138.s002.zip › S2_File/File S8 - 4A published_Bcl2 Resv + TRAIL.tif]

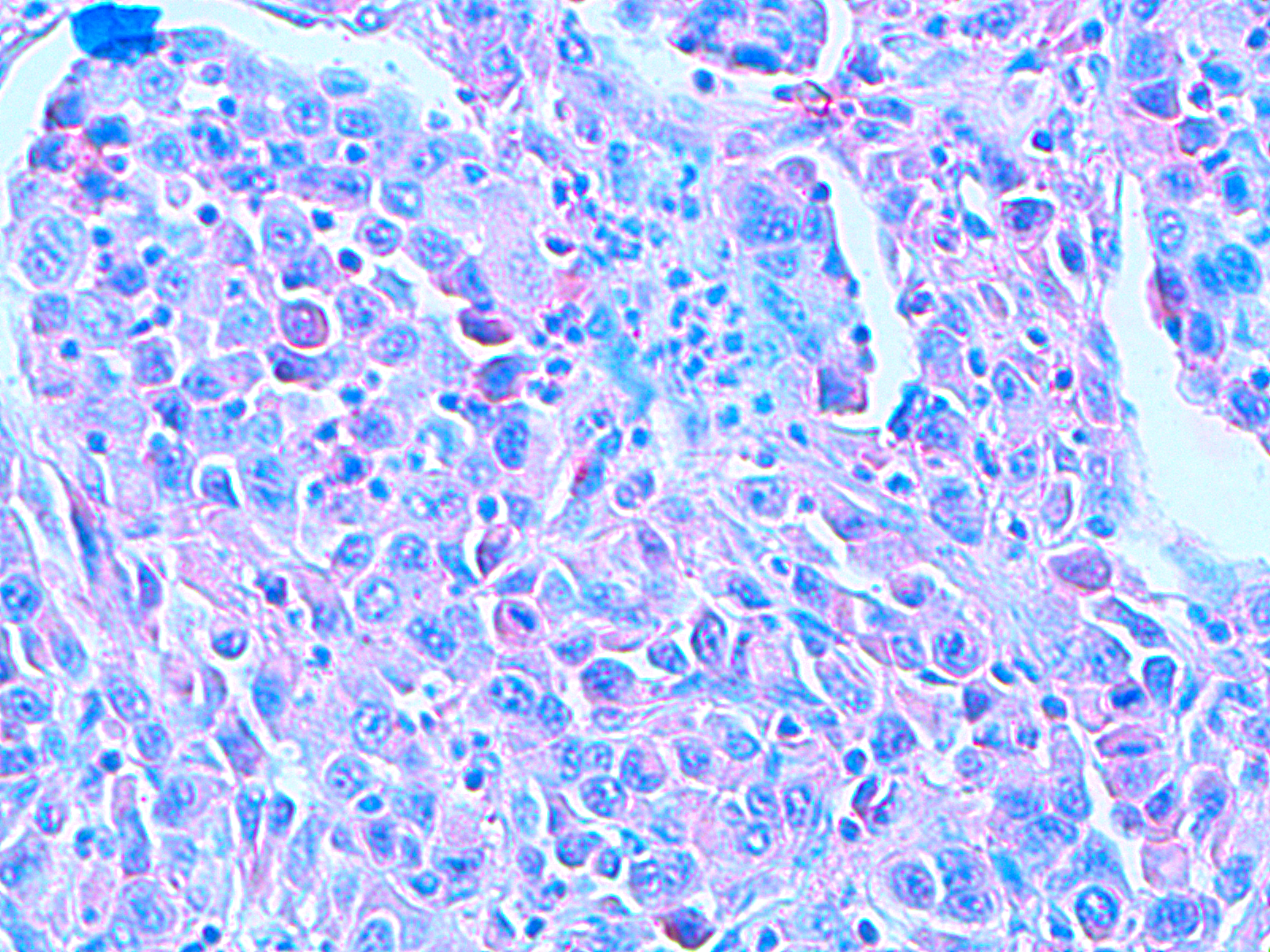

Supplement: S2 File — (ZIP) [file pone.0223138.s002.zip › S2_File/File_S19 - 4A published Bcl-2-TRAIL.tif]

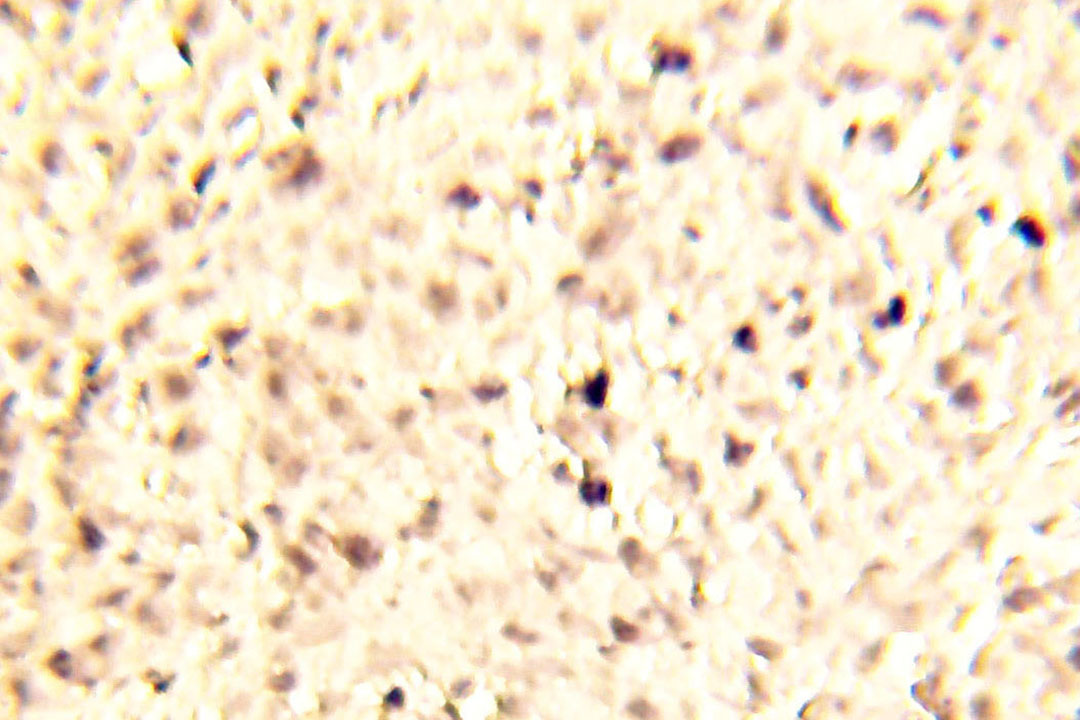

Supplement: S3 File — (ZIP) [file pone.0223138.s003.zip › S3_File/S2_File - 4A corrected Bax control.tif]

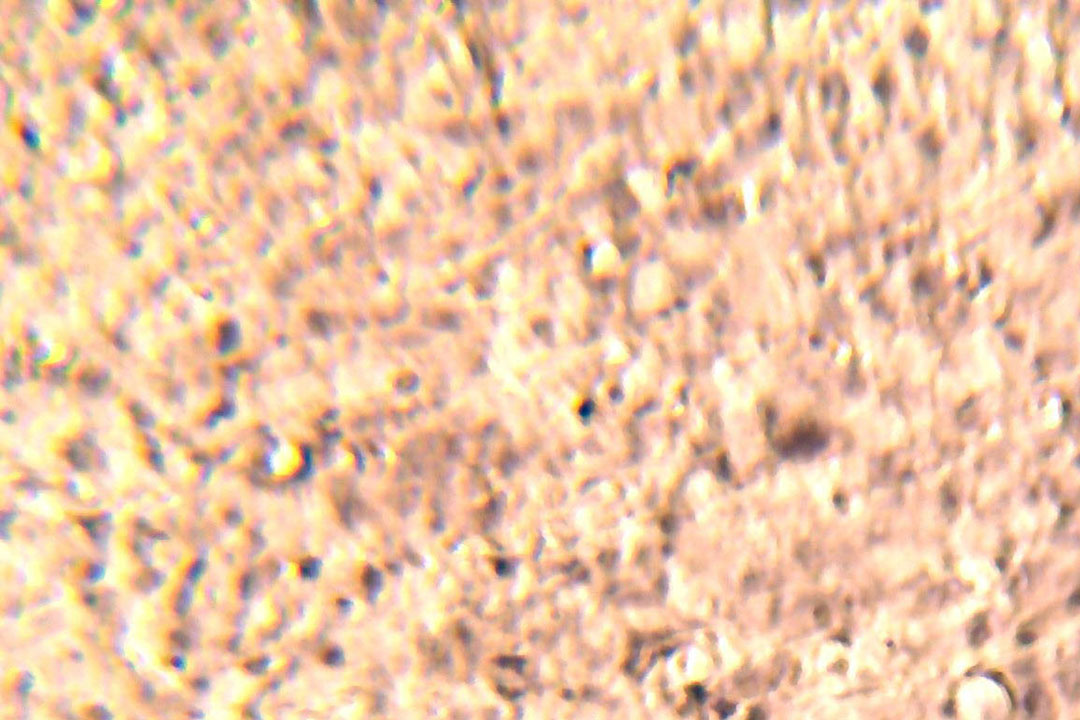

Supplement: S3 File — (ZIP) [file pone.0223138.s003.zip › S3_File/S3_File - 4A corrected Bax Resveratrol.tif]

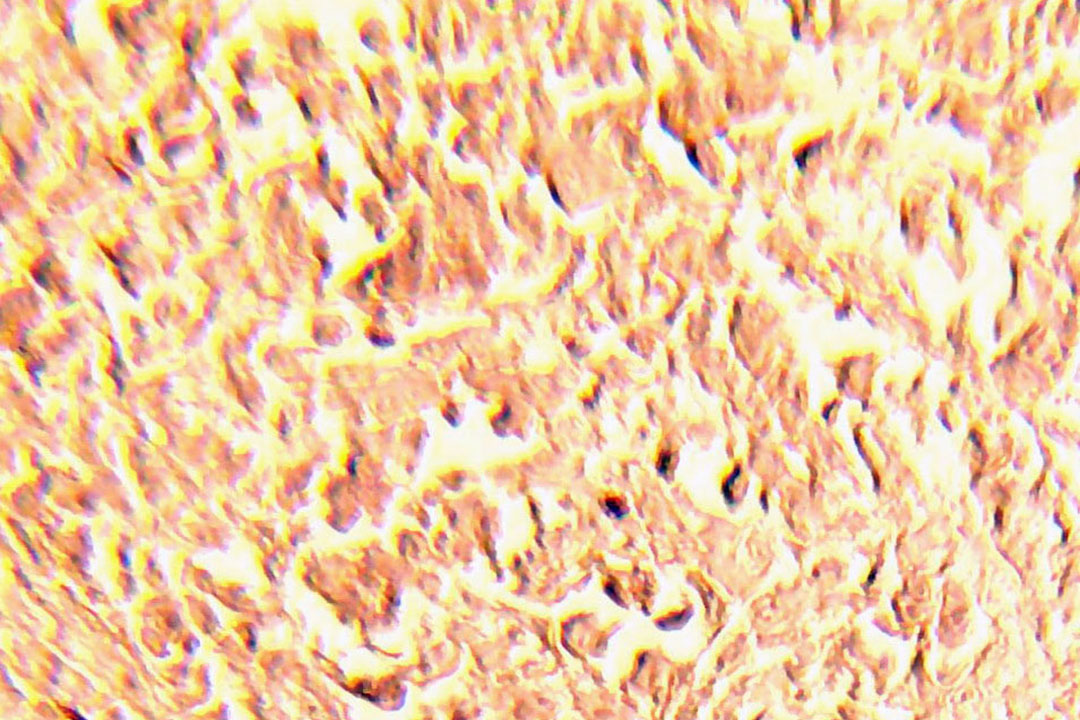

Supplement: S3 File — (ZIP) [file pone.0223138.s003.zip › S3_File/S4_File - 4A corrected Bax TRAIL.tif]

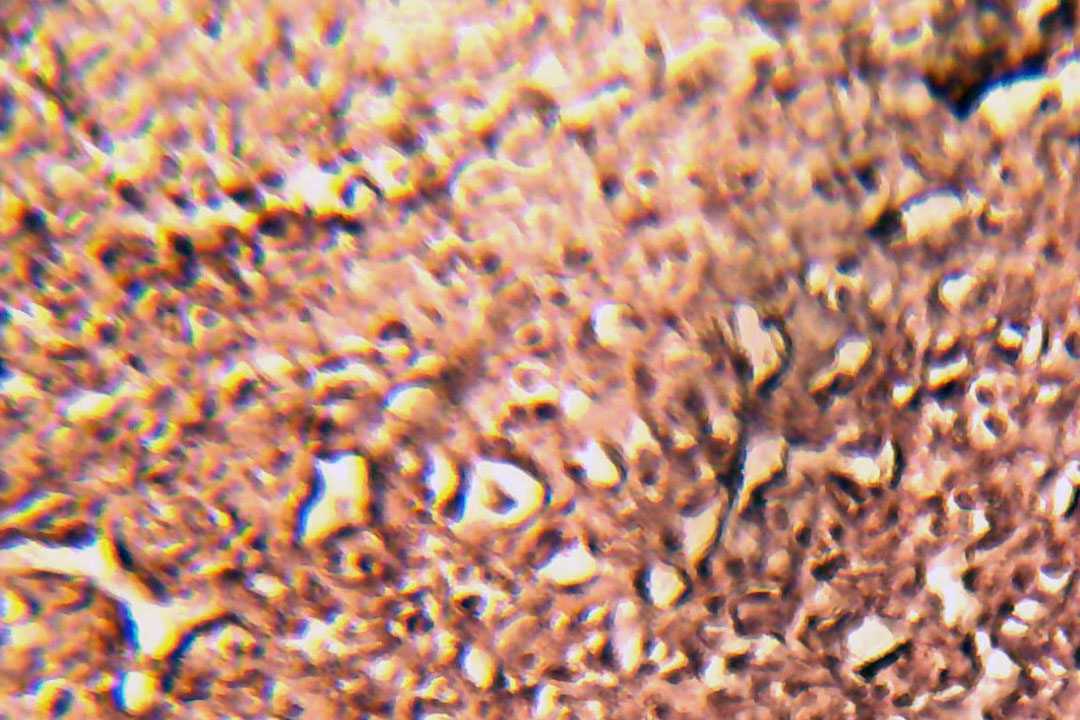

Supplement: S3 File — (ZIP) [file pone.0223138.s003.zip › S3_File/S5_File - 4A corrected Bax Resv+TRAIL.tif]

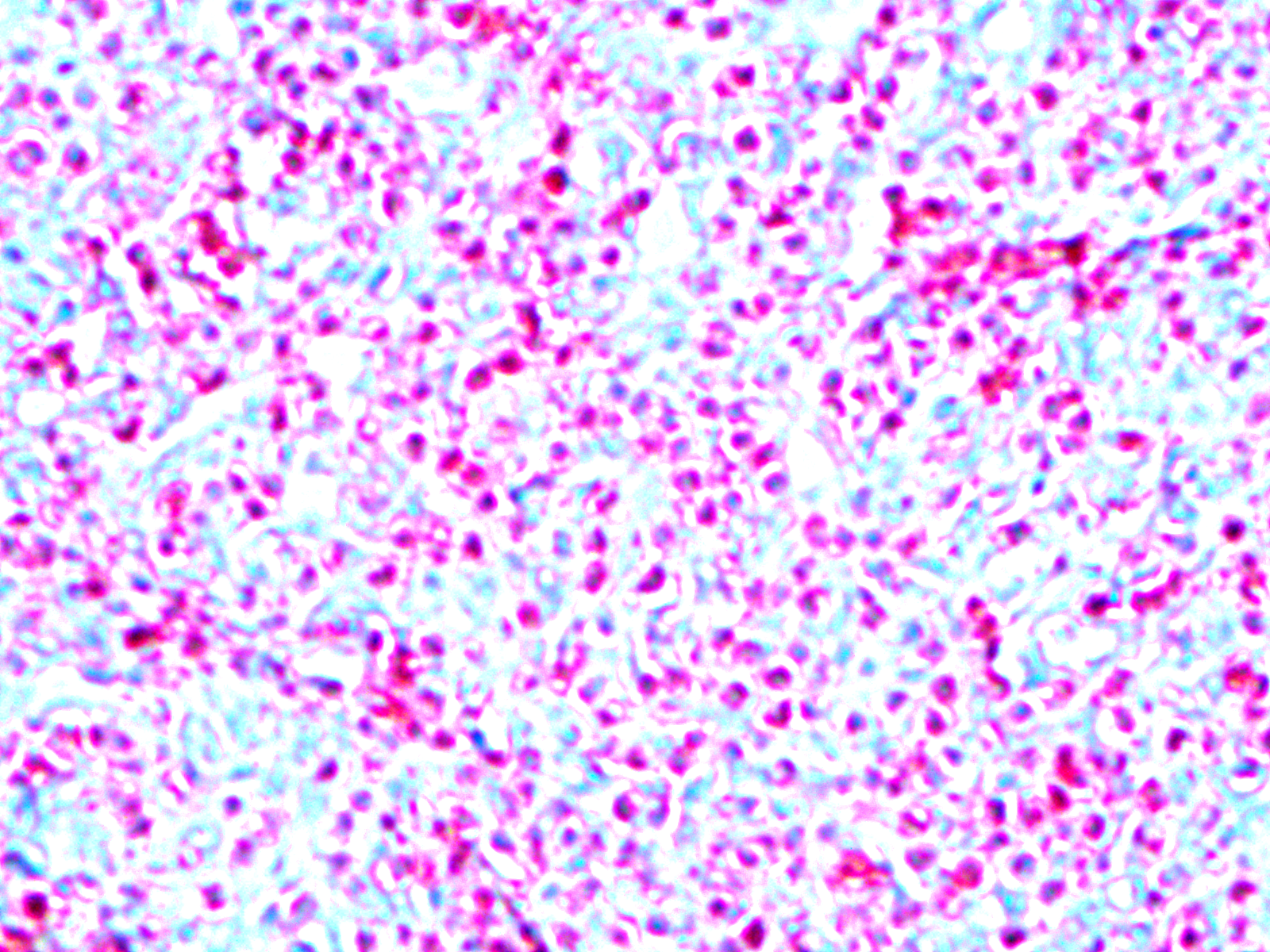

Supplement: S4 File — (ZIP) [file pone.0223138.s004.zip › S4_File/File S27 - 5A published MMP2 control.tif]

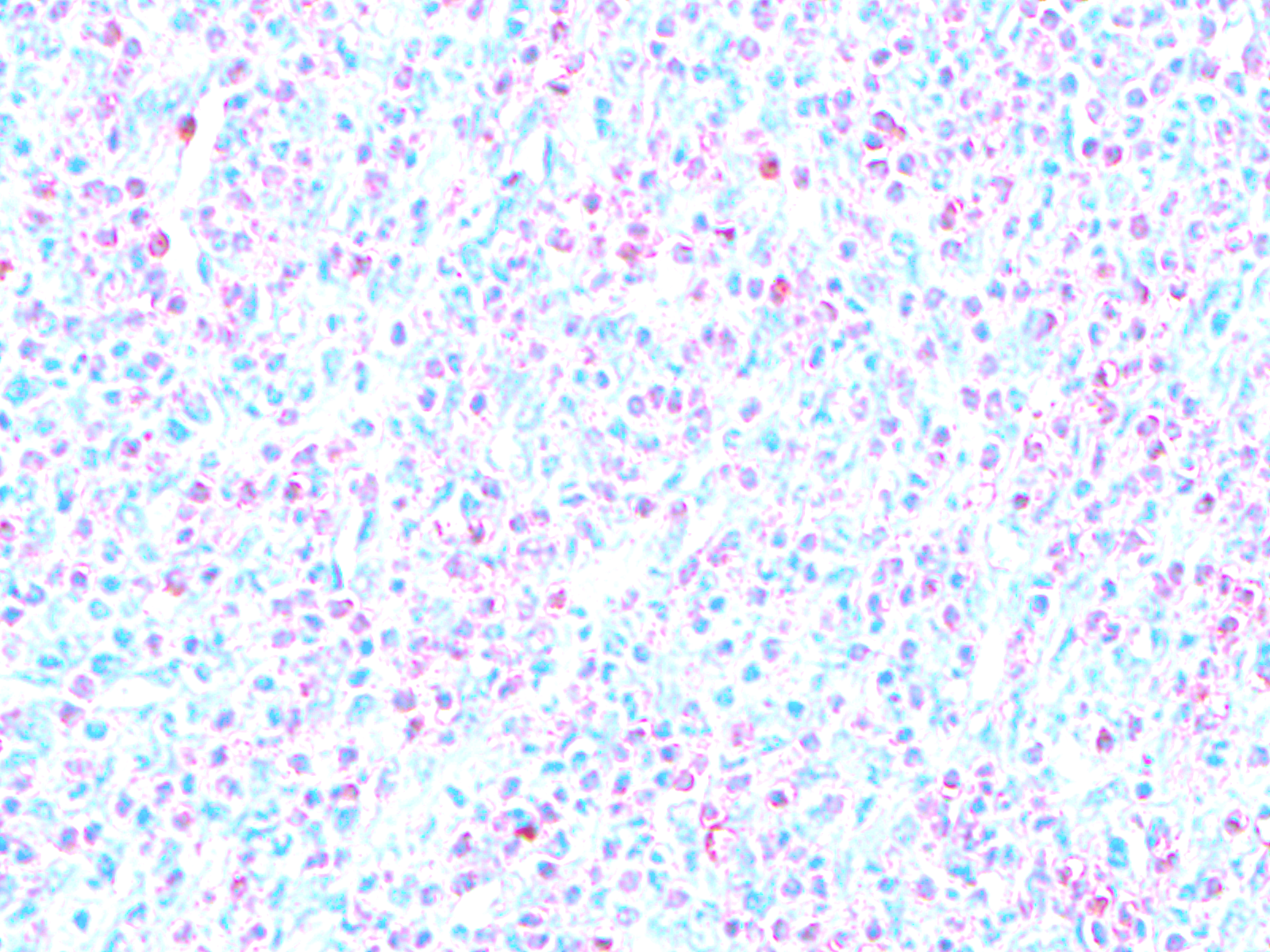

Supplement: S4 File — (ZIP) [file pone.0223138.s004.zip › S4_File/File S28 - 5A published MMP2 Resv.tif]

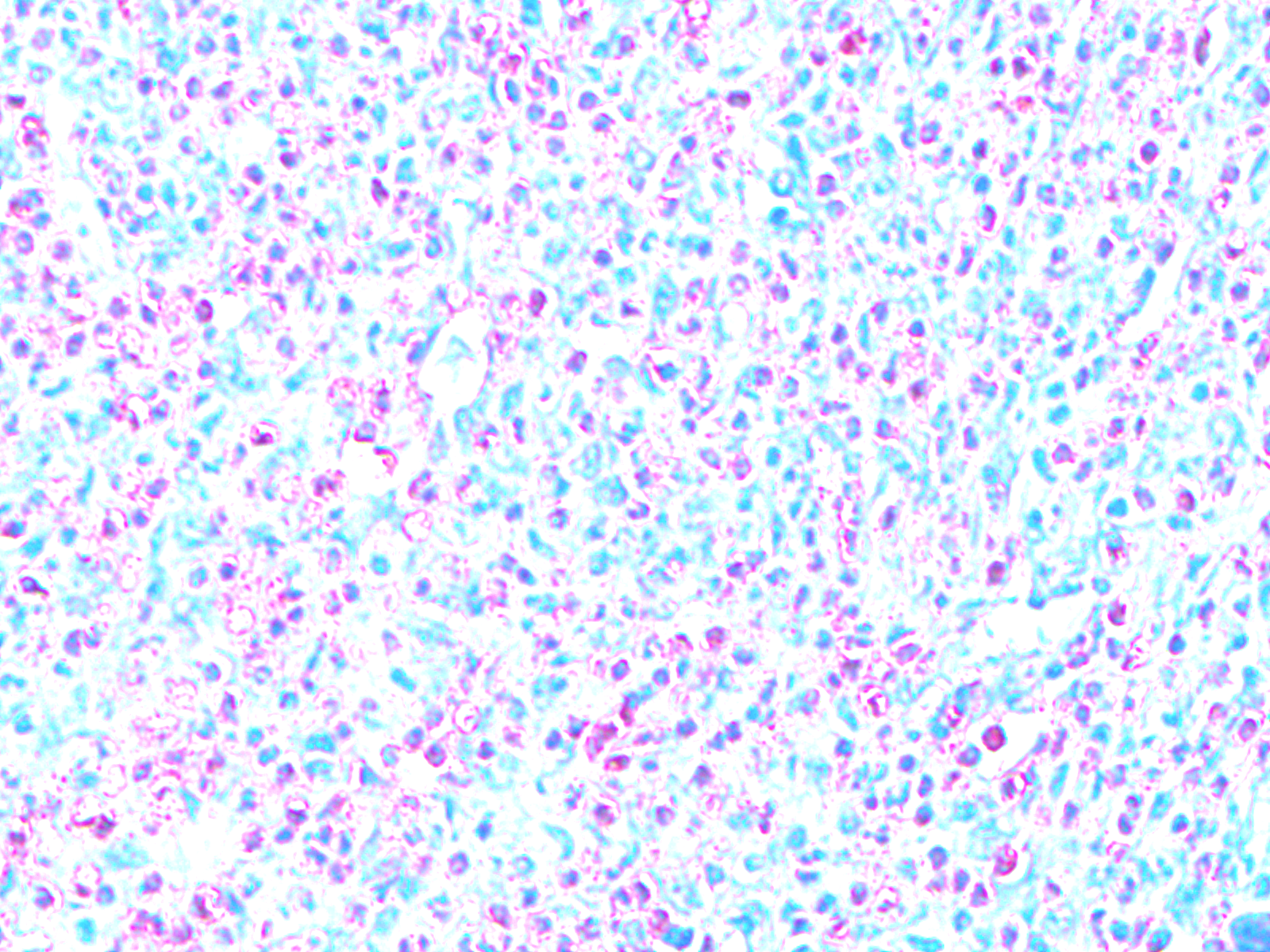

Supplement: S4 File — (ZIP) [file pone.0223138.s004.zip › S4_File/File S29 - 5A published MMP2 TRAIL.tif]

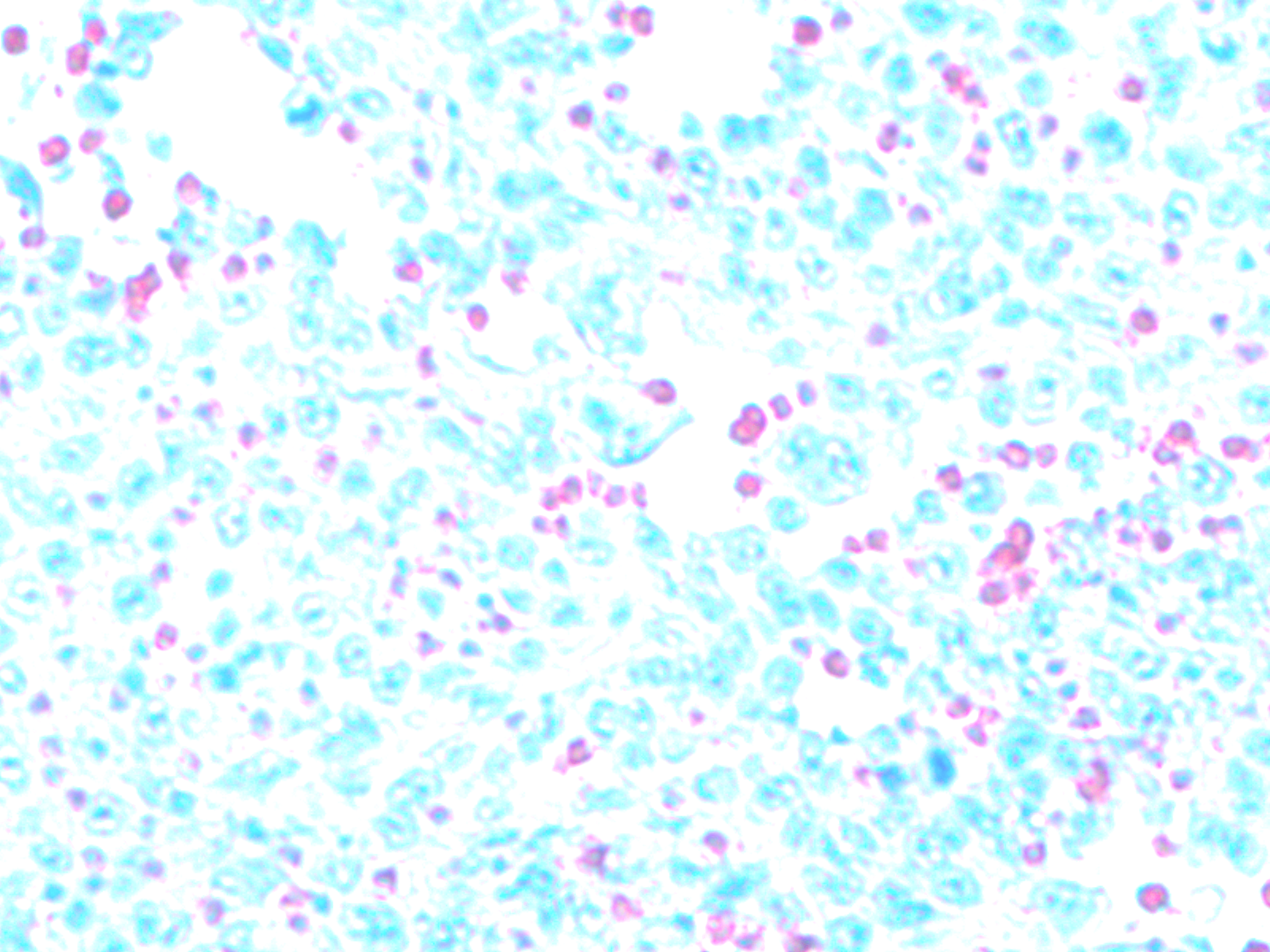

Supplement: S4 File — (ZIP) [file pone.0223138.s004.zip › S4_File/File S30 - 5A published MMP2 Resv TRAIL.tif]

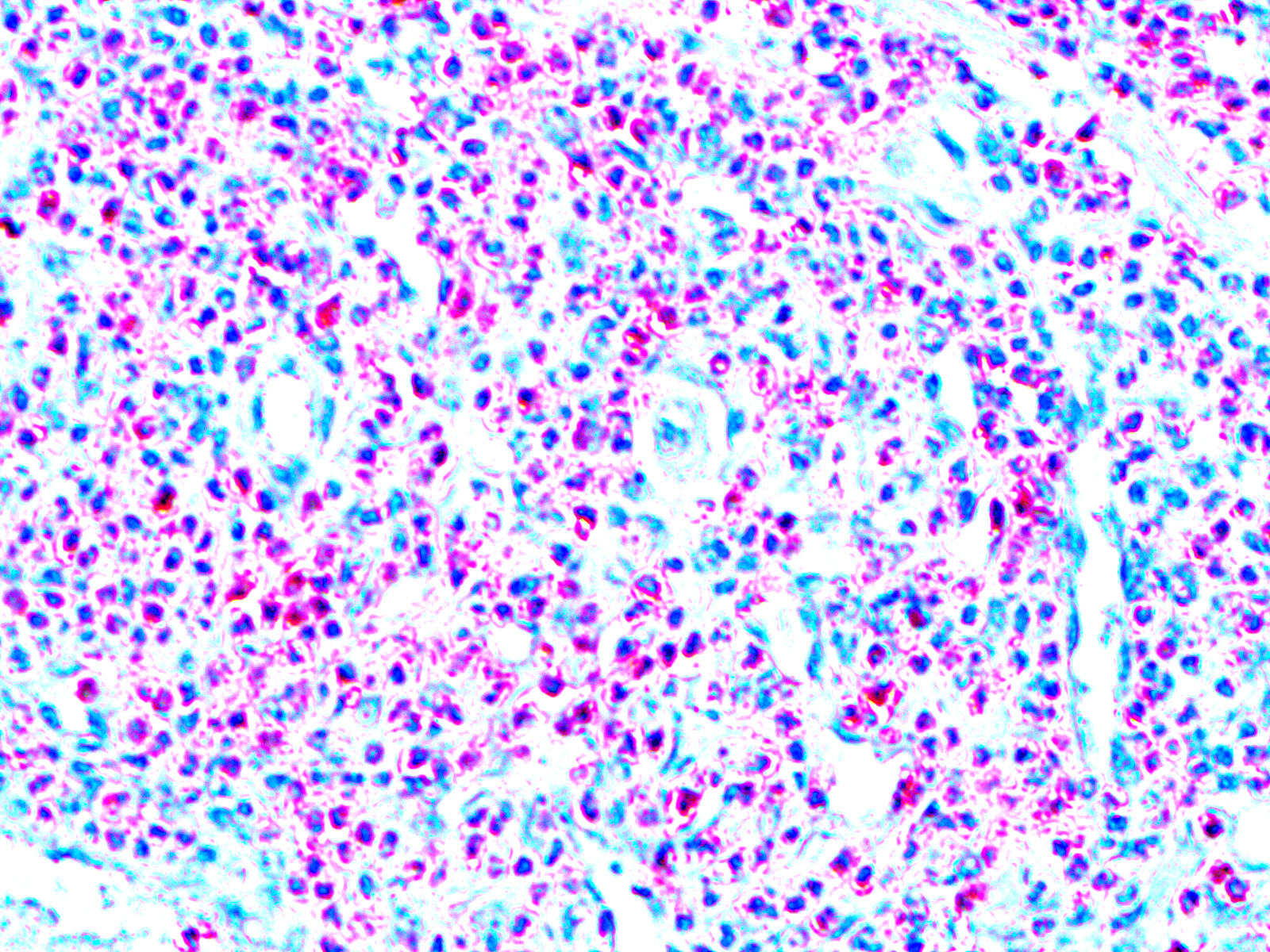

Supplement: S4 File — (ZIP) [file pone.0223138.s004.zip › S4_File/File S31 - 5A published MMP9 control.tif]

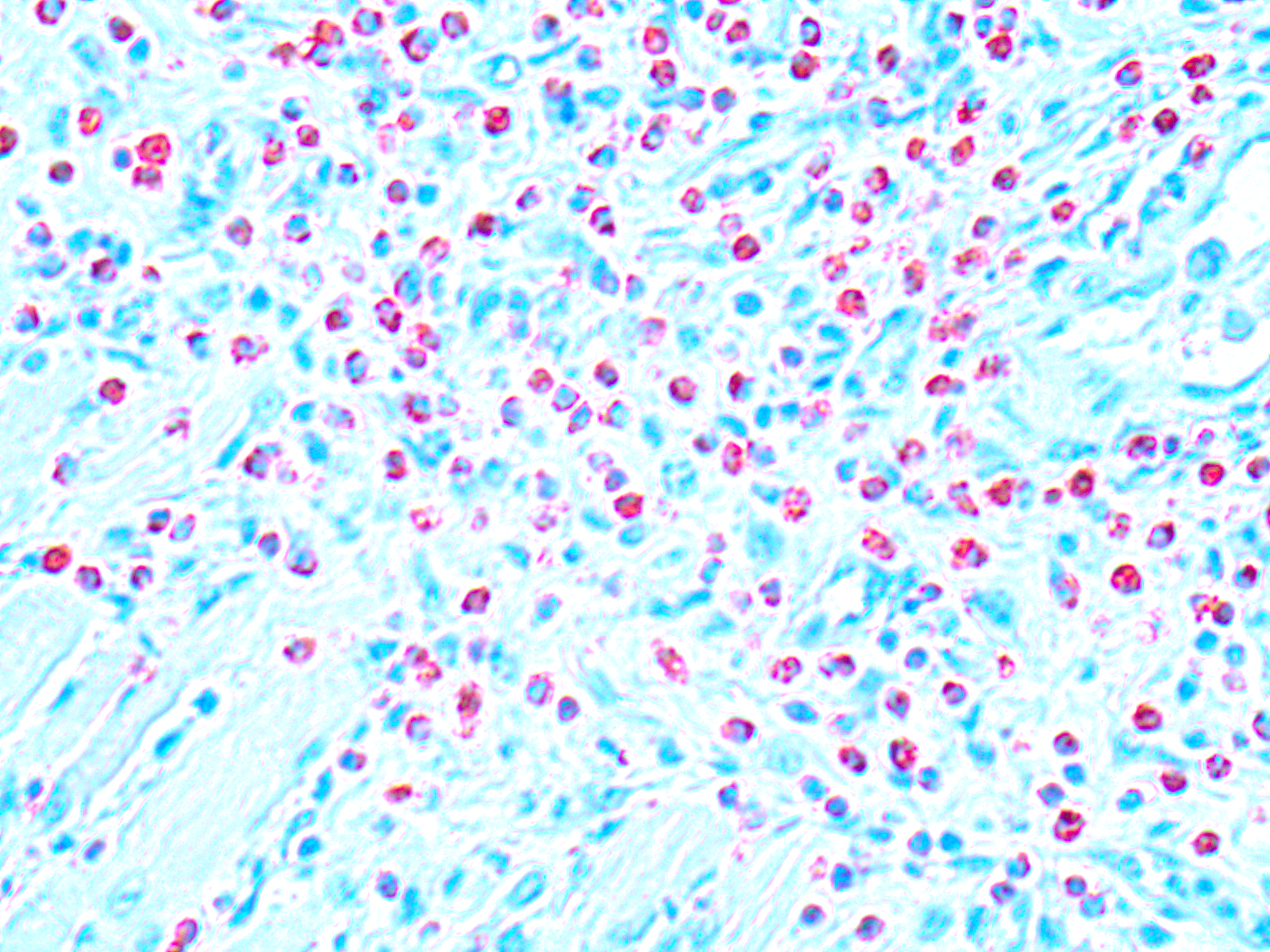

Supplement: S4 File — (ZIP) [file pone.0223138.s004.zip › S4_File/File S32 - 5A published MMP9 Resv.tif]

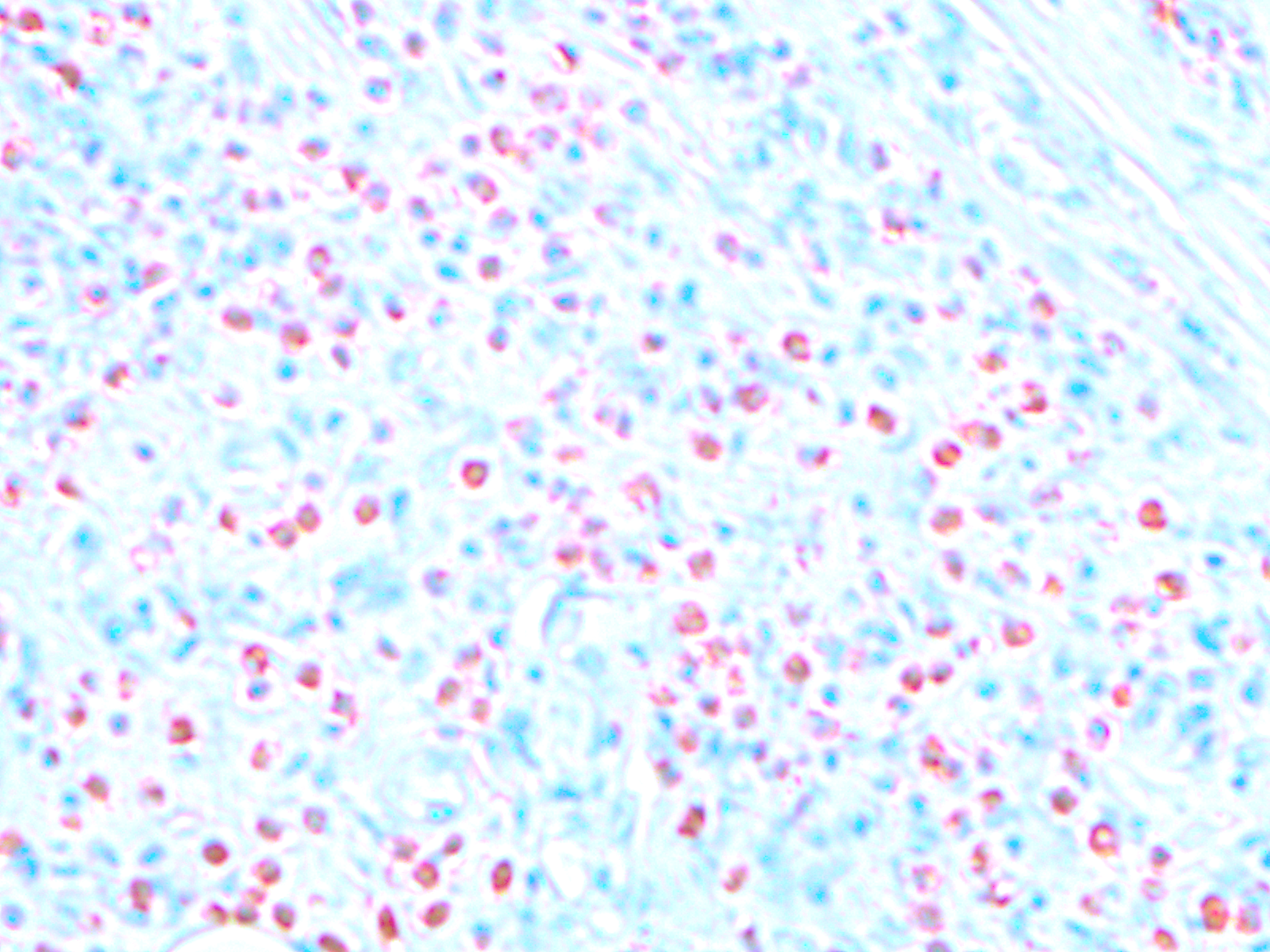

Supplement: S4 File — (ZIP) [file pone.0223138.s004.zip › S4_File/File S33 - 5A published MMP9 TRAIL.tif]

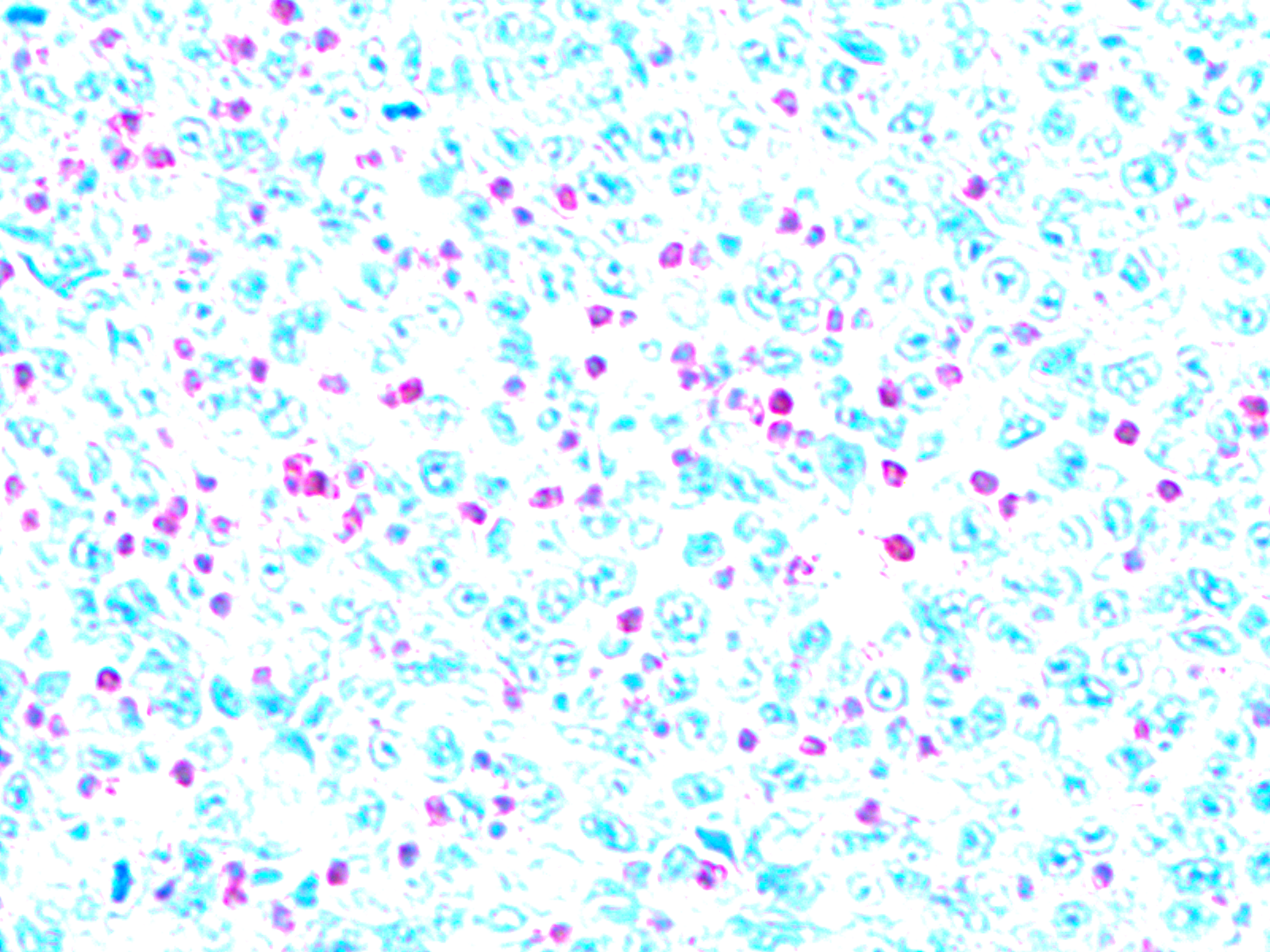

Supplement: S4 File — (ZIP) [file pone.0223138.s004.zip › S4_File/File S34 - 5A published MMP9 Resv TRAIL.tif]

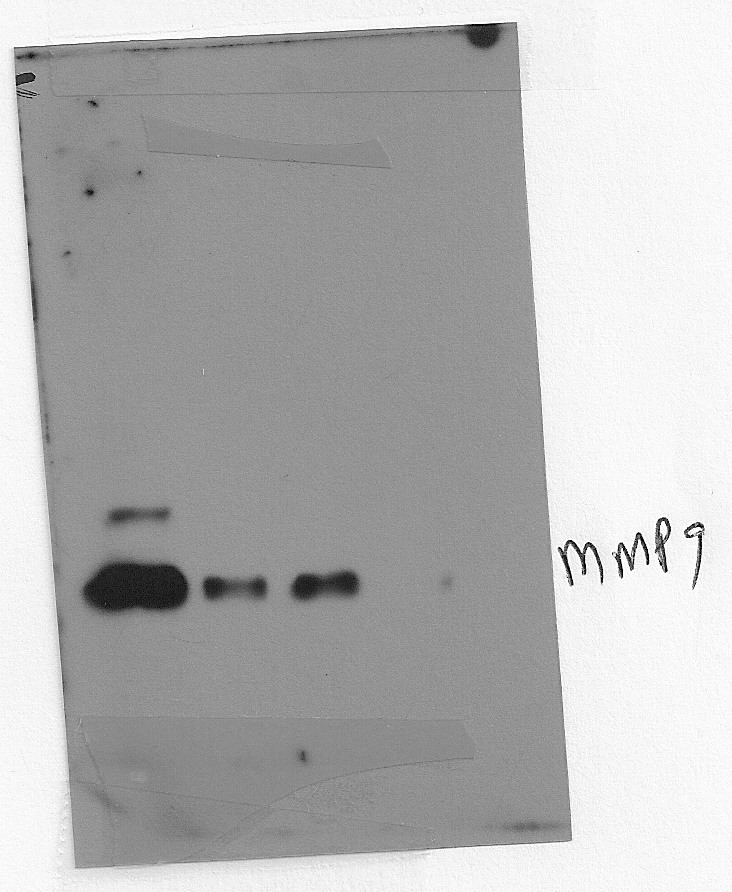

Supplement: S4 File — (ZIP) [file pone.0223138.s004.zip › S4_File/File S36 - 5C published MMP9 western blot.jpg]

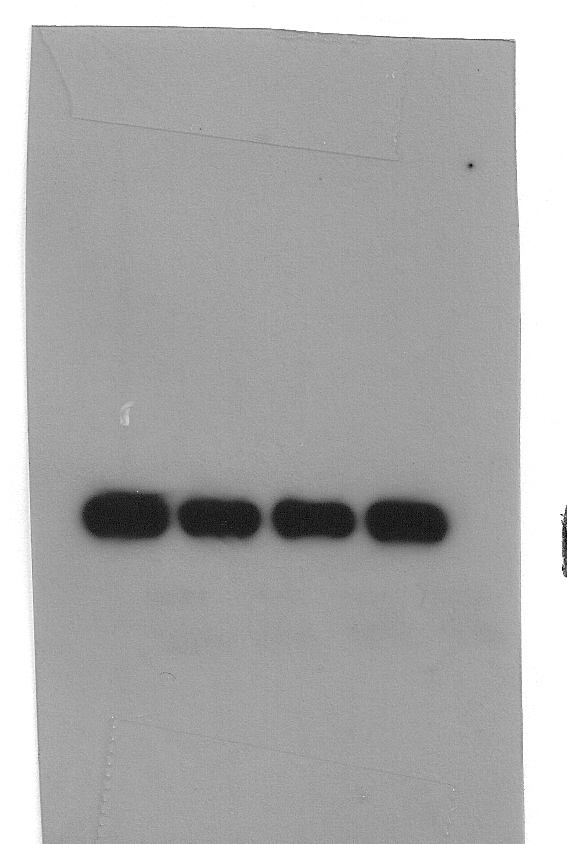

Supplement: S4 File — (ZIP) [file pone.0223138.s004.zip › S4_File/File S37 - 5C published b-actin western blot.jpg]

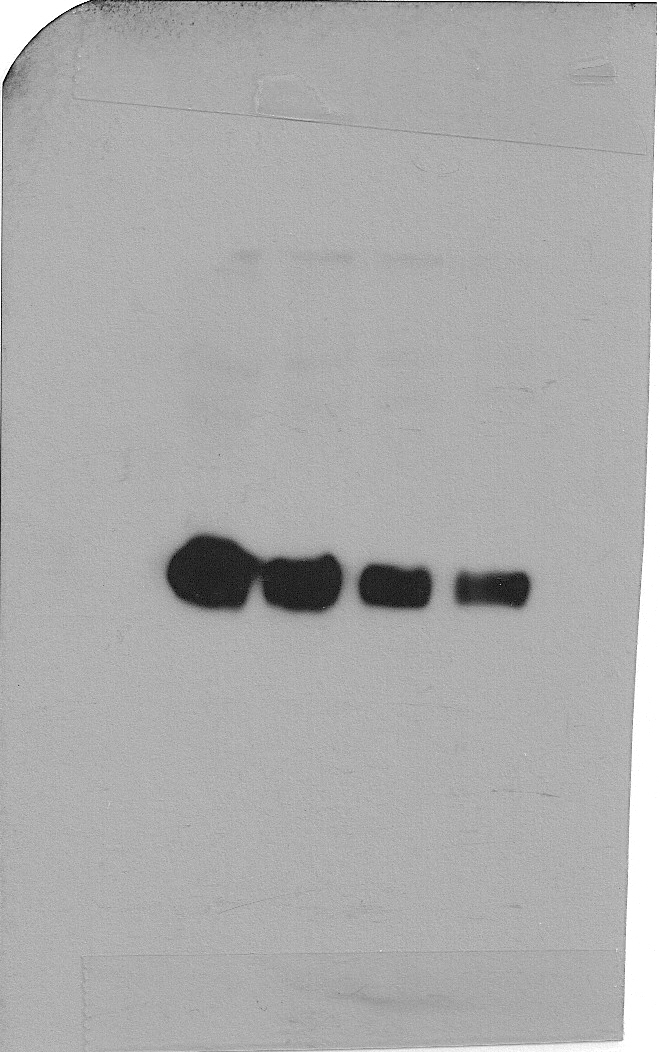

Supplement: S4 File — (ZIP) [file pone.0223138.s004.zip › S4_File/File S38 - 5C published MMP2 western blot.jpg]

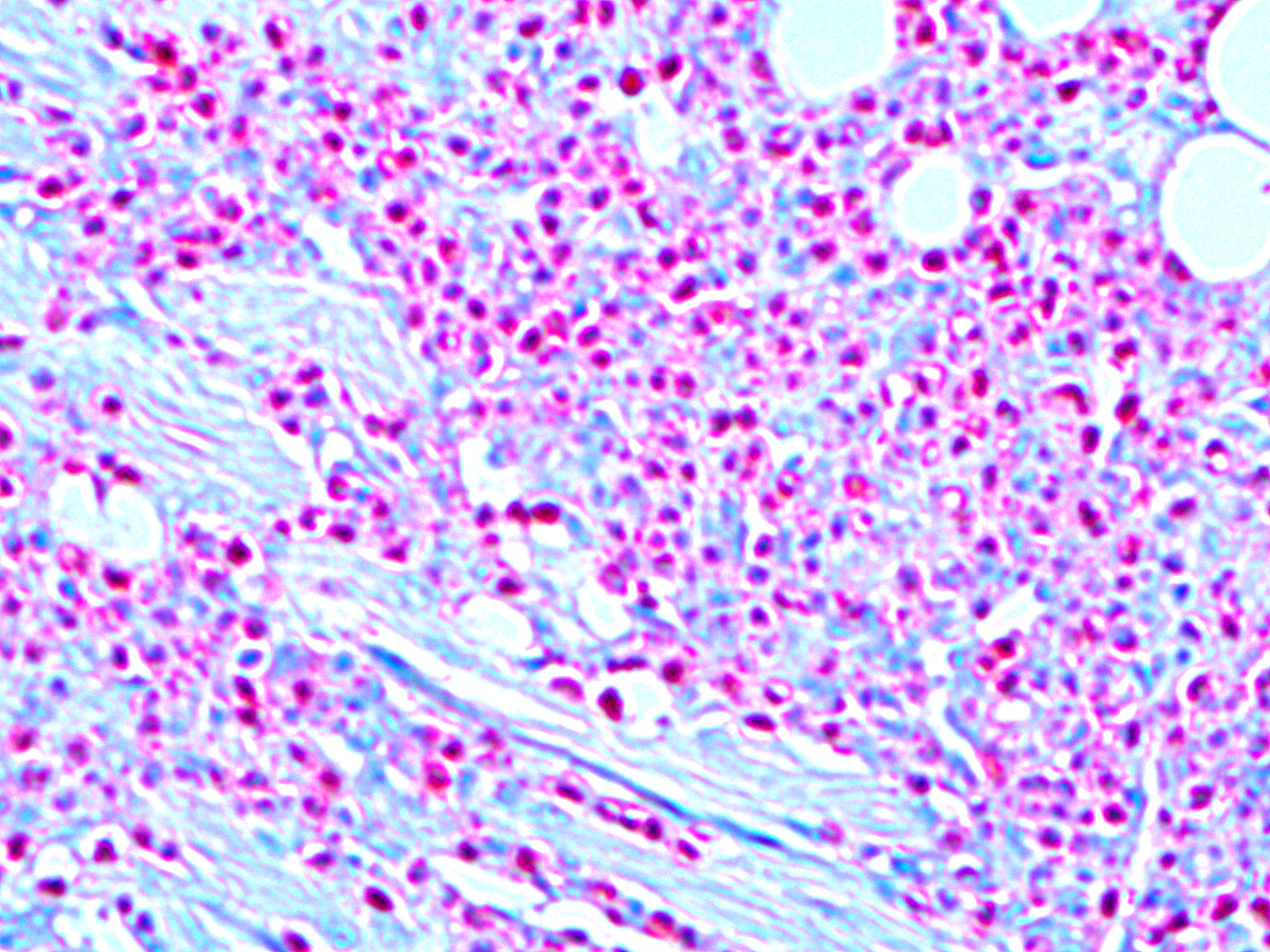

Supplement: S5 File — (ZIP) [file pone.0223138.s005.zip › S5_File/File S22 - 6B published -TRAIL control.tif]

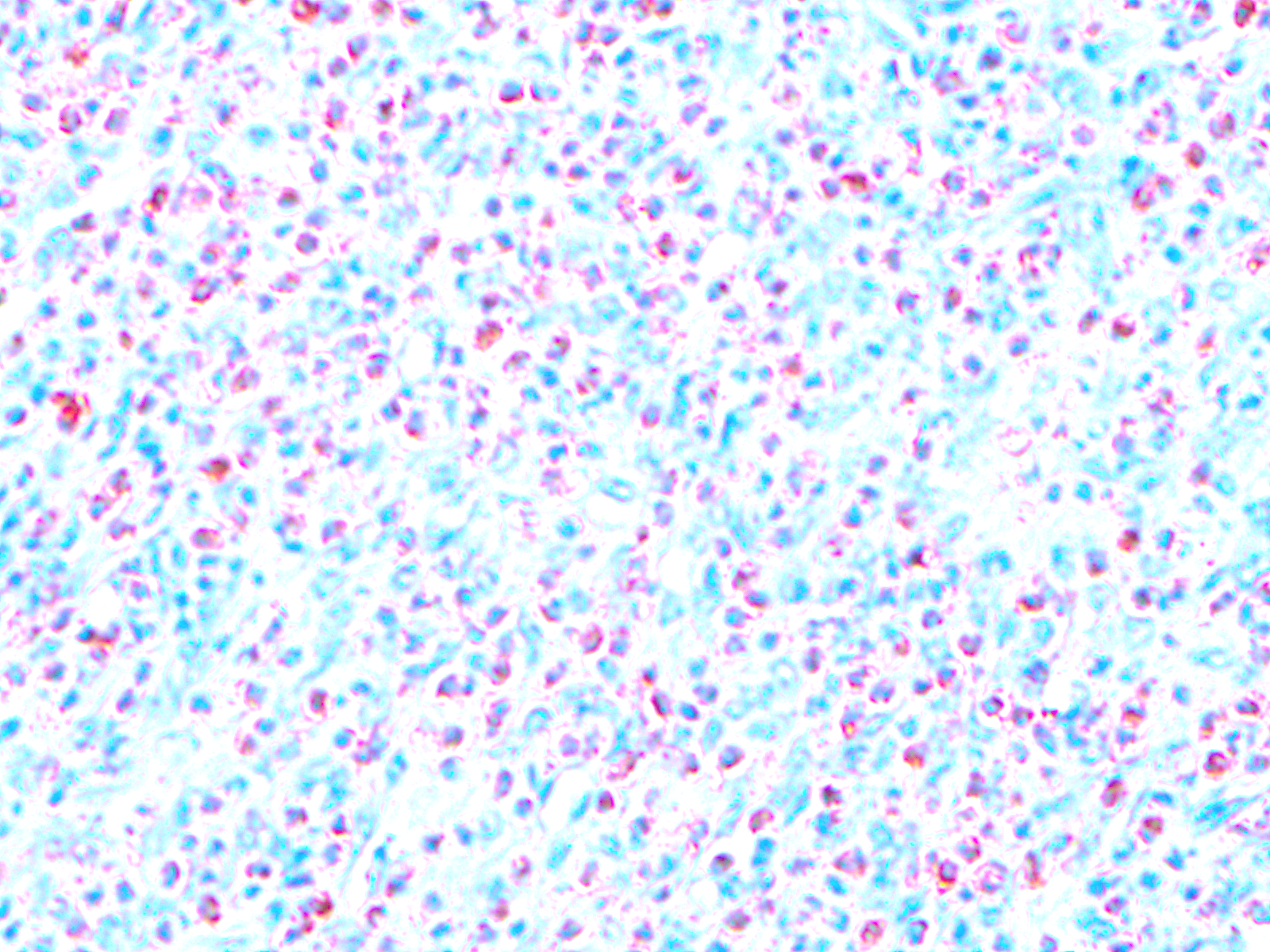

Supplement: S5 File — (ZIP) [file pone.0223138.s005.zip › S5_File/File S23 - 6B published -TRAIL Resv.tif]

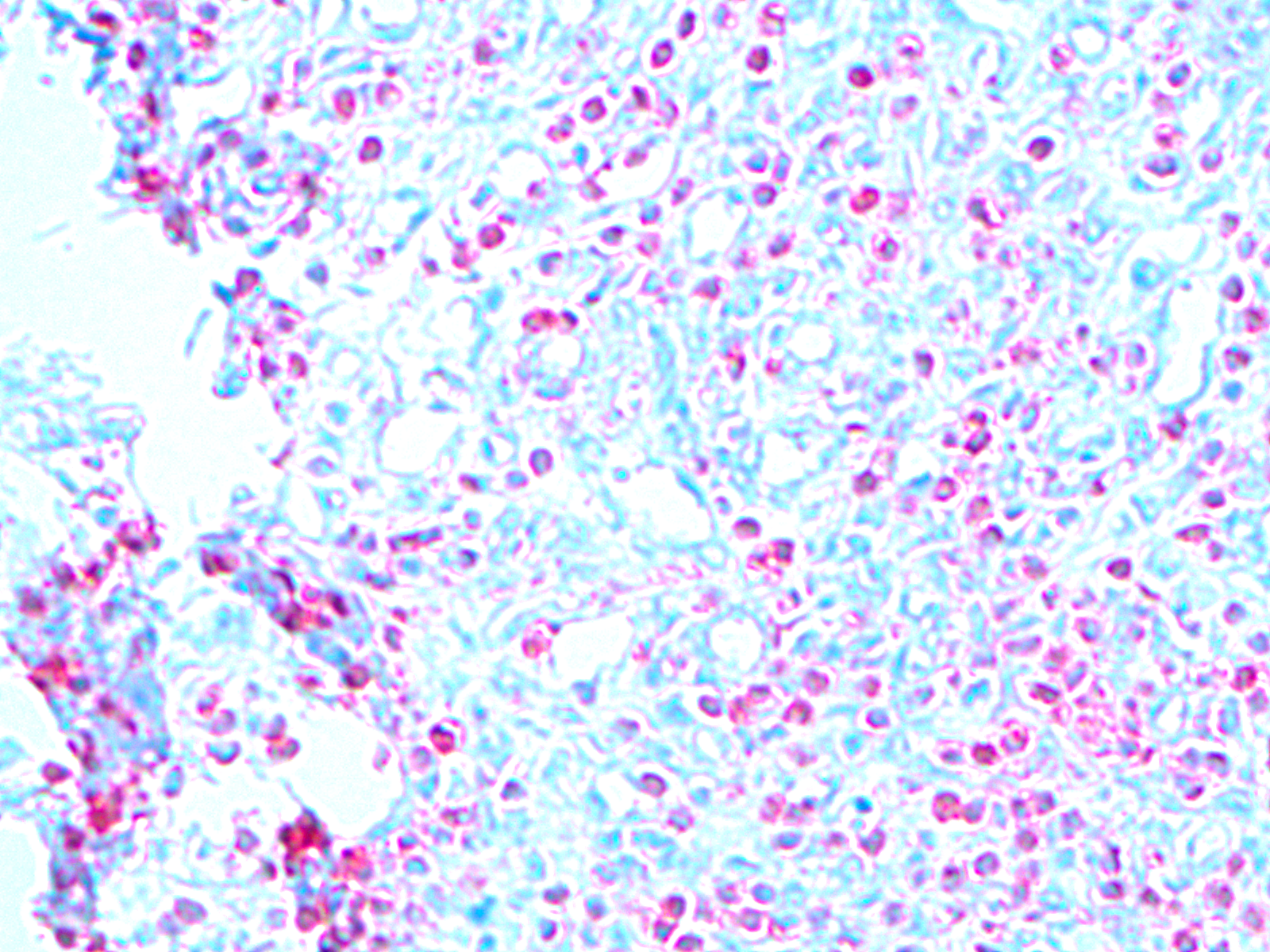

Supplement: S5 File — (ZIP) [file pone.0223138.s005.zip › S5_File/File S24 - 6B NEW +TRAIL Resv.tif]

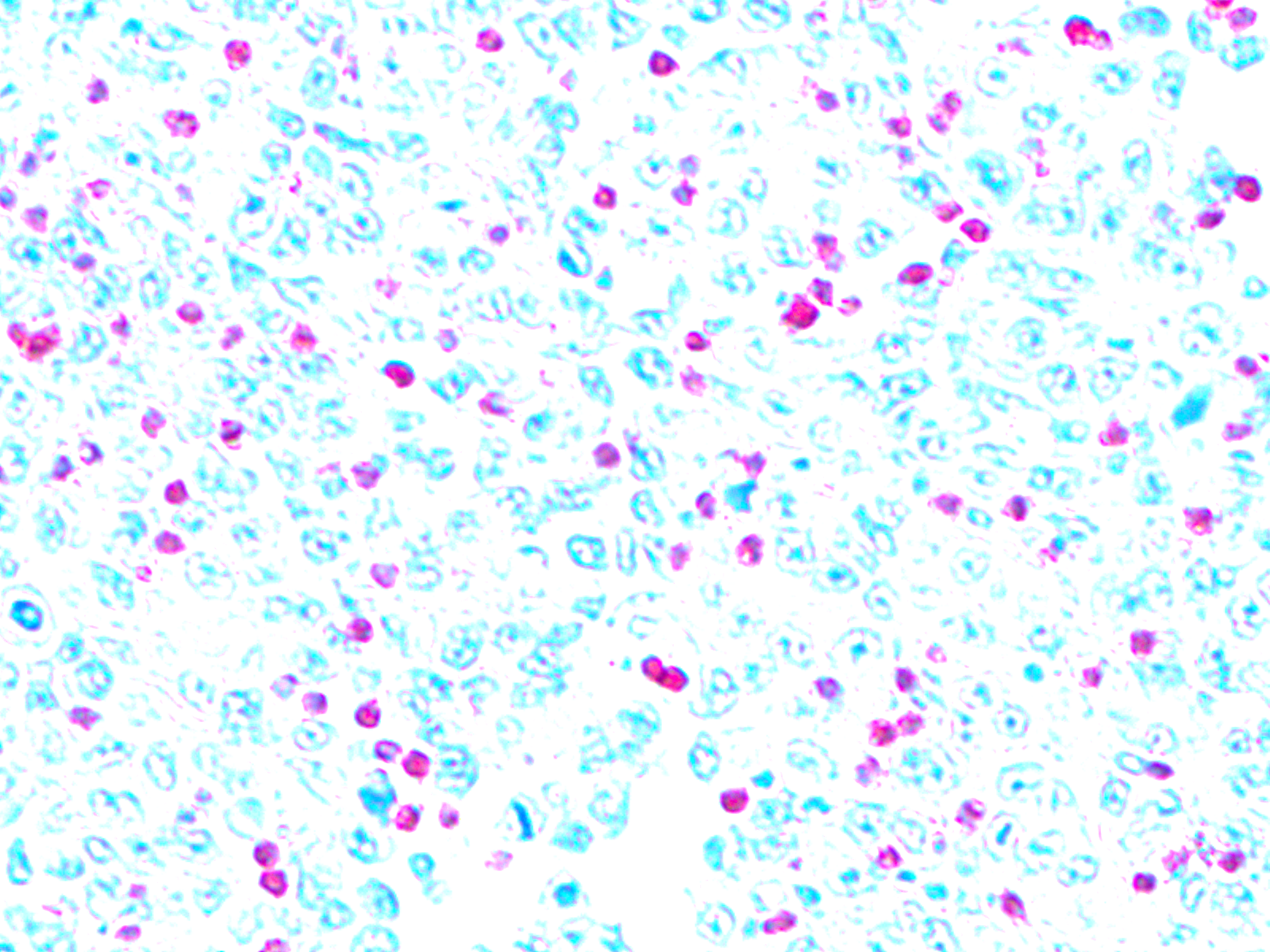

Supplement: S5 File — (ZIP) [file pone.0223138.s005.zip › S5_File/File S25 - 6B published +TRAIL Resv.tif]

## Slide 1
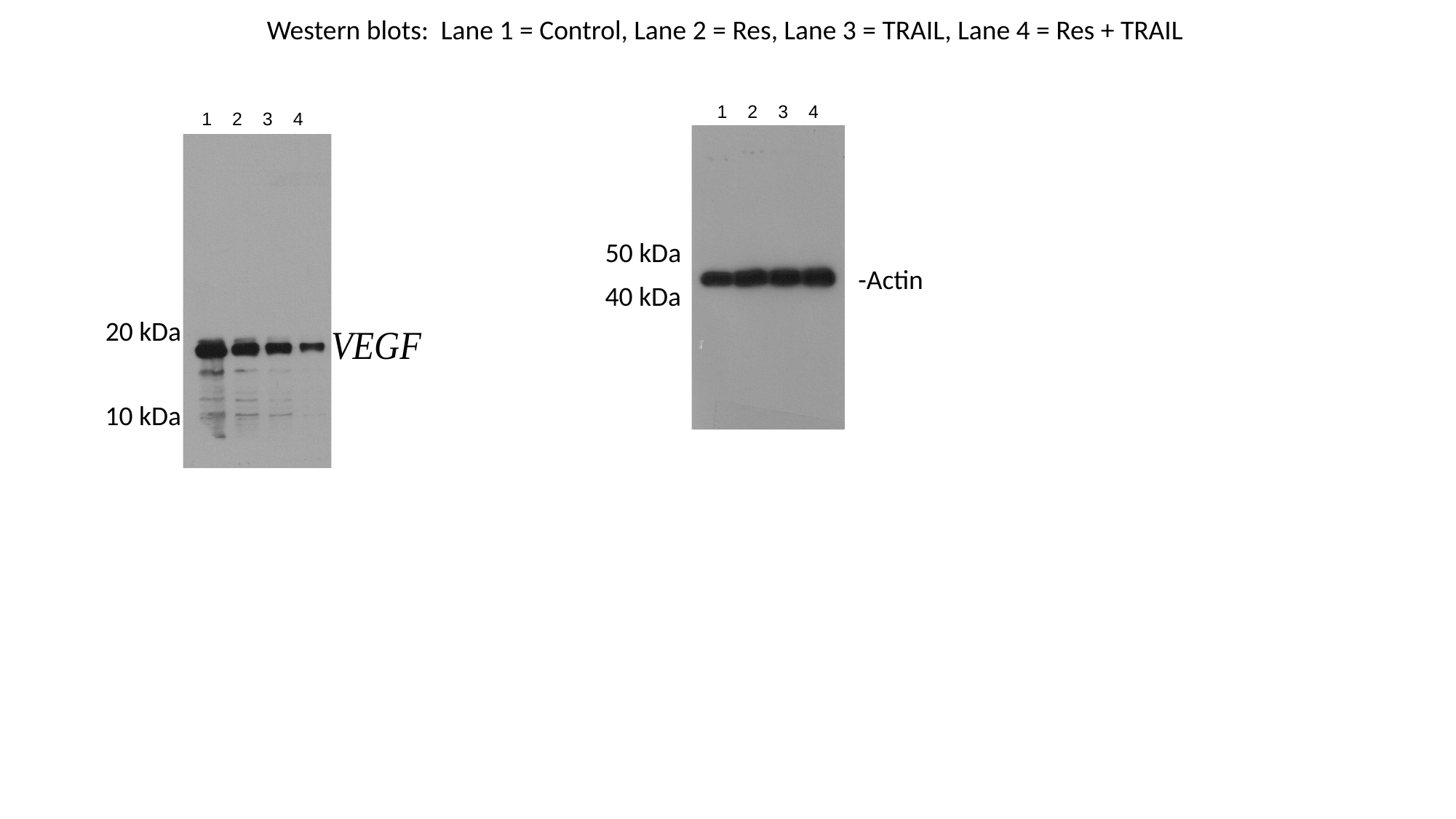

Western blots: Lane 1 = Control, Lane 2 = Res, Lane 3 = TRAIL, Lane 4 = Res + TRAIL
1 2 3 4
1 2 3 4
50 kDa
40 kDa
20 kDa
10 kDa

Supplement: S5 File — (ZIP) [file pone.0223138.s005.zip › S5_File/File S26 - 6B published western blots.pptx]
