# Supplementary material for: Expression of Concern: Resveratrol Enhances Antitumor Activity of TRAIL in Prostate Cancer Xenografts through Activation of FOXO Transcription Factor
Source: PLoS One. 2019 Sep 24;14(9):e0223138. doi: 10.1371/journal.pone.0223138 (PMC6759166; doi:10.1371/journal.pone.0223138)
Supplement: S2 File — (ZIP) [file pone.0223138.s002.zip › S2_File/File S1 - 4A published_western blots.pptx]

## Slide 1
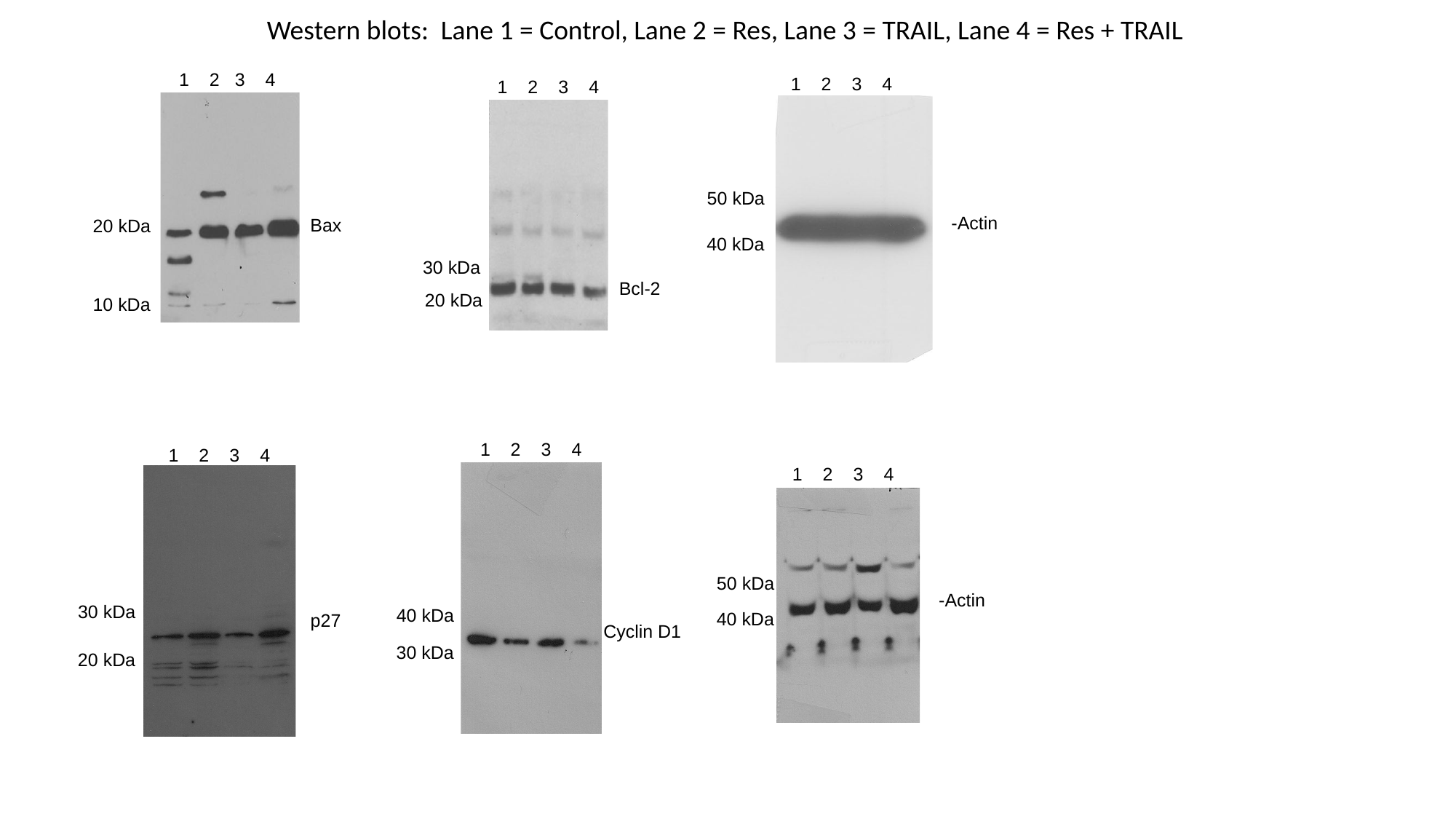

Western blots: Lane 1 = Control, Lane 2 = Res, Lane 3 = TRAIL, Lane 4 = Res + TRAIL
1 2 3 4
1 2 3 4
1 2 3 4
50 kDa
Bax
20 kDa
40 kDa
30 kDa
Bcl-2
20 kDa
10 kDa
1 2 3 4
1 2 3 4
1 2 3 4
50 kDa
30 kDa
40 kDa
40 kDa
p27
Cyclin D1
30 kDa
20 kDa
